# Cerium(III) Azolate Promoted CO_2_ Insertion

**DOI:** 10.1021/acs.inorgchem.5c03187

**Published:** 2025-11-05

**Authors:** Jonas Riedmaier, Cäcilia Maichle-Mössmer, Reiner Anwander

**Affiliations:** Institut für Anorganische Chemie, Eberhard Karls Universität Tübingen, Auf der Morgenstelle 18, Tübingen 72076, Germany

## Abstract

A series of sandwich cerium azolates (pyrazolates, triazolates, tetrazolates) has been synthesized via salt-metathesis (cerous precursor: [Cp*_2_CeCl_2_K­(thf)]_*n*_; Cp* = C_5_Me_5_) and protonolysis protocols (cerous precursors: Cp*_2_Ce­[N­(SiHMe_2_)_2_] or Cp*_2_Ce­[N­(SiMe_3_)_2_]) and their carboxophilicity has been probed. The sandwich and half-sandwich complexes Cp*_2_Ce­(pz^Me,Me^) and Cp*Ce­(pz^Me,Me^)_2_(thf)_2_ show exhaustive CO_2_ insertion into the pyrazolato moieties, which leads to bis­(carbamato)-bridged dimeric complexes. Complex Cp*_2_Ce­(pz^Ph,Ph^), featuring the sterically more demanding and less nucleophilic diphenylpyrazolato ligand inserts only one molecule CO_2_ per two metal centers, forming the asymmetrically bridged complex Cp*_2_Ce­(μ-pz^Ph,Ph^·CO_2_)­CeCp*_2_(pz^Ph,Ph^). The triazolato derivatives Cp*_2_Ce­(tz^Me,Me^)­(dmap) and Cp*_2_Ce­(tz^Ph,Ph^) indicate CO_2_ insertion only for the DMAP-free complex. Utilizing the nitrogen-richer 5-phenyltetrazole resulted in the formation of the trimer [Cp*_2_Ce­(tet^Ph^)]_3_, which does not insert CO_2_ at ambient temperature. The absorption and electrochemical properties of the compounds were investigated and their catalytic activity in the cycloaddition of CO_2_ and propylene oxide was examined.

## Graphical abstract

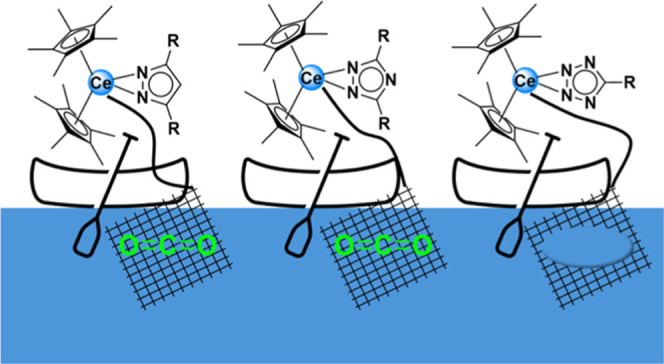

## Introduction

Carbon capture and storage (CCS) as well as carbon capture and utilization (CCU) display the most commonly pursued strategies to combat the emission of carbon dioxide as the most common greenhouse gas.^1–4^ Industrial CCS is currently accomplished by using aqueous amines.^5–7^ The favorable molecular interaction of the nucleophilic amine nitrogen with CO_2_ is the basis/key for this technology. More recently, this approach has been also successfully exploited in amine-containing ionic liquids or high surface materials like metal–organic frameworks (MOF) or porous silica.^8,9^ Rare-earth metals exhibit a great compatibility with CO_2_ due to their high oxophilicity and pronounced Lewis acidity of the trivalent metal cations.^10^ Recently, we observed the reversible insertion of CO_2_ into homoleptic cerium dimethylpyrazolates yielding Ce^III^_4_(pz^Me,Me^·CO_2_)_12_ and Ce^IV^(pz^Me,Me^·CO_2_)_4_ (pz^Me,Me^ = 3,5-dimethylpyrazolato, max. 25 wt % CO_2_).^11^ This concept was also applied to the lighter metals magnesium, aluminum and titanium as well as the rare-earth metals scandium and yttrium.^12–14^

The present study aimed at a deeper molecular understanding of the carboxophilicity of rare-earth-metal azolato moieties. We stuck to cerium as the most abundant metal center and the feasibility of Ce­(III)/Ce­(IV) redox events. Further, we chose the “inert” sandwich entity [Cp*_2_Ce]^+^ (Cp* = pentamethylcyclopentadienyl) ensuring one reactive Ce­(III)-azolato site. The scope of the azole pool should not be limited to differently substituted pyrazoles Hpz^R,R^ but be extended to triazoles (Htz^R,R^ = 3,5-*R*,*R*-1,2,4-triazole) and 5-phenyltetrazole (Htet^Ph^).

The class of molecular rare-earth-metal azolates with three or more nitrogen atoms in the 5-membered ring has not been exhaustively investigated yet. In 2006 the group of Müller-Buschbaum synthesized [Yb­(tz^H,H^)_3_]_∞_ and [Eu_2_(tz^H,H^)_5_(Htz^H,H^)_2_]_∞_ via direct reaction of the metals with Htz^H,H^.^15^ Another example is the homoleptic complex La­(tz^Ph,Ph^)_3_(thf)_3_ obtained by protonolysis of La­[N­(SiMe_3_)_2_]_3_ with Htz^Ph,Ph^.^16^ Rare-earth-metal complexes bearing a 1,2,3-triazolato ligand (trz) include [Ln­(trz^H,H^)_3_]_∞_ (Ln = Gd–Lu)^17,18^ as well as Cp*_2_Ln­(trz^SiMe3,*t*Bu^)­(NC*t*Bu) (Ln = La, Sm) obtained from [Cp*_2_Ln­(CNN­(SiMe_3_))]_2_ and nitriles.^19^ Rare-earth-metal tetrazolates are featured mainly by MOFs or complexes with multidentate tetrazolato ligands.^20–22^ To access alkali-metal tetrazolate precursors, deprotonation of the proligand with MOH (M = Li, Na, K, Rb, Cs), KH or M_2_CO_3_ (M = K, Cs) was used.^23–25^ The group of Winter also reported Nb­(pz^*t*Bu,*t*Bu^)_3_(tet^Ph^)_2_ and Ta­(pz^*t*Bu,*t*Bu^)_3_(tet^Ph^)Cl as well as Cr­(*t*BuNC­(CH_3_)­N*t*Bu)_2_(tet^CF3^)­(*p*-*t*BuC_5_H_4_N) via salt metathesis.^26,27^

The Cp*_2_Ce-cerocene motif is well established. Already in 1986 the synthesis and structural characterization of Cp*_2_CeCl_2_Li­(OEt_2_)_2_ was reported by Rausch et al.^28^ The crystal structures of the similar complex [Cp*_2_CeCl_2_K­(thf)]_*n*_ and the first alkyl derivative Cp*_2_Ce­[CH­(SiMe_3_)_2_] were reported two years later.^29,30^ Many differently substituted amide complexes of the type Cp*_2_Ce­(NRR′) (*R* = *R*′ = SiMe_3_, SiHMe_2_, *i*Pr; *R* = *i*Pr, *R*′ = C­(CH_3_)=CH_2_, *t*Bu, SiHMe_2_, SiMe_3_, Si*t*BuMe_2_; *R* = *t*Bu, *R*′ = SiMe_3_, Si*t*BuMe_2_) were synthesized by the group of Andersen and ours and investigated regarding their oxidizability and thermal rearrangement reactions.^31,32^ Yet the oxidation of Cp*_2_Ce­[N­(SiHMe_2_)_2_] did not result in the formation of a Ce­(IV) complex.

## Results and Discussion

### Cerous Pyrazolates

Treatment of [Cp*_2_CeCl_2_K­(thf)]_*n*_ with one equivalent of Kpz^Me,Me^ in *n*-hexane, toluene or THF and subsequent crystallization from *n*-hexane produced yellow crystals of Cp*_2_Ce­(pz^Me,Me^)­(thf) (**1** ^**thf**^) (Scheme 1). Complex **1** ^**thf**^ is also available via protonolysis of Cp*_2_Ce­[N­(SiHMe_2_)_2_] or Cp*_2_Ce­[N­(SiMe_3_)_2_] with Hpz^Me,Me^ in THF. Performing the protonolysis in a nondonating solvent results in the formation of Cp*_2_Ce­(pz^Me,Me^) (**1**). The ^1^H NMR spectrum in C_6_D_6_ shows two signals for the pz^Me,Me^ ligand at 7.88 and −3.76 ppm in the ratio 1:6, and one signal for the Cp* ligand at 1.97 ppm (Figure S17).

**Scheme 1:**
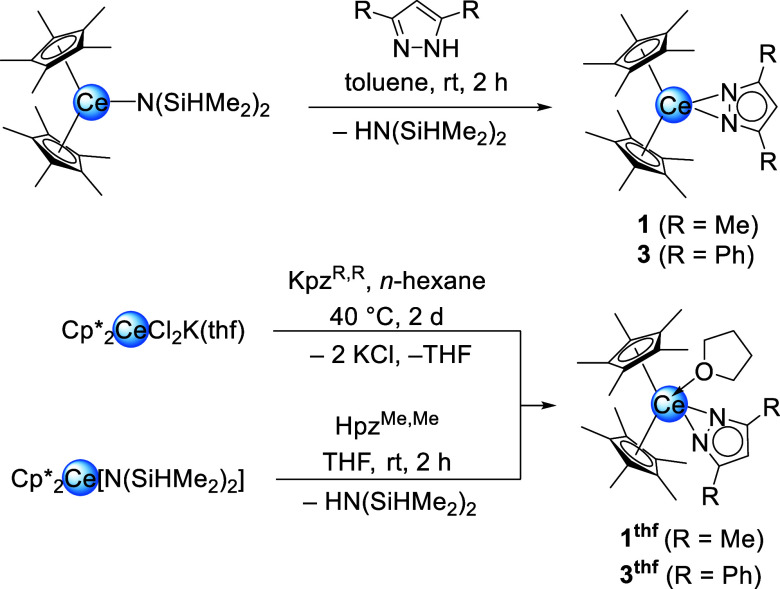
Synthesis of Complexes 1, 1^thf^, 3 and 3^thf^ via Protonolysis and Salt Metathesis from Cp*_2_Ce­[N­(SiHMe_2_)_2_] and [Cp*_2_CeCl_2_K­(thf)]_*n*_, Respectively, Employing Different Pyrazoles and Potassium Pyrazolates

The solid-state structure of **1** is shown in Figure 1/left. The Ce–Ct distance of 2.492 Å (Ct = ring centroid of Cp*) is similar to the known pyrrolate complex Cp*_2_Ce­(NC_4_Me_4_) (2.490–2.492 Å),^33^ but shorter compared to most other Cp*_2_Ce amide derivatives, e. g. Cp*_2_Ce­(N*i*Pr_2_) (2.544–2.562 Å)^31^ or Cp*_2_Ce­(hpp) (hpp = 1,3,4,6,7,8-hexahydro-2*H*-pyrimido­[1,2-*a*]-pyrimidinato) (2.513–2.524 Å).^34^ The κ^2^ bonding of the pz^Me,Me^ ligand results in a larger distance between the cerium and the nitrogen atoms at 2.4334(8) Å than in the κ^1^ coordinated nitrogen atoms in Cp*_2_Ce­(NC_4_Me_4_) (2.400(2) Å) and Cp*_2_Ce­(N*i*Pr_2_) (2.303(4)/2.307(4) Å). The triazolato ligand of the lanthanum sandwich complex Cp*_2_La­(N_3_C­(*t*Bu)­C­(SiMe_3_))­(NC*t*Bu) shows a similar bonding motif, but displays longer Ln–N distances of 2.4719(16) Å and 2.5505(16) Å, respectively.^19^

**Figure 1:**
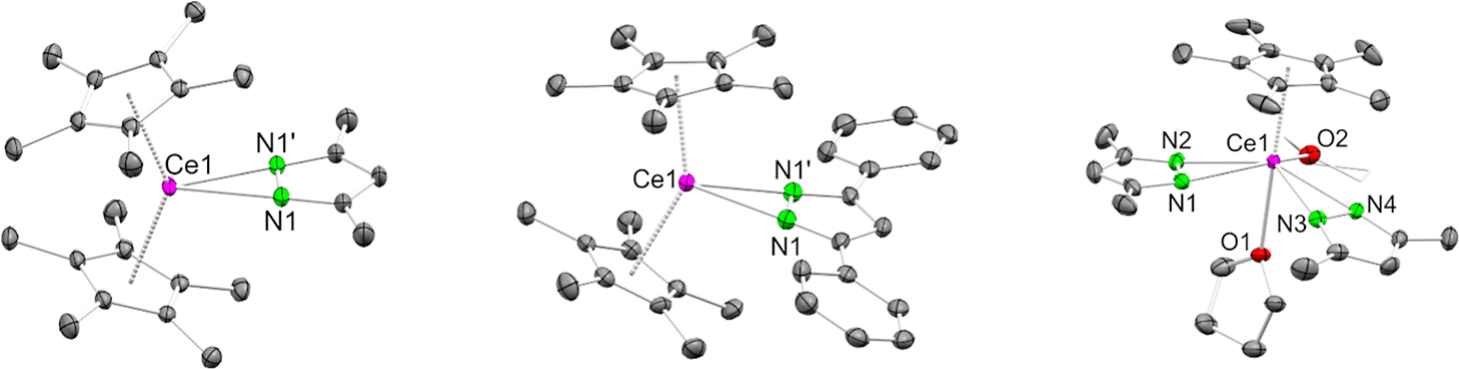
Crystal structures of Cp*_2_Ce­(pz^Me,Me^) (**1**, left), Cp*_2_Ce­(pz^Ph,Ph^) (**3**, middle) and Cp*Ce­(pz^Me,Me^)_2_(thf)_2_ (**5**, right). Ellipsoids are shown at a 50% probability level. A part of **5** is represented by a wireframe model. Hydrogen atoms are omitted for clarity. Selected interatomic distances/angles are listed in the Supporting Information.

Exposing a C_6_D_6_ solution of **1** ^**thf**^ to 1 bar CO_2_ atmosphere resulted in the precipitation of a yellow solid. Crystallization from a THF solution at −40 °C yielded yellow crystals of the dimeric complex [Cp*_2_Ce­(pz^Me,Me^·CO_2_)]_2_ (**2**) (Scheme 2, Figure 2). The ^1^H NMR spectrum shows more signals than the four expected signals which could not be assigned (Figure S25). This insertion behavior had been also observed for the homoleptic cerium pyrazolates, giving access to complexes Ce^III^_4_(pz^Me,Me^·CO_2_)_12_ and Ce^IV^(pz^Me,Me^·CO_2_)_4_.^11^ In complex **2**, two Cp*_2_Ce entities are bridged by two carbamato pz^Me,Me^·CO_2_ ligands via one nitrogen and both oxygen atoms. The cerium centers and the two CO_2_ form an eight-membered ring whose structure resembles a chair conformation.

**Scheme 2:**
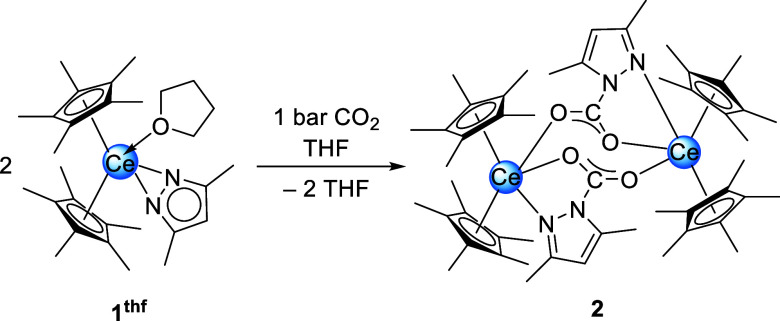
Reaction of Cp*_2_Ce­(pz^Me,Me^)­(thf) (1^thf^) with CO_2_ in THF

**Figure 2:**
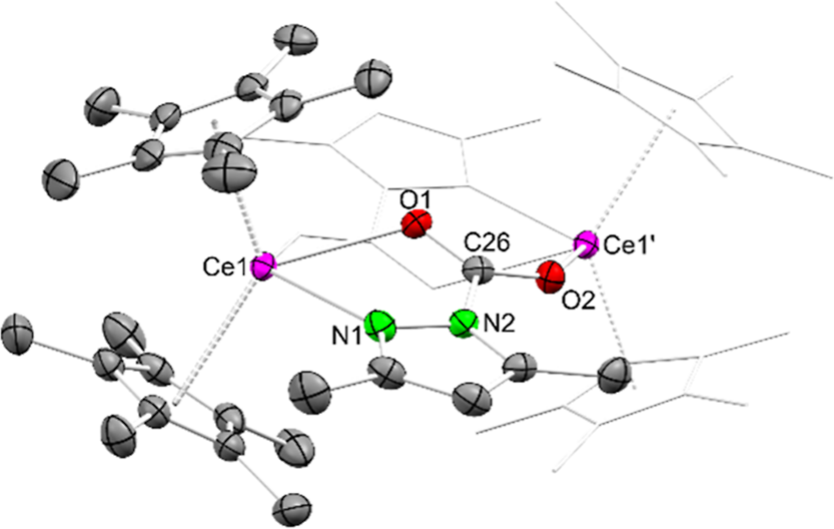
Crystal structure of [Cp*_2_Ce­(pz^Me,Me^·CO_2_)]_2_ (**2**). Ellipsoids are shown at a 50% probability level. Except for the cerium atom the second half of the molecule is represented by a wireframe model. Hydrogen atoms and cocrystallized C_6_D_6_ are omitted for clarity. Selected interatomic distances/angles are listed in the Supporting Information.

Compared to **1** the Ce–Ct distances of 2.540 Å and 2.573 Å are elongated, but only slightly longer than in **1** ^**thf**^. The Ce–N1 distance in **2** amounts to 2.708(3) Å, which suggests a Ce–N donor interaction. The Ce–O distances of 2.469(3) Å and 2.532(2) Å lie within the range of those detected for the cerium­(III) pyrazolate [Ce_4_(pz^Me,Me^·CO_2_)_12_] (2.364(6)–2.771(6) Å).^11^ Similar to the bridging pz^Me,Me^·CO_2_ moieties in [Ce_4_(pz^Me,Me^·CO_2_)_12_], the C–O distances in **2** are in the same range (1.242(4) Å and 1.247(4) Å) indicating delocalization of the C–O double bond. For further comparison, the first structural authentication of a CO_2_-inserted organorare-earth-metal complex was reported for scandocene Cp*_2_Sc­(O_2_C)­C_6_H_4_CH_3_.^35^ Treatment of the cerocene hydride [Cp*_2_Ce­(μ-H)]_2_ with CO_2_ afforded the carbonate species Cp*_2_Ce­(μ-CO_3_)­CeCp*_2_ (ref 36) while Cp*_3_Sm was converted to carboxylate Cp*_2_Sm­(O_2_CC_5_Me_5_).^37^

Stirring a *n*-pentane solution of **1** ^**thf**^ under 1 bar CO_2_ atmosphere resulted first in a color change from green to purple followed by precipitation of yellow **2**. It can be hypothesized that the monomeric complex Cp*_2_Ce­(pz^Me,Me^·CO_2_)­(thf) is formed initially which then loses THF and dimerizes to **2** accompanied by precipitation. The DRIFT spectrum of **2** shows strong absorption bands in the region between 1600 and 1750 cm^–1^ and 1250 and 1400 cm^–1^ for the C–O stretching vibrations (Figure 3, Figure S70).

**Figure 3:**
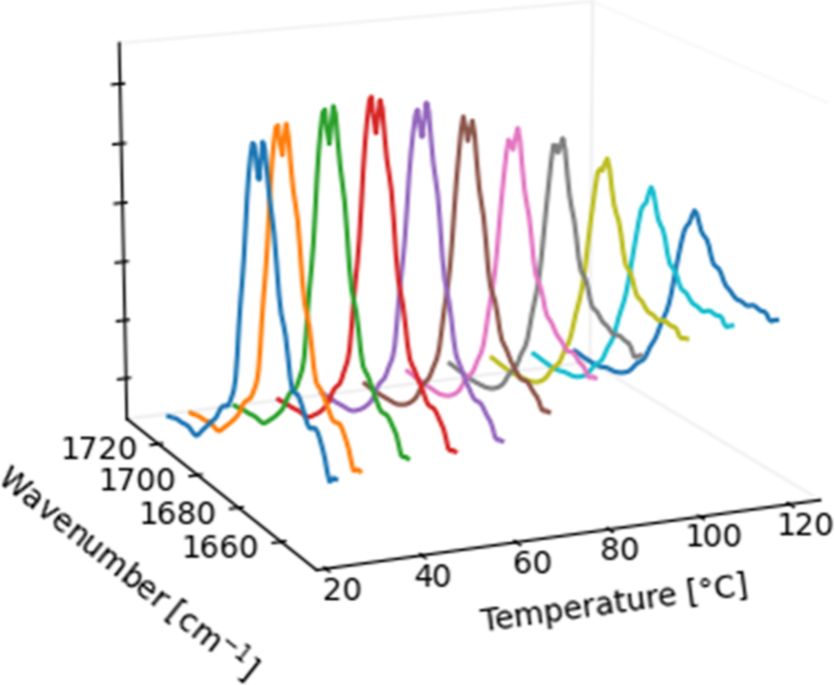
VT DRIFTS of **2** in the range of ṽ = 1730–1650 cm^–1^.

A variable-temperature (VT) NMR study of **2** in THF-*d*_8_ was carried out to determine the reversibility of the CO_2_ insertion (cf. Figure S50). Already at 40 °C distinct signals of the starting material were visible which increased with further heating. Back-cooling to ambient temperature fully reformed complex **2**. Heating the THF-*d*_8_ solution under argon atmosphere at 60 °C for 3 h and measuring a ^1^H NMR spectrum at ambient temperature still gave an unchanged spectrum. However, stirring the THF-*d*_8_ solution of **2** inside the glovebox in an open vial at 40 °C for 30 min yielded **1** ^**thf**^, although some decomposition was observed. Retreatment of **1** ^**thf**^ with CO_2_ restores **2** (cf. Figure S51). After three cycles, most of the starting material had decomposed. Furthermore, exposing **2** to reduced pressure did not yield complex **1**. Performing a thermogravimetric analysis (TGA) showed a 4% mass loss between 60 and 160 °C and another mass loss of 4% between 160 and 200 °C which would be in accordance with the loss of one molecule CO_2_ (4%) each (Figure S81). This is further corroborated by conducting a VT-IR experiment. Upon replacing the argon atmosphere with CO_2_, instant formation of **2** was observed. After renewing the argon atmosphere, the sample was heated in 10 °C increments from 30 to 120 °C (Figure 3). The absorption of the band at 1691 cm^–1^ decreases visibly in the measured temperature range. It appears that the THF donor plays a crucial role in lowering the deinsertion temperature to 40 °C. This behavior resembles the reported complexes [Cp*_2_Sm­(μ-EPh)]_2_ (E = S, Se) which react with CO_2_ to form [Cp*_2_Sm­(μ-CO_2_EPh)]_2_.^38^ Adding THF to [Cp*_2_Sm­(μ-CO_2_SePh)]_2_ leads to decarboxylation and the THF adduct Cp*_2_Sm­(SePh)­(thf).

To probe the steric demand of the pyrazolato ligand, the synthesis of the diphenylpyrazolate complex was envisaged. Performing a salt metathesis of [Cp*_2_CeCl_2_K­(thf)]_*n*_ and Kpz^Ph,Ph^ in *n*-hexane at 40 °C and subsequent crystallization yielded yellow and blue crystals. Similar to **1**, the yellow crystals were identified as the THF adduct Cp*_2_Ce­(pz^Ph,Ph^)­(thf) (**3** ^**thf**^) and the blue ones as THF-free Cp*_2_Ce­(pz^Ph,Ph^) (**3**). By applying high vacuum (10^–4^ mbar) and 100 °C, **3** ^**thf**^ could be converted to **3**. The ^1^H NMR spectrum of **3** in C_6_D_6_ shows the expected signals. Complex **3** crystallizes in the tetragonal space group *P*4_1_2_1_2 (Figure 1/middle). The Ce–Ct distance of 2.486 Å is slightly shorter than in **1**, while the Ce–N distance of 2.444(3) Å is in the same range. The previously reported isostructural ytterbium congener Cp*_2_Yb­(pz^Ph,Ph^) exhibits, due to the significantly smaller rare-earth metal center, shorter Yb–Ct and Yb–N distances of 2.304 Å and of 2.248(18) Å, respectively.^39^

Treatment of **3** with CO_2_ (1 bar pressure) in toluene-*d*_8_ led to a color change from blue to reddish. Addition of *n*-pentane and cooling to −40 °C produced a few red crystals. A single-crystal X-ray diffraction (SCXRD) analysis revealed the formation of the bimetallic insertion product Cp*_2_Ce­(μ-pz^Ph,Ph^·CO_2_)­CeCp*_2_(pz^Ph,Ph^) (**4**) (Scheme 3, Figure 4). Similar to **2** the ^1^H NMR spectra in C_6_D_6_, toluene-*d*_8_ or THF-*d*_8_ were not conclusive (Figures S32–S34). In THF-*d*_8_ only partial insertion was observed at ambient temperature.

**Scheme 3:**
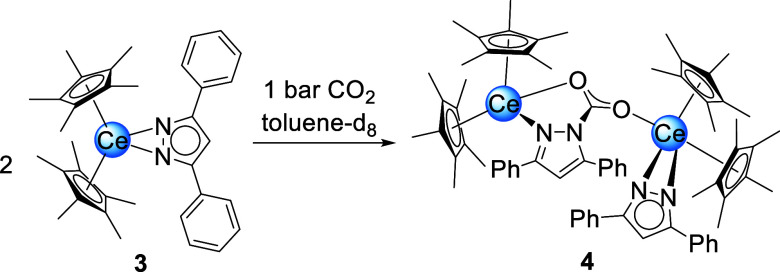
Reaction of Cp*_2_Ce­(pz^Ph,Ph^) (3) and Excess CO_2_ to Form Mono-Inserted Bimetallic 4

**Figure 4:**
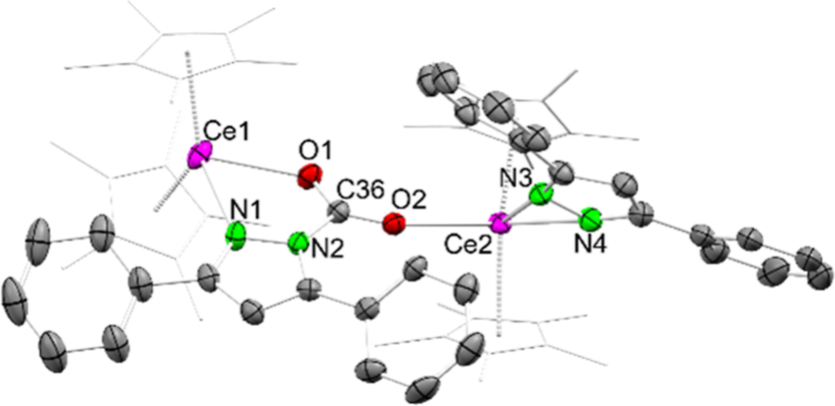
Crystal structure of Cp*_2_Ce­(μ-pz^Ph,Ph^·CO_2_)­CeCp*_2_(pz^Ph,Ph^) (**4**). Ellipsoids are set a 50% probability level. The Cp* ligands are represented by a wireframe model. Hydrogen atoms are omitted for clarity. Selected interatomic distances/angles are listed in the Supporting Information.

The bridging pyrazolato derivative shows a μ-carbamato-κ^2^ *N*,*O*/κ*O*′ binding motif. Noteworthily, both pyrazolato ligands are located on the same side of the complex. The average Ce–Ct distance amounts to 2.535 Å. Both the terminal and the bridging nitrogen atoms exhibit larger distances to the cerium metal centers compared to **3** (2.498(3)–2.563(3) Å). Compared to **2** the C–O distances of the inserted CO_2_ differ far more (1.230(4) Å and 1.254(4) Å) which indicates less delocalization. This is also reflected in increasingly distinct Ce–O distances of 2.429(2) Å and 2.588(2) Å.

Unlike **1**, insertion of CO_2_ occurred into only one pyrazolato ligand of **3**, probably due to the higher steric demand of the phenyl groups compared to the methyl groups. Contrary to **2**, which starts to release CO_2_ at 60 °C in the solid state, **4** is stable under CO_2_ atmosphere but partially releases CO_2_ under argon atmosphere at ambient temperature. This hampered the isolation of a sufficient quantity of complex **4** for FTIR and TGA measurements.

Similarly to **1**, Cp*CeI_2_(thf)_3_ was treated with two equivalents of Kpz^Me,Me^ in *n*-hexane aiming at the corresponding half-sandwich complex (Scheme 4). Slow evaporation at ambient temperature yielded colorless crystals of Cp*Ce­(pz^Me,Me^)_2_(thf)_2_ (**5**, Figure 1/right). The ^1^H NMR spectrum in C_6_D_6_ shows five signals seemingly assignable to **5** (Figure S35), however, slow ligand scrambling is indicated by the appearance of peaks ascribed to **1** ^**thf**^. The ligands in **5** are arranged in a distorted trigonal-byramidal fashion. The Cp* and one THF ligand occupy the axial positions while the remaining THF as well as the two pyrazolatos bend away from the Cp* in the equatorial positions. Compared to **1** ^**thf**^ the Ce–Ct distance of **5** is shorter (2.505 Å vs 2.535 Å). The Ce–N distances involving the κ^2^-bonded pyrazolato ligands range from 2.432(2) Å to 2.500(3) Å. The equatorial THF is closer (Ce–O 2.591(3) Å) to the metal center than the axial one (Ce–O 2.6195(18) Å).

**Scheme 4:**
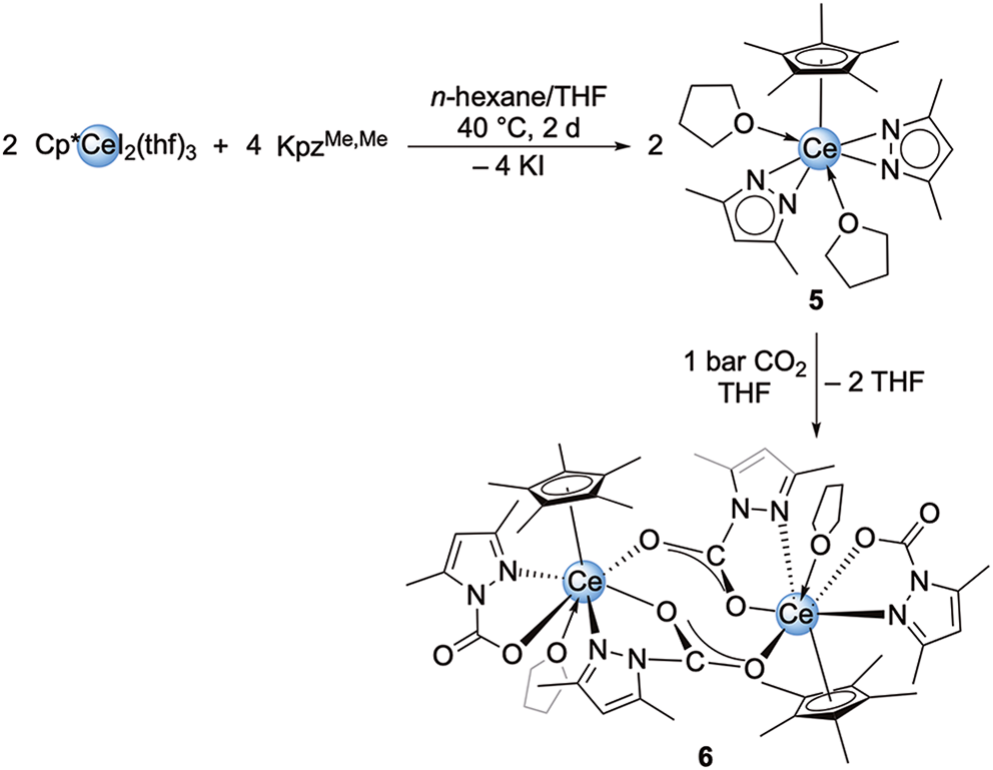
Salt-Metathesis Reaction of Cp*CeI_2_(thf)_3_ and Kpz^Me,Me^ to Yield Cp*Ce­(pz^Me,Me^)_2_(thf)_2_ (**5**)­^a^

a Treatment of **5** with CO_2_ resulted in the formation of dimeric [Cp*Ce­(pz^Me,Me^·CO_2_)_2_(thf)]_2_ (**6**).

Overall, the coordination geometry of **5** is distinct from the fluorenyl half-sandwich complex FluCe­(pz^Me,Me^)_2_(thf)_2_ which adopts a slightly bent pseudo square pyramidal coordination geometry with trans-positioned κ^2^-pyrazolato ligands.^40^ The Ce–N distances in FluCe­(pz^Me,Me^)_2_(thf)_2_ average 2.480 Å.

Exposing a solution of **5** in C_6_D_6_ to 1 bar of CO_2_ led to the precipitation of a yellow powder. Crystallization from a THF solution at −40 °C yielded yellow crystals of the dimeric complex [Cp*Ce­(pz^Me,Me^·CO_2_)­(μ-pz^Me,Me^·CO_2_)­(thf)]_2_ (**6**) (Scheme 4, Figure 5). The ^1^H NMR spectrum of **6** features several broad peaks which could not be assigned (Figure S37).

**Figure 5:**
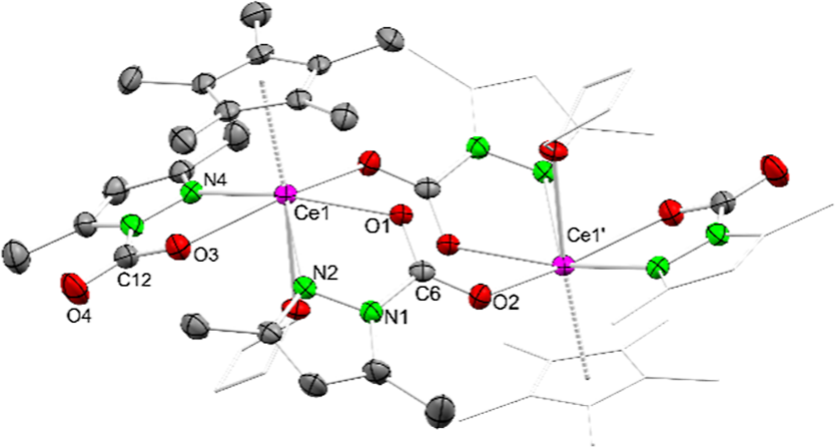
Crystal structure of [Cp*Ce­(pz^Me,Me^·CO_2_)_2_(thf)]_2_ (**6**). Ellipsoids are set a 50% probability level. Part of the molecule is represented by a wireframe model for better visibility. Hydrogen atoms are omitted for clarity. Selected interatomic distances/angles are listed in the Supporting Information.

The cerium centers in **6** are coordinated axially by one Cp* ligand and one THF donor and equatorially by one terminal pz^Me,Me^·CO_2_ carbamato ligand. Furthermore, two pz^Me,Me^·CO_2_ ligands bridge the two cerium centers, in a similar manner as observed for **2**. The Ce–Ct distance is 2.522 Å and thus elongated compared to precursor **5**. The Ce1–N2/N4 distances amount to 2.743(4) Å and 2.727(4) Å and are typical for a metal–donor interaction. The O–C–O distances of the terminal pz^Me,Me^·CO_2_ ligands imply a localized double bond (C12–O3 1.263(6) Å, C12–O4 1.222(5) Å) and delocalization for the bridging ligands (C6–O1 1.246(5) Å, C6–O2 1.250(5) Å), fully in line with the Ce–O distances which are shorter for the terminal pz^Me,Me^·CO_2_ ligand (Ce1–O3 2.389(3) Å) compared to the bridging ones (Ce1–O1 2.479(3) Å, Ce1̀'–O2 2.564(3) Å). This is further supported by DRIFTS measurements. The IR spectrum of **6** shows strong absorption bands at 1728 and 1668 cm^–1^ ascribed to the stretching vibration of the C–O double bond. In the area between 1470 and 1290 cm^–1^ there are several strong bands for the stretching of the C–O single bond as well as the delocalized C–O bonds. The TGA of complex **6** revealed no clear loss of CO_2_. Instead, a mass loss of 60% was observed in the range from 85 to 480 °C, showing two steps of 45% between 85 and 300 °C and 15% between 300 and 480 °C (Figure S82).

### Cerous Triazolates and Tetrazolates

To examine the effect of less nucleophilic azolato ligands, the synthesis of sandwich cerium triazolates and tetrazolates was pursued. However, both the salt metathesis of [Cp*_2_CeCl_2_K­(thf)]_*n*_ and potassium triazolate Ktz^Me,Me^ as well as the protonolysis of Cp*_2_Ce­[N­(SiHMe_2_)_2_] with triazole Htz^Me,Me^ yielded only slightly soluble yellow solids. This can be interpreted by the formation of oligomeric structures. For example, Zhang et al. reported that the protonolysis of Cp′_3_Yb (Cp′ = C_5_H_4_Me) with Htz^H,H^ in THF yielded tetrameric [Cp′_2_Yb­(tz^H,H^)]_4_.^41^ We found that addition of DMAP to the protonolysis reaction mixture and subsequent crystallization from Et_2_O gave colorless needles of Cp*_2_Ce­(tz^Me,Me^)­(dmap) (**7**) (Scheme 5, Figure 6/left). The ^1^H NMR spectrum in C_6_D_6_ shows the expected five signals (Figure S38).

**Scheme 5:**
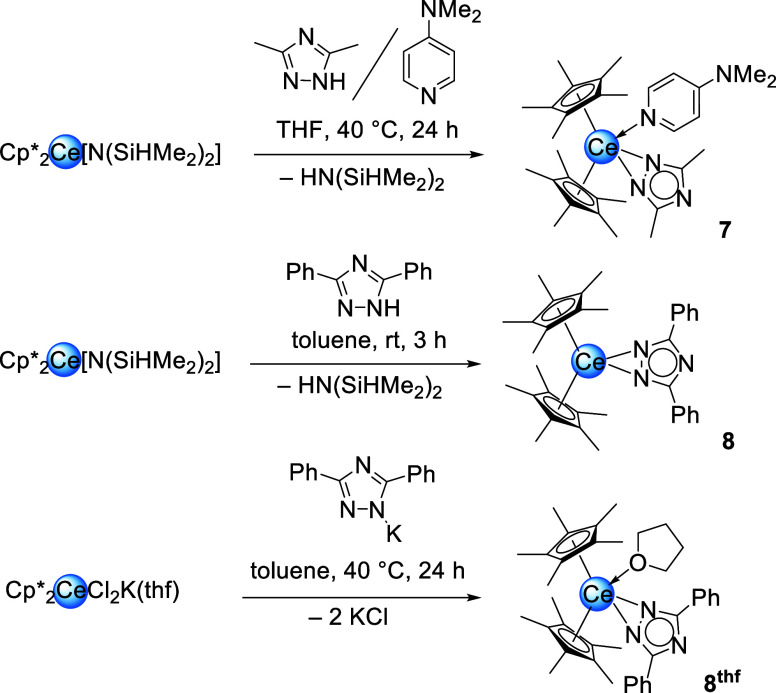
Synthesis of Cp*_2_Ce­(tz^Me,Me^)­(dmap) (**7**), Cp*_2_Ce­(tz^Ph,Ph^) (**8**) and Cp*_2_Ce­(tz^Ph,Ph^)­(thf) (**8** ^thf^) via Protonolysis and Salt Metathesis, Respectively

**Figure 6:**
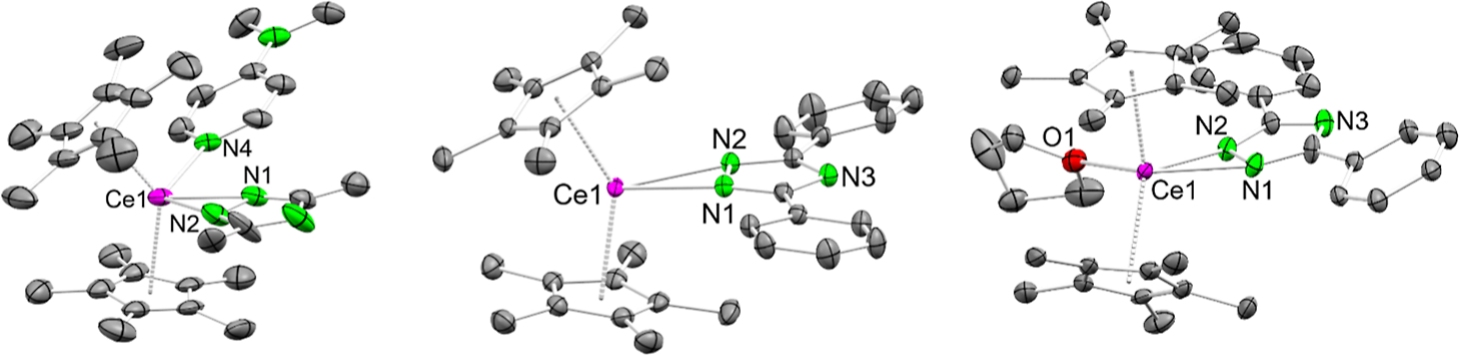
Crystal structures of Cp*_2_Ce­(tz^Me,Me^)­(dmap) (**7**, left), Cp*_2_Ce­(tz^Ph,Ph^) (**8**, middle) and Cp*_2_Ce­(tz^Ph,Ph^)­(thf) (**8** ^**thf**^, right). Complex **7** has two molecules in the asymmetric unit. One molecule is severely disordered and omitted for clarity. Ellipsoids are set at a 50% probability level. Hydrogen atoms are omitted for clarity. Selected interatomic distances/angles are listed in the Supporting Information.

The Ce–N distances of **7** involving the triazolato ligand amount to 2.458(4) Å and 2.537(4) Å and are similar to the C–N­(pyrazolato) distances in **1** ^**thf**^. The DMAP ligand coordinates with the pyridine nitrogen atom at a distance of 2.592(4) Å. Contrary to the pyrazolates, complex **7** does not react with CO_2_ at ambient temperature. This could be due to the presence of the stronger donor ligand DMAP compared to THF, the less nucleophilic triazolato ligand or both.

Analogously to the pyrazolates the synthesis of the diphenyl triazolate derivative was carried out next. Once more, both the donor-free Cp*_2_Ce­(tz^Ph,Ph^) (**8**) as well as the THF-containing sandwich complex Cp*_2_Ce­(tz^Ph,Ph^)­(thf) (**8** ^**thf**^) could be isolated via protonolysis or salt metathesis, individually (Scheme 5, Figure 6/middle, right).

The ^1^H NMR spectra in C_6_D_6_ revealed four and six signals, respectively (Figures S40 and S42). For **8**, the signal of the Cp* methyl protons appeared at 2.22 ppm, the signals for the *para* and *meta* protons at 4.64 and 3.05 ppm. The *ortho* protons are detected at −5.08 ppm. Compared to **8**, the signals of **8** ^**thf**^ are shifted to lower field, except the two THF signals which appear at −4.31 and −13.63 ppm.

There are two independent units in the crystal structure of **8**. The triazolato ligands each coordinate in κ^2^-fashion to the cerium center. The Ce–N distances range from 2.4611(15) Å to 2.4947(16) Å being longer than in the corresponding pyrazolate **3**. The average Ce–Ct distance of 2.473 Å is slightly shorter than in **3**. This can be attributed to the lower nucleophilicity of the triazolato compared to the pyrazolato ligand. Adding CO_2_ to **8** in C_6_D_6_ or *n*-pentane resulted in a color change from blue to dark green and precipitation of an off-white solid. The solid shows an IR absorption band around 1677 cm^–1^ ascribed to the stretching vibration of a C–O double bond. Numerous strong bands are detected between 1467 cm^–1^ and 1355 cm^–1^ corresponding to the stretching of C–O single bonds and delocalized C–O bonds. This suggests a successful reaction of **8** with CO_2_, however, in THF-*d*_8_ no CO_2_ insertion was detected via NMR spectroscopy. Due to the low solubility in aromatic solvents and the rapid deinsertion in THF, no crystalline material could be obtained to date.

Turning to the even less nucleophilic tetrazolates, the sandwich complex [Cp*_2_Ce­(tet^Ph^)]_3_ (**9-Ce**) could be obtained via salt metathesis from [Cp*_2_CeCl_2_K­(thf)]_*n*_ and Ktet^Ph^ (Scheme 6). The ^1^H NMR spectrum shows only three signals due to overlap of the *meta* protons of the phenyl ring with Cp* methyls (Figure S44). Contrary to the pyrazolates and triazolates **9-Ce** constitutes a trimeric structure (Figure 7). Two Cp* ligands coordinate to each metal center while the tetrazolato ligands bridge the cerium atoms via a μ-κ^2^ *N*,*N*′/κ^2^ *N*″,*N*‴ binding motif.

**Scheme 6:**
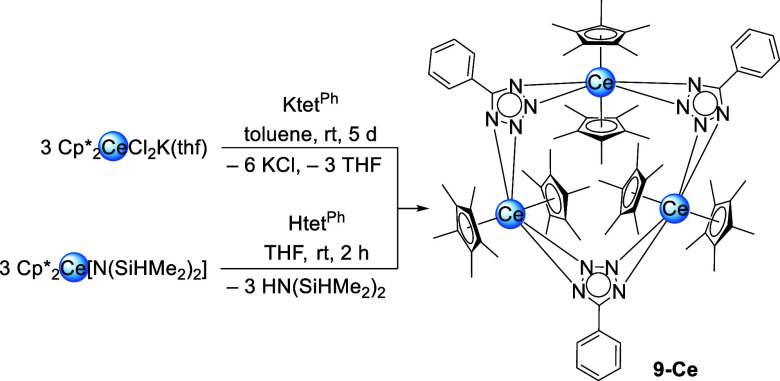
Synthesis of [Cp*_2_Ce­(tet^Ph^)]_3_ (**9-Ce**) via Salt Metathesis and Protonolysis

**Figure 7:**
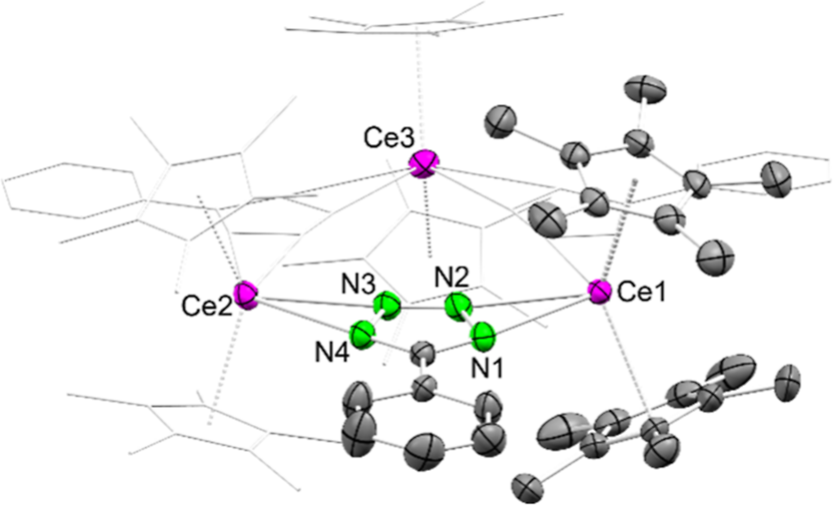
Crystal structure of [Cp*_2_Ce­(tet^Ph^)]_3_ (**9-Ce**). Ellipsoids are set at a 50% probability level. Part of the molecule is represented by a wireframe model for better visibility. The lanthanum congener **9-La** is isostructural. Hydrogen atoms are omitted for clarity. Selected interatomic distances/angles are listed in the Supporting Information.

The cerium centers and the phenyltetrazolato ligands form a slightly distorted plane. The cerium nitrogen distances range from 2.596(3) Å to 2.682(3) Å for the inner (N2/N3, N6/N7, N10/N11) and from 2.620(3) Å to 2.830(4) Å for the outer nitrogen atoms (N1/N4, N5/N8, N9/N12). Complex **9-Ce** does not insert CO_2_ at 1 bar pressure in C_6_D_6_, toluene-*d*_8_ or THF-*d*_8_. Despite the larger size of the La­(III) center, no CO_2_ insertion was observed for the corresponding [Cp*_2_La­(tet^Ph^)]_3_ (**9-La**). Isostructural complex **9-La** was synthesized according to the salt metathesis shown in Scheme 6.

We also probed the protonolysis of the pyrazolato ligand of **1** ^**thf**^ with the more acidic Htz^Me,Me^ in THF-*d*_8_ and Htet^Ph^ in toluene.^42^ However, instead of the azolate sandwich complexes no product could be isolated. Among other compounds the ^1^H NMR spectra show the formation of HCp* (Figures S52 and S53).

### Electronic Absorption Spectra

The pyrazolate complexes **1** and **1** ^**thf**^ display similar absorption spectra (in *n*-hexane), featuring a slight shoulder at around 268 nm and growing absorption until the cutoff wavelength of the solvent (Figure S83). The half-sandwich congener **5** exhibits a shoulder at 275 nm but has a similar increase of the absorption toward the cutoff. The global absorption maximum for the diphenyl derivative **3** is detected at 257 nm, while the tz^Ph,Ph^ complex **8** displays two local maxima at 262 and 235 nm. The global absorption maximum of trimetallic **9-Ce** appears at 242 nm. The complexes **1** ^**thf**^, **2**, **3** ^**thf**^, **6**, **7**, and **8** ^**thf**^ were measured in THF solution (Figure S84). The CO_2_-inserted complex **2** exhibits two absorption maxima at 266 and 225 nm, while none was observed for the half-sandwich CO_2_ complex **6** in the absorption range from 300 to 220 nm. A local maximum of **7** is detected at 254 nm. Overall, the THF adducts show similar spectra to their donor-free congeners. Due to poor solubility in *n*-hexane and partial deinsertion of CO_2_ in THF and CH_3_CN complex **4** was measured in toluene (Figure S91), ruling out a measurement at shorter wavelengths. The absorption ranges from about 400 to 300 nm.

### Electrochemical Properties

The redox behavior of the bis­(alkoxy) cerium­(IV) metallocene complexes Cp*_2_Ce­(OR)_2_ (R = Et, *i*Pr, *t*Bu, CH_2_ *t*Bu, SiMe_3_, SiPh_3_) was recently investigated by cyclic voltammetry.^43^ Naturally, we probed the electrochemical properties of the compounds under study to determine the accessibility of the Ce­(IV) oxidation state and if sandwich cerium­(IV) pyrazolates are feasible and isolable. The respective electrochemical measurements were performed in a glovebox (argon atmosphere) at ambient temperature with a glassy-carbon (GC) working electrode in THF solvent with 0.1 M [*n*Pr_4_N]­[B­(C_6_H_3_(CF_3_)_2_-3,5)_4_] supporting electrolyte.

All potentials are referenced versus the Fc/Fc^+^ redox couple. Without exception, the surveyed complexes revealed qualitatively irreversible oxidation waves (Figures 8 and S98–S107). The isolation of single waves did not result in reversible redox events. The cyclic voltammogram of **1** ^**thf**^ exhibits three oxidation waves at −0.46 V, −0.24 and +0.37 V, respectively. Similar oxidation events are visible for **3** ^**thf**^ at −0.24 V and +0.41 V, with an additional event at +0.74 V. Overall, the cyclic voltammogram is reminiscent of that of the chloride precursor [Cp*_2_CeCl_2_K­(thf)]_*n*_ whose Ce­(III/IV) redox couple at *E*_ox_ = −0.57 V was found irreversible as well.^43^ The waves of the diphenyl-substituted triazolato derivative **8** ^**thf**^ appeared at more negative potentials of −0.29 V, +0.36 V and +0.60 V indicating an easier oxidation compared to the corresponding pyrazolate complex. The trimeric complex **9-Ce** exhibits redox events at −0.16 V, +0.41 V and +0.70 V.

**Figure 8:**
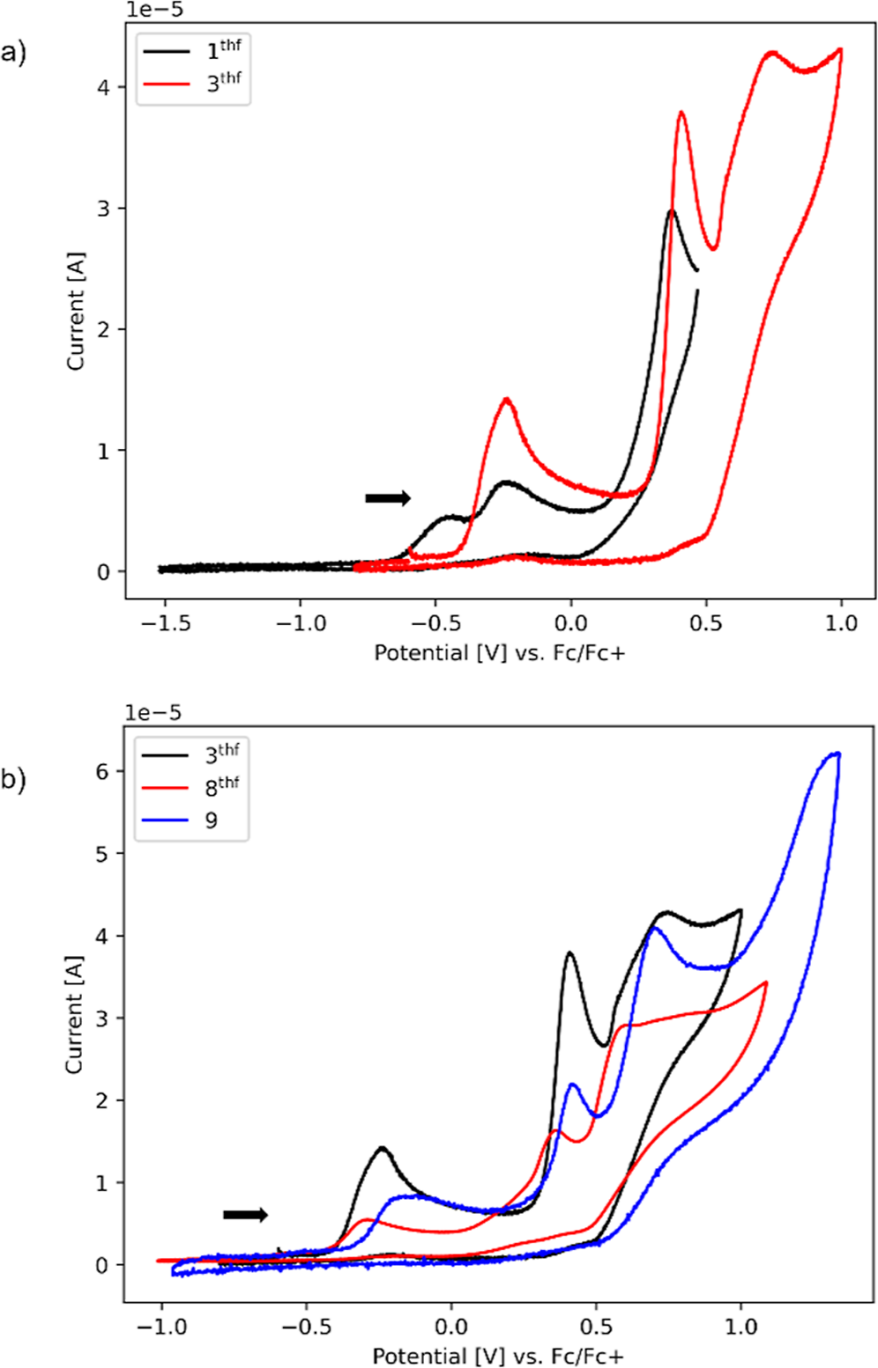
(a) Cyclic voltammograms of Cp*_2_Ce­(pz^Me,Me^)­(thf) (**1** ^**thf**^, black) and Cp*_2_Ce­(pz^Ph,Ph^)­(thf) (**3** ^**thf**^, red) vs Fc/Fc^+^ in THF at a glassy-carbon electrode obtained at a scan rate of 50 mV/s. The arrow indicates the scan direction. The analyte concentration was 1 mM, and the electrolyte concentration was 100 mM [*n*Pr_4_N]­[B­(C_6_H_3_(CF_3_)_2_-3,5)_4_]. (b) Cyclic voltammograms of Cp*_2_Ce­(pz^Ph,Ph^)­(thf) (**3** ^**thf**^, black), Cp*_2_Ce­(tz^Ph,Ph^)­(thf) (**8** ^**thf**^, red) and [Cp*_2_Ce­(tet^Ph^)]_3_ (**9-Ce**, blue) vs Fc/Fc^+^ in THF at a glassy-carbon electrode. The arrow indicates the scan direction. The analyte concentration was 1 mM, and the electrolyte concentration was 100 mM [*n*Pr_4_N]­[B­(C_6_H_3_(CF_3_)_2_-3,5)_4_]. The scan rate was 50 mV/s for **3** ^**thf**^ and **8** ^**thf**^ and 250 mV/s for **9-Ce**.

The corresponding lanthanum complex Cp*_2_La­(pz^Me,Me^)­(thf) (**1** ^**thf**^ **-La**) was synthesized to compare the electrochemical behavior of **1** ^**thf**^ with a redox-innocent metal (Figures 9 and S108). The lanthanum derivative features oxidation events at 0.00 V, +0.30 V, +0.51 V and +0.70 V. Compared to the cerium complex **1** ^**thf**^ the oxidation events are shifted to a higher potential and suggest that the first oxidation event detected for **1** ^**thf**^ at *E*_ox_ = −0.46 V belong to the Ce­(III/IV) redox couple. At higher potential, the cyclic voltammogram looks similar to those of the cerium congeners, hinting at ligand-based oxidation processes. The attempted chemical oxidation of **1** ^**thf**^ with C_2_Cl_6_ in C_6_D_6_ did not result in the formation of a Ce­(IV) complex (Scheme 7, Figure S55). The corresponding ^1^H NMR spectrum of the blue solution revealed signals assigned to Cp*_2_, alongside with a paramagnetic Ce­(III) species. A crystallization attempt from *n*-hexane resulted in a few colorless crystals which could be identified as the Ce­(III) cluster Cp*_4_Ce_4_(μ_2_-Cl)_3_(μ_3_-Cl)_2_(μ_4_-Cl)­(μ_2_-pz^Me,Me^)­(pz^Me,Me^)­(thf) (**10**). Complex **10** features a Ce_4_Cl_6_-cluster core. Each cerium center is capped by one Cp* ligand. One pyrazolato ligand coordinates κ_^2^_ in a terminal fashion while the other bridges two metal centers in a μ-1κ^2^(*N*,*N*′):2κ­(*N*) fashion. The Cp*_4_Ln_4_Cl_6_ motif resembles the structures of the reported rare-earth-metal complexes Cp*_5_Nd_5_(AlMe_4_)­Cl_9_ (ref 44) and Cp*_6_Sm_5_Cl_9_.^45^ The Ce–Cl distances range from 2.7529(16) to 3.1121(15) Å. For comparison, the Ce–Cl distances in the dodecanuclear cluster [(C_5_H_4_SiMe_3_)­CeCl_2_]_12_ are between 2.845(2) and 3.164(3) Å.^46^ Apparently, any transient Ce­(IV) decomposes under formation of a Ce­(III) species and Cp*_2_ or the ligand is oxidized directly. This is in contrast to the sandwich alkoxide complexes which can be isolated, albeit slow conversion to Ce­(IV) half-sandwich derivatives is noted.

**Figure 9:**
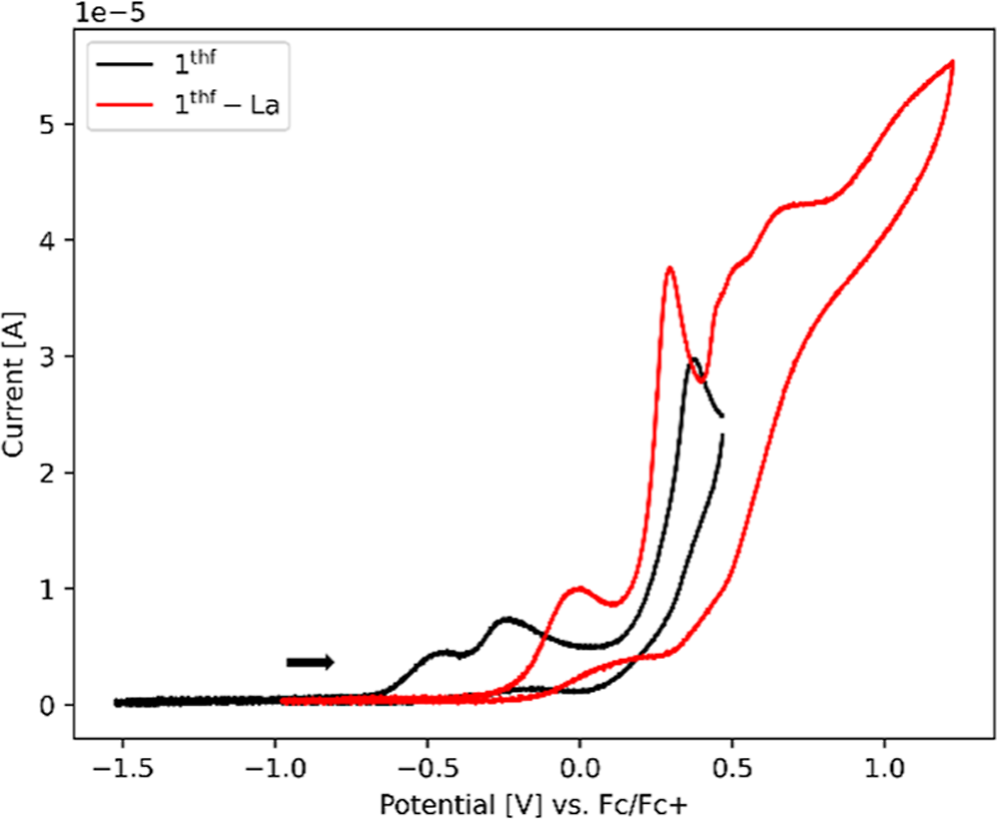
Cyclic voltammograms of Cp*_2_Ce­(pz^Me,Me^)­(thf) (**1** ^**thf**^, black) and Cp*_2_La­(pz^Me,Me^)­(thf) (**1** ^**thf**^ **-La**, red) vs Fc/Fc^+^ in THF at a glassy-carbon electrode obtained at a scan rate of 50 mV/s. The arrow indicates the scan direction. The analyte concentration was 1 mM and the electrolyte concentration was 100 mM [*n*Pr_4_N]­[B­(C_6_H_3_(CF_3_)_2_-3,5)_4_].

**Scheme 7:**
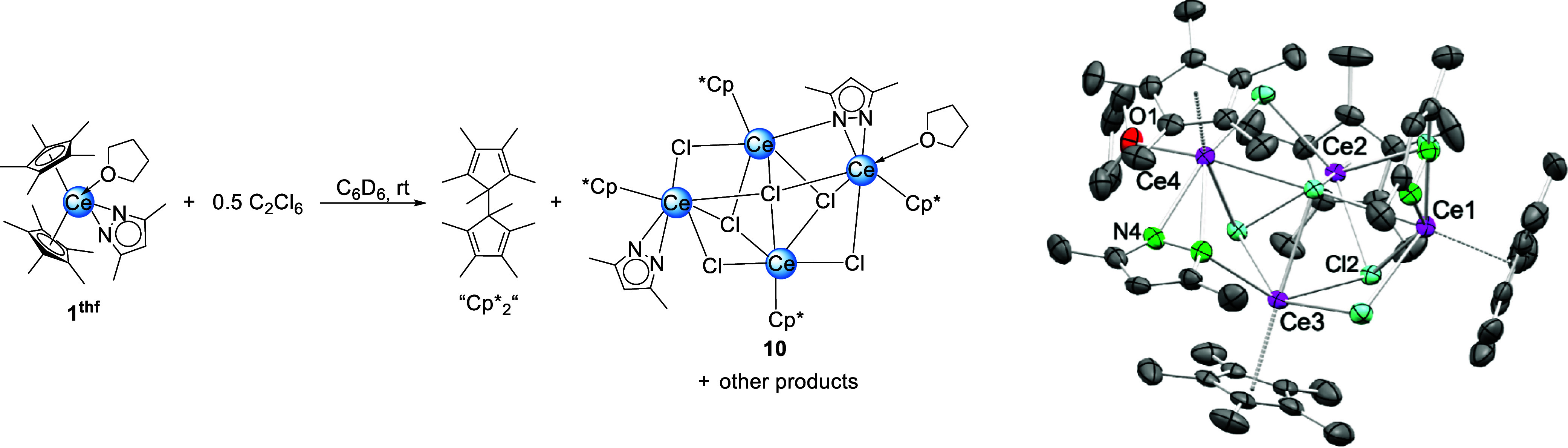
Oxidation of Cp*_2_Ce­(pz^Me,Me^)­(thf) (**1** ^thf^) with C_2_Cl_6_ Yielding Cp*_2_, Cp*_4_Ce_4_Cl_6_(pz^Me,Me^)_2_(thf) (**10**) and Other Products (Left): Crystal Structure of **10** (Right)

For further comparison, the alkoxide complexes Cp*_2_Ce­(OR)_2_ (R = Et, *i*Pr, *t*Bu, CH_2_ *t*Bu) display fully reversible oxidation waves of the Ce­(III/IV) redox couple at *E*_ox_ = −1.5 V.^43^ Conceivably, the pyrazolato ligand is not a strong enough donor to support the Ce­(IV) oxidation state within the metallocene scaffold. The addition of C_2_Cl_6_ to the lanthanum congener **1** ^**thf**^ **-La** in C_6_D_6_ was also investigated. The ^1^H NMR spectrum after 10 min shows mostly starting material **1** ^**thf**^ **-La** and small peaks belonging to Cp*_2_. The integral ratio of the pz-CH proton compared to the six proton signals of Cp*_2_ at 1.15 ppm accounted for 1:0.03 and increased to 1:0.21 after 18 h (Figures S56 and S57). The proton NMR spectrum of the oxidation with **1** ^**thf**^ did not show any signals of the starting material left after 10 min. This hints at a possible Ce­(IV) intermediate followed by decomposition rather than direct ligand oxidation. It is noteworthy that the reaction of KCp* with C_2_Cl_6_ led also to the formation of Cp*_2_ corroborating that Cp*^–^ can be directly oxidized by C_2_Cl_6_.

### Propylene Carbonate Formation

Previous investigations by our group explored the catalytic activity of metal pyrazolates in the cycloaddition of epoxides and CO_2_.^11–14^ Naturally, we wanted to determine the effect of different azolates on the catalytic performance. The reaction was conducted in propylene oxide as the solvent with 0.5 mol% catalyst, 1 mol% TBAB (TBAB = tetra-*n*-butylammonium bromide) as cocatalyst and 1 bar CO_2_ pressure at ambient temperature for 24 h (Table 1). The conversion was determined via ^1^H NMR spectroscopy. The donor-free dimethylpyrazolate **1** showed a slightly better performance than the THF adduct **1** ^**thf**^ (97% versus 95% conversion, entries 1 and 2). The sterically more demanding diphenylpyrazolate **3** displayed a slightly lower conversion of 92% (entry 3). Using the THF adduct **3** ^**thf**^ significantly decreased the carbonate formation to 75% (entry 4). Albeit having two azolate moieties, the half-sandwich congener **5** shows a catalytic activity similar to **3** ^**thf**^ (entry 5), i. e. lower than **1** ^**thf**^. Complex **7** exhibits a significantly lower conversion of 63% compared to **1** ^**thf**^ (entry 6), probably due to the coordination of the stronger DMAP donor. The diphenyltriazolato derivatives **8** and **8** ^**thf**^ gave comparable results indicating a slightly better performance of the THF adduct (entries 7 and 8). Overall, the exchange of the pyrazolato for the triazolato ligand has only minor influence. The best performance with only traces of starting material left displayed the trimeric tetrazolato complex **9-Ce** (entry 9). This is in accordance with the catalytic performance of magnesium pyrazolates which exhibited the highest activity for the fluorinated derivative Mg_2_(pz^CF3,CF3^)_4_(thf)_3_ which like complex **9-Ce** did not form an isolable carbamate complex.^12^

**Table 1:**
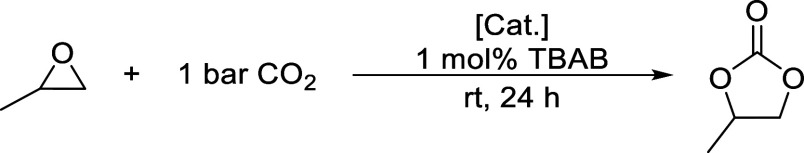
Formation of Propylene Carbonate From Propylene Oxide and CO_2_, Catalyzed by the Sandwich Cerium Azolates Under Study

entry^a^	catalyst	conversion [%]^b^	TON^c^
1	**1**	97	194
2	**1** ^**thf**^	95	190
3	**3**	92	184
4	**3** ^**thf**^	75	150
5	**5**	74	148
6	**7**	63	126
7	**8**	87	174
8	**8** ^**thf**^	92	184
9	**9-Ce**	>99	199

a Reaction conditions: 1 bar CO_2_, 0.5 mol% catalyst or 0.167 mol% for **9-Ce** (≡0.5 mol% Ce centers), 1 mol% TBAB at ambient temperature for 24 h in neat epoxide.

b Determined via ^1^H NMR by comparison of the proton integrals in α-position of the propylene oxide and the propylene carbonate.

c ((1/[Ce])/100)·conversion.

Compared to the systems described in literature the catalysts in this study only exhibit moderate catalytic activity. A heteroscorpionate lanthanum complex by the group of Otero revealed a TOF of 15,000 h^–1^ and a TON value of 306,667.^47^

## Conclusion

Differently substituted sandwich cerium­(III) azolates were synthesized and their reactivity toward CO_2_ examined. The pyrazolate complex Cp*_2_Ce­(pz^Me,Me^) inserts CO_2_ forming dimeric [Cp*_2_Ce­(pz^Me,Me^·CO_2_)]_2_. Extending the steric demand from dimethyl to the diphenylpyrazolato derivative gave a bimetallic complex with only one inserted CO_2_. The half-sandwich Cp*Ce­(pz^Me,Me^)_2_(thf)_2_ exhibits exhaustive CO_2_ insertion and forms a dimer with bridging and terminal pz^Me,Me^·CO_2_ ligands. The triazolate Cp*_2_Ce­(tz^Me,Me^)­(dmap) could only be isolated by utilizing the strong donor DMAP, which however thwarted the insertion of CO_2_. In contrast, the donor-free triazolate Cp*_2_Ce­(tz^Ph,Ph^) gave CO_2_ insertion in nondonating solvents. Overall, the less nucleophilic and less basic triazolato ligands seem to have a lower affinity for CO_2_ insertion than the pyrazolatos. The decreasing carboxophilicity is ultimately revealed by trimeric tetrazolate complex [Cp*_2_Ce­(tet^Ph^)]_3_ which gave no detectable insertion of CO_2_. Electrochemical measurements showed quantitively irreversible oxidation events, which are accompanied by the formation of Cp*_2_ as the possible oxidation product. Cp*_2_ is also produced in chemical oxidations of Cp*_2_Ce­(pz^Me,Me^)­(thf) or KCp* with C_2_Cl_6_. All cerium­(III) compounds promote the cyclization reaction of propylene oxide and CO_2_ to propylene carbonate although only with moderate catalytic activity.

## Experimental Section

### General Procedures

No uncommon hazards are noted. All reactions were performed under an inert atmosphere (Ar) by using a glovebox (MBraun UNIlab pro; <0.1 ppm of O_2_, <0.1 ppm of H_2_O) or according to standard Schlenk techniques in oven-dried glassware. Unless otherwise stated, the solvents were purified with Grubbs type columns (MBraun SPS, solvent purification system) and stored in a glovebox. CO_2_ was purchased from Westfalen AG. Anhydrous cerium­(III) chloride (99.5%) was purchased from ABCR and activated via Soxhlet extraction with THF giving CeCl_3_(thf). C_6_D_6_, toluene-*d*_8_ and THF-*d*_8_ were purchased from Sigma-Aldrich and dried over NaK alloy. C_6_D_6_, toluene-*d*_8_, and THF-*d*_8_ were filtered and stored in a glovebox. THF-*d*_8_ was also stored over molecular sieves (3 Å). 3,5-Dimethyltriazole and 5-phenyltetrazole were purchased from TCI. DMAP, potassium bis­(trimethylsilyl)­amide, 3,5-dimethylpyrazole and C_2_Cl_6_ were purchased from Sigma-Aldrich. Lithium bis­(dimethylsilyl)­amide was synthesized from bis­(dimethylsilyl)­amine (abcr) using *n*BuLi.^48^ 3,5-Diphenyltriazole,^49^ KCp*,^50^ [Cp*_2_CeCl_2_K­(thf)]_*n*_,^29^ Cp*CeI_2_(thf)_3_ (ref 51) and Cp*_2_Ce­[N­(SiHMe_2_)_2_][Bibr ref31] were synthesized according to published procedures. {Cp*_2_LaCl_2_K­(thf)} was prepared in the same way as [Cp*_2_CeCl_2_K­(thf)]_*n*_. Kpz^Me,Me^, Kpz^Ph,Ph^, Ktz^Ph,Ph^, and Ktet^Ph^ were synthesized from the respective azoles with KN­(SiMe_3_)_2_ in toluene.

NMR spectra were recorded on a Bruker AVII+400 (^1^H: 400.11 MHz, ^13^C: 100.61 MHz) or a Bruker AVII+500 (^1^H: 500.13 MHz, ^13^C: 125.76 MHz) at 26 °C using J. Young-valved NMR tubes. ^1^H and ^13^C NMR chemical shifts are referenced to a solvent resonance and reported in parts per million (ppm) relative to tetramethylsilane. Analysis of the NMR spectra was performed with ACD/NMR Processor Academic Edition (Product Version: 12.01). Multiplicities of signals are given as singulet (s), doublet (d), triplet (t) and multiplet (m). DRIFT spectra were recorded on a Bruker INVENIO R spectrometer and converted using the Kubelka–Munk refinement. The samples were mixed with KBr and measured in a cell with KBr windows. VT IR spectra were recorded using a praying mantis unit. Elemental analysis (C, H, N) was performed on a Elementar vario MICRO cube. Absorption measurements were performed on a PerkinElmer Lambda 35 spectrometer. Cyclic voltammetry (CV) experiments were performed with a Nordic Electrochemistry ECi-200 workstation applying the IR-compensation mode. The data were recorded using Nordic Electrochemistry EC4 DAQ software (version 4.1.90.1) and processed with EC-4 VIEW software (version 1.2.36.1). The CV experiments were performed in a glovebox under argon atmosphere at ambient temperature. The setup comprised a 4 mL vial, equipped with a CHI 104 glassy carbon disc working electrode (CH Instruments, Inc.), a platinum wire counter electrode, and a Ag/AgCl quasi-reference electrode. The surface of the working electrode was polished prior to the measurement. Solutions containing ∼1 mM analyte and 100 mM [*n*Pr_4_N]­[B­(C_6_H_3_(CF_3_)_2_-3,5)_4_] supporting electrolyte were used for the electrochemical analysis. The potentials are reported in volts versus the Fc/Fc^+^ couple, which was used as the internal standard for cell calibration, and determined the end of each measurement.

Crystals for X-ray crystallography were handpicked in a glovebox, coated with Parabar 10,312 and stored on microscope slides. Crystallographic data were collected on a Bruker APEX II DUO diffractometer by using QUAZAR optics and Mo K_α_ radiation (λ = 0.71073 Å). The data collection strategy was determined using COSMO^52^ employing φ and ω scans. Raw data were processed using APEX3 ^53^ and SAINT.^54^ Corrections for absorption effects were applied using SADABS.^55^ The structures were solved by direct methods^56^ and refined against F^2^.^57^ Disorder models are calculated using DSR,^58^ a program included in ShelXle. For compound **4** and **9-Ce** the serious disorder was treated using Platon/Squeeze.^59^

### Cp*_2_Ce­(pz^Me,Me^) (1)

Pyrazole Hpz^Me,Me^ (14.4 mg, 150 μmol, 1.00 equiv) in toluene (4 mL) was slowly added to a solution of Cp*_2_Ce­[N­(SiHMe_2_)_2_] (81.4 mg, 150 μmol, 1.00 equiv) in toluene (1 mL) and stirred for 2 h. A color change from red to blue was observed. The reaction mixture was evaporated to dryness. Crystallization from *n*-hexane yielded blue crystals of **1** (62.4 mg, 123 μmol, 82%). ^1^H NMR (C_6_D_6_, 400.1 MHz, 26 °C): δ = 7.88 (1H, s, pz–C*H*), 1.97 (30H, s, Cp–C*H*_3_), −3.76 (6H, s, pz–C*H*_3_) ppm. ^13^C­{^1^H} NMR (C_6_D_6_, 100.6 MHz, 26 °C): δ = 208.9 (Cp-*C*CH_3_), 158.3 (pz-N*C*), 122.4 (pz-*C*H), 8.1 (pz-*C*H_3_, Cp-*C*H_3_) ppm. DRIFT: ṽ = 3288 (vw), 3099 (w), 2959 (s), 2910 (vs), 2858 (vs), 2725 (w), 2459 (vw), 1514 (s), 1433 (vs), 1382 (m), 1327 (w), 1145 (vw), 1049 (w), 1001 (m), 953 (m), 779 (m), 729 (w), 587 (w), 488 (vw), 434 (w) cm^–1^. Elemental analysis (%) calcd. for C_25_H_37_CeN_2_ (505.70 g/mol): C 59.38, H 7.38, N 5.54; found, C 59.82, H 7.24, N 5.70.

### Cp*_2_Ce­(pz^Me,Me^)­(thf) (1^thf^)

(a) Salt-metathesis route: [Cp*_2_CeCl_2_K­(thf)]_*n*_ (119 mg, 200 μmol, 1.00 equiv) and Kpz^Me,Me^ (26.8 mg, 200 μmol, 1.00 equiv) were suspended in THF (5 mL) and stirred for 2 d. All volatiles were removed, the yellow solid was extracted with *n*-hexane (2 × 5 mL) and filtered. The resulting green solution was evaporated to dryness producing a yellow powder. Crystallization from *n*-hexane yielded green crystals of **1** ^**thf**^ (106 mg, 183 μmol, 92%). Performing the reaction in aliphatic or aromatic solvents resulted in lower yields (62%–76%). ^1^H NMR (C_6_D_6_, 400.1 MHz, 26 °C): δ = 7.09 (1H, s, pz–C*H*), 3.39 (30H, s, Cp–C*H*_3_), −2.04 (6H, s, pz–C*H*_3_), −4.72 (4H, s, OCH_2_C*H*_2_), −13.59 (4H, s, OC*H*_2_) ppm. ^13^C­{^1^H} NMR (C_6_D_6_, 100.6 MHz, 26 °C): δ = 181.7 (Cp-*C*CH_3_), 157.0 (pz-N*C*), 119.6 (pz-*C*H), 10.1 (pz-*C*H_3_), 8.0 (Cp-*C*H_3_) ppm. The THF signals in C_6_D_6_ are only visible in the 2D NMR spectra. ^1^H NMR (THF-*d*_8_, 400.1 MHz, 26 °C): δ = 6.63 (1H, s, pz–C*H*), 3.54 (4H, s, OC*H*_2_), 3.29 (30H, s, Cp–C*H*_3_), 1.78 (4H, m, OCH_2_C*H*_2_), −2.03 (6H, s, pz–C*H*_3_) ppm. ^13^C­{^1^H} NMR (THF-*d*_8_, 100.6 MHz, 26 °C): δ = 178.3 (Cp-*C*CH_3_), 156.8 (pz-N*C*), 119.0 (pz-*C*H), 68.0 (O*C*H_2_), 26.3 (OCH_2_ *C*H_2_), 10.0 (pz-*C*H_3_), 7.7 (Cp-*C*H_3_) ppm. DRIFT: ṽ = 2968 (s), 2904 (vs), 2856 (vs), 2721 (w), 1910 (vw), 1841 (vw), 1764 (vw), 1519 (m), 1428 (m), 1376 (w), 1028 (m), 1007 (m), 955 (w), 872 (m), 780 (m), 726 (w) cm^–1^. Elemental analysis (%) calcd. for C_29_H_45_CeN_2_O (577.81 g/mol): C 60.28, H 7.85, N 4.85; found: C 60.01, H 7.92, N 4.82. (b) Protonolysis route: Hpz^Me,Me^ (14.4 mg, 150 μmol, 1.00 equiv) in THF (4 mL) was slowly added to a solution of Cp*_2_Ce­[N­(SiHMe_2_)_2_] (81.4 mg, 150 μmol, 1.00 equiv) in THF (2 mL) and stirred for 2 h. A color change from red to yellow was observed. The reaction mixture was evaporated to dryness. Crystallization from *n*-hexane yielded **1** ^**thf**^ (62.4 mg, 123 μmol, 82%).

### [Cp*_2_Ce­(pz^Me,Me^·CO_2_)]_2_ (2)

Cp*_2_Ce­(pz^Me,Me^) (55.4 mg, 110 μmol, 1.00 equiv) was dissolved in *n*-pentane (5 mL) and the solution stirred under 1 bar CO_2_ for 60 min. The yellowish solution first turned purple followed by a precipitation of a yellow solid (43.2 mg, 39.3 μmol, 71%). Performing the reaction in C_6_D_6_ followed by addition of THF to the suspension and storing at −40 °C yielded yellow crystals of **2**. DRIFT: ṽ = 3087 (w), 2969 (s), 2906 (s), 2858 (s), 2723 (w), 1729 (m), 1691 (vs), 1640 (vs), 1604 (vs), 1469 (m), 1378 (s), 1320 (vs), 1104 (m), 1035 (m), 979 (m), 858 (m), 803 (m), 755 (m), 599 (w), 478 (w), 413 (w) cm^–1^. Elemental analysis (%) calcd. for C_58_H_74_Ce_2_N_4_O_4_ (1099.42 g/mol): C 56.81, H 6.78, N 5.10; found, C 56.85, H 7.21, N 5.08.

### Cp*_2_Ce­(pz^Ph,Ph^) (3)

(a) Salt-metathesis route: [Cp*_2_CeCl_2_K­(thf)]_*n*_ (119 mg, 200 μmol, 1.00 equiv) and Kpz^Ph,Ph^ (51.7 mg, 200 μmol, 1.00 equiv) were suspended in *n*-hexane (5 mL) and stirred at 40 °C for 1 d. The white precipitate was filtered off and extracted with *n*-hexane (5 mL). The resulting blue solution was evaporated to dryness producing a yellow powder. Crystallization from *n*-hexane yielded yellow crystals of **3** ^**thf**^ and blue crystals of **3**. ^1^H NMR (**3**, C_6_D_6_, 400.1 MHz, 26 °C): δ = 7.60 (1H, s, pz–C*H*), 4.60 (2H, t, *para*–C*H*), 2.95 (4H, t, *meta*–C*H*), 2.11 (30H, s, Cp–C*H*_3_), −5.64 (4H, d, *ortho*–C*H*) ppm. ^1^H NMR (**3**, toluene-*d*_8_, 400.1 MHz, 26 °C): δ = 7.56 (1H, s, pz–C*H*), 4.58 (2H, t, *para*–C*H*), 2.92 (4H, t, *meta*–C*H*), 2.09 (30H, s, Cp–C*H*_3_, overlap with solvent signal), −5.61 (4H, d, *ortho*–C*H*) ppm. ^13^C­{^1^H} NMR (**3**, C_6_D_6_, 100.6 MHz, 26 °C): δ = 219.7 (Cp-*C*CH_3_), 159.6 (pz-N*C*), 124.7 (*meta*-*C*), 123.1 (*para*-*C*), 115.7 (*ipso*-*C* or *ortho*-*C*), 115.6 (*ipso*-*C* or *ortho*-*C*), 114.8 (pz-*C*H), 9.4 (Cp-*C*H_3_) ppm. ^13^C­{^1^H} NMR (**3**, toluene-*d*_8_, 100.6 MHz, 26 °C): δ = 219.0 (Cp-*C*CH_3_), 159.7 (pz-N*C*), 124.6 (*meta*-*C*), 123.1 (*para*-*C*), 115.8 (*ortho*-*C*), 114.8 (pz-*C*H), 9.2 (Cp-*C*H_3_) ppm. The signal of the *ipso* carbon atom overlaps with the solvent signals. DRIFT (**3**): ṽ = 3063 (m), 3040 (m), 2964 (m), 2898 (s), 2855 (s), 2727 (w), 1605 (m), 1467 (vs), 1444 (m), 1404 (m), 1378 (m), 1178 (vw), 1069 (w), 1047 (m), 1025 (m), 968 (s), 903 (vw), 754 (vs), 702 (s), 680 (s), 537 (m), 477 (w), 427 (m) cm^–1^. Elemental analysis (%) calcd. for C_35_H_41_CeN_2_ (629.84 g/mol): C 66.74, H 6.56, N 4.45; found, C 66.94, H 6.58, N 4.61. (b) Protonolysis route: Hpz^Ph,Ph^ (44.1 mg, 200 μmol, 1.00 equiv) in toluene (5 mL) was slowly added to a solution of Cp*_2_Ce­[N­(SiHMe_2_)_2_] (109 mg, 200 μmol, 1.00 equiv) in toluene (1 mL) and stirred at 40 °C for 1 h. A color change from red to blue was observed. The reaction mixture was evaporated to dryness. Crystallization from *n*-hexane yielded **3** (106 mg, 168 μmol, 84%).

### Cp*_2_Ce­(pz^Ph,Ph^)­(thf) (3^thf^)

[Cp*_2_CeCl_2_K­(thf)]_*n*_ (119 mg, 200 μmol, 1.00 equiv) and Kpz^Ph,Ph^ (51.7 mg, 200 μmol, 1.00 equiv) were suspended in THF (5 mL) and stirred for 2 d. The reaction mixture was evaporated to dryness. The resulting yellow powder was extracted with *n*-hexane (2 × 5 mL) and then again evaporated to dryness. Crystallization from a mixture of THF and *n*-hexane yielded **3** ^**thf**^ as colorless crystals (96.1 mg, 137 μmol, 68%). ^1^H NMR (C_6_D_6_, 400.1 MHz, 26 °C): δ = 7.54 (1H, s, pz–C*H*), 5.21 (2H, t, *para*–C*H*), 4.08 (4H, t, *meta*–C*H*), 2.98 (30H, s, Cp–C*H*_3_), −1.31 (4H, s, *ortho*–C*H*), −1.78 (4H, s, OCH_2_C*H*_2_), −5.48 (4H, s, OC*H*_2_) ppm. ^13^C­{^1^H} NMR (C_6_D_6_, 100.6 MHz, 26 °C): δ = 203.3 (Cp-*C*CH_3_), 160.0 (pz-N*C*), 130.1 (*ipso*-*C*), 125.6 (*meta*-*C*), 123.8 (*para*-*C*), 118.7 (*ortho*-*C*), 115.1 (pz-*C*H), 20.4 (OCH_2_ *C*H_2_), 9.3 (Cp-*C*H_3_) ppm. According to 2D NMR spectra the THF-O*C*H_2_ signal appears around 52 ppm, but it is not visible in recorded ^13^C spectra. DRIFT: ṽ = 3065 (w), 2960 (m), 1897 (s), 2857 (s), 2720 (w), 1606 (m), 1466 (s), 1446 (m), 1400 (m), 1222 (w), 1156 (w), 1047 (m), 1024 (s), 970 (s), 910 (m), 876 (s), 757 (vs), 696 (s), 594 (vw), 541 (w), 496 (w), 413 (m) cm^–1^. Elemental analysis (%) calcd. for C_39_H_49_CeN_2_O (701.95 g/mol): C 66.73, H 7.04, N 3.99; found, C 67.10, H 7.13, N 4.02.

### Cp*Ce­(pz^Me,Me^)_2_(thf)_2_ (5)

Cp*CeI_2_(thf)_3_ (149 mg, 200 μmol, 1.00 equiv) and Kpz^Me,Me^ (53.7 mg, 400 μmol, 2.00 equiv) were suspended in *n*-hexane (5 mL) and stirred at 40 °C for 2 d resulting in a yellow suspension. The white precipitate was filtered off and extracted with *n*-hexane (2 × 4 mL). Crystallization from *n*-hexane by evaporation at ambient temperature yielded colorless crystals of **5**. In solution **5** slowly decomposes to **1** ^**thf**^ and unknown side products. ^1^H NMR (C_6_D_6_, 400.1 MHz, 26 °C): δ = 13.06 (2H, s, pz–C*H*), 4.98 (15H, s, Cp–C*H*_3_), 2.11 (18H, s, pz–C*H*_3_ or THF), −1.10 (18H, s, pz–C*H*_3_ or THF), −2.46 (14H, s, THF) ppm. ^1^H NMR (THF-*d*_8_, 400.1 MHz, 26 °C): δ = 12.26 (2H, s, pz–C*H*), 3.87 (15H, s, Cp–C*H*_3_), 3.62 (4H, m, OC*H*_2_), 1.79 (20H, m, pz–C*H*_3_ and OCH_2_C*H*_2_) ppm. The signal at 3.62 ppm should have an integral of 8. There are also signals of the decomposition product **1** ^**thf**^ visible. Due to the impurities some signals as well as the ^13^C NMR spectrum could not be assigned. DRIFT: ṽ = 3090 (w), 2975 (s), 2901 (vs), 2858 (s), 2719 (w), 1513 (s), 1428 (vs), 1036 (s), 1007 (s), 959 (m), 877 (m), 774 (m), 731 (m), 433 (w) cm^–1^. Elemental analysis (%) calcd. for C_28_H_45_CeN_4_O_2_ (609.81 g/mol): C 55.15, H 7.44, N 9.19; found, C 55.36, H 7.37, N 9.22.

### [Cp*Ce­(pz^Me,Me^·CO_2_)­(thf)­(μ-pz^Me,Me^·CO_2_)]_2_ (6)

Cp*Ce­(pz^Me,Me^)_2_(thf)_2_ (61.0 mg, 100 μmol, 1.00 equiv) was dissolved in *n*-pentane (5 mL) and then stirred under 1 bar of CO_2_ atmosphere for 30 min resulting in a yellowish precipitate. Yield: 49.0 mg (39.2 μmol, 78%). Crystals suitable for X-ray diffraction were grown from a THF solution at −40 °C. DRIFT: ṽ = 3097 (vw), 2967 (w), 2927 (w), 2856 (w), 1728 (s), 1668 (vs), 1572 (w), 1468 (m), 1417 (s), 1383 (s), 1360 (s), 1335 (vs), 1290 (s), 1208 (w), 1128 (m), 1038 (m), 981 (w), 841 (w), 795 (m), 759 (m), 928 (vw), 581 (vw), 476 (vw), 416 (vw) cm^–1^. Elemental analysis (%) calcd. for C_52_H_74_Ce_2_N_8_O_10_ (1251.44 g/mol): C 49.91, H 5.96, N 8.95; found, C 47.86, H 5.76, N 9.75. The deviation between theoretical and experimental microanalytical data can be attributed to removal of THF from the complex under vacuum.

### Cp*_2_Ce­(tz^Me,Me^)­(dmap) (7)

DMAP (20.8 mg, 170 μmol, 1.00 equiv) in THF (3 mL) was added to Cp*_2_Ce­[N­(SiHMe_2_)_2_] (92.2 mg, 170 μmol, 1.00 equiv) and stirred for 15 min. Then Htz^Me,Me^ (16.5 mg, 170 μmol, 1.00 equiv) in THF (3 mL) was added and stirred for 2 h. The resulting yellow solution was evaporated to dryness producing a pale-yellow powder. Crystallization from THF yielded yellow crystals of **7** (64.9 mg, 103 μmol, 61%). ^1^H NMR (C_6_D_6_, 400.1 MHz, 26 °C): δ = 3.53 (30H, s, Cp–C*H*_3_), 1.61 (2H, s, DMAP), 0.25 (6H, s, N­(C*H*_3_)_2_), −1.96 (6H, s, tz–C*H*_3_), −12.54 (2H, s, DMAP) ppm. ^13^C­{^1^H} NMR (C_6_D_6_, 125.8 MHz, 26 °C): δ = 180.8 (Cp-*C*CH_3_), 147.5 (tz-N*C*), 101.1 (DMAP), 36.5 (N­(*C*H_3_)_2_), 10.0 (tz-*C*H_3_), 8.3 (Cp-*C*H_3_) ppm. The other signals of the DMAP ligand were not detected. DRIFT: ṽ = 2967 (m), 2903 (s), 2858 (s), 1613 (vs), 1536 (m), 1494 (m), 1453 (s), 1393 (s), 1307 (w), 1233 (s), 1112 (w), 1066 (w), 1001 (s), 952 (w), 872 (vw), 811 (s), 742 (w), 700 (w), 534 (w) cm^–1^. Elemental analysis (%) calcd. for C_31_H_46_CeN_5_ (628.86 g/mol): C 59.21, H 7.37, N 11.14; found, C 59.48, H 7.52, N 10.97.

### Cp*_2_Ce­(tz^Ph,Ph^) (8)

Triazole Htz^Ph,Ph^ (44.3 mg, 200 μmol, 1.00 equiv) in toluene (4 mL) was slowly added to a solution of Cp*_2_Ce­[N­(SiHMe_2_)_2_] (109 mg, 200 μmol, 1.00 equiv) in toluene (1 mL) and stirred for 2 h. A color change from red to blue violet was observed. The reaction mixture was evaporated to dryness. Crystallization from *n*-hexane yielded blue crystals of **8** (89.9 mg, 143 μmol, 71%). ^1^H NMR (C_6_D_6_, 400.1 MHz, 26 °C): δ = 4.64 (2H, t, *para*–C*H*), 3.05 (4H, t, *meta*–C*H*), 2.22 (30H, s, Cp–C*H*_3_), −5.08 (4H, s, *ortho*–C*H*) ppm. ^13^C­{^1^H} NMR (C_6_D_6_, 100.6 MHz, 26 °C): δ = 233.0 (Cp-*C*CH_3_), 164.9 (tz-N*C*), 124.8 (*para*-*C*), 124.6 (*meta*-*C*), 124.2 (*ipso*-*C*), 116.5 (*ortho*-*C*), 10.6 (Cp-*C*H_3_) ppm. DRIFT: ṽ = 3063 (w), 2958 (m), 2909 (s), 2857 (s), 2729 (w), 1605 (vw), 1468 (vs), 1426 (vs), 1407 (m), 1384 (w), 1285 (w), 1177 (w), 1070 (w), 1023 (w), 991 (m), 919 (vw), 790 (w), 728 (s), 694 (s), 488 (vw), 426 (m) cm^–1^. Elemental analysis (%) calcd. for C_34_H_40_CeN_3_ (630.83 g/mol): C 64.74, H 6.39, N 6.66; found, C 65.58, H 6.32, N 6.53. The carbon result is outside the range of analytical purity, but no better elemental analysis could be obtained to date, possibly because of cocrystallized *n*-hexane.

Addition of CO_2_ (1 bar) to a *n*-pentane solution of **8** resulted in the precipitation of an off-white solid. The solid was centrifuged and dried under argon atmosphere. DRIFT: ṽ = 3067 (vw), 2971 (w), 2909 (w), 2858 (w), 1677 (w), 1584 (m), 1529 (m), 1467 (vs), 1427 (vs), 1401 (s), 1355 (s), 1070 (w), 992 (vw), 844 (w), 788 (w), 729 (s), 694 (s), 425 (vw) cm^–1^.

### Cp*_2_Ce­(tz^Ph,Ph^)­(thf) (8^thf^)

[Cp*_2_CeCl_2_K­(thf)]_*n*_ (119 mg, 200 μmol, 1.00 equiv) and Ktz^Ph,Ph^ (51.9 mg, 200 μmol, 1.00 equiv) were suspended in a mixture of *n*-hexane (5 mL) and THF (0.5 mL) and stirred at 40 °C for 2 d. The reaction mixture was evaporated to dryness resulting in a yellow solid. Crystallization from *n*-hexane yielded yellow crystals of **8** ^**thf**^ (54.5 mg, 77.5 μmol, 39%). ^1^H NMR (C_6_D_6_, 400.1 MHz, 26 °C): δ = 5.62 (2H, t, *para*–C*H*), 4.80 (4H, s, *meta*–C*H*), 3.83 (30H, s, Cp–C*H*_3_), 1.51 (4H, s, *ortho*–C*H*), −4.31 (4H, s, OCH_2_C*H*_2_), −13.63 (4H, s, OC*H*_2_) ppm. ^13^C­{^1^H} NMR (C_6_D_6_, 100.6 MHz, 26 °C): δ = 202.7 (Cp-*C*CH_3_), 165.7 (tz-N*C*), 126.0 (*meta*-*C*), 125.7 (*para*-*C*), 120.9 (*ortho*-*C*), 16.1 (OCH_2_ *C*H_2_), 10.3 (Cp-*C*H_3_) ppm. The signals of the *ipso*-*C* and the O*C*H_2_ were not visible in the spectrum. The ^1^H–^13^C HSQC spectrum shows a weak signal for the THF-O*C*H_2_ at around 40 ppm. DRIFT: ṽ = 3065 (m), 2973 (vs), 2905 (vs), 2859 (s), 2723 (w), 1606 (m), 1465 (s), 1424 (vs), 1402 (m), 1284 (vw), 1175 (w), 1069 (m), 1017 (m), 992 (m), 921 (w), 866 (m), 789 (m), 730 (vs), 698 (vs), 593 (vw), 553 (w), 481 (vw), 428 (m) cm^–1^. Elemental analysis (%) calcd. for C_38_H_48_CeN_3_O (702.94 g/mol): C 64.93, H 6.88, N 5.98; found, C 64.98, H 6.48, N 5.87.

### [Cp*_2_Ce­(tet^Ph^)]_3_ (9-Ce)

(a) Salt-metathesis route: [Cp*_2_CeCl_2_K­(thf)]_*n*_ (119 mg, 200 μmol, 1.00 equiv) and Ktet^Ph^ (36.9 mg, 200 μmol, 1.00 equiv) were suspended in toluene (7 mL) and stirred for 5 d. The white precipitate was filtered off and extracted with toluene (3 mL). The resulting yellow solution was evaporated to dryness giving a yellow powder. Crystallization from *n*-hexane yielded yellow crystals of **9-Ce** (86.3 mg, 51.8 μmol, 78%). ^1^H NMR (toluene-*d*_8_, 400.1 MHz, 26 °C): δ = 4.11 (3H, t, *para*–C*H*) 2.64 (96H, m, *meta*–C*H* and Cp–C*H*_3_), −5.64 (6H, d, *ortho*–C*H*) ppm. ^13^C­{^1^H} NMR (toluene-*d*_8_, 100.6 MHz, 26 °C): δ = 192.5 (Cp-*C*CH_3_), 143.9 (tet-N*C*), 126.2 (*para*-*C*H), 123.5 (*meta*-*C*H), 116.6 (*ortho*-*C*H), 114.3 (*ipso*-*C*H), 8.6 (Cp-*C*H_3_) ppm. DRIFT: ṽ = 3061 (m), 3024 (m), 2977 (s), 2945 (vs), 2898 (vs), 2859 (vs), 2724 (m), 2529 (vw), 2449 (vw), 2022 (w), 1952 (w), 1864 (vw), 1810 (w), 1603 (m), 1520 (m), 1494 (m), 1444 (vs), 1378 (m), 1358 (s), 1279 (m), 1176 (m), 1120 (m), 1073 (m), 1012 (m), 921 (w), 785 (m), 728 (vs), 695 (s), 594 (w), 551 (vw), 507 (m), 466 (m) cm^–1^. Elemental analysis (%) calcd. for C_81_H_105_Ce_3_N_12_ (1667.16 g/mol): C 58.36, H 6.35, N 10.08; found, C 58.39, H 6.37, N 10.11. (b) Protonolysis route: tetrazole Htet^Ph^ (29.2 mg, 200 μmol, 1.00 equiv) was suspended in toluene (7 mL) and slowly added to a solution of Cp*_2_Ce­[N­(SiHMe_2_)_2_] (109 mg, 200 μmol, 1.00 equiv) in THF (1 mL). The color changed from red to orange. After being stirred for 3 d the reaction mixture was evaporated to dryness to yield **9** as an orange solid (109 mg, 65.4 μmol, 98%).

### [Cp*_2_La­(tet^Ph^)]_3_ (9-La)

Cp*_2_LaCl_2_K­(thf) (118 mg, 200 μmol, 1.00 equiv) and Kpz^Me,Me^ (36.9 mg, 200 μmol, 1.00 equiv) were suspended in toluene (10 mL) and stirred for 1 day. The solvent was removed and the resulting white solid was extracted with *n*-hexane (2 × 5 mL). The resulting colorless solution was evaporated to dryness giving a white powder. Crystallization from *n*-hexane yielded colorless crystals of **9-La** (92.1 mg, 55.4 μmol, 84%). ^1^H NMR (toluene-*d*_8_, 400.1 MHz, 26 °C): δ = 8.70 (6H, d, *ortho*–C*H*), 7.48 (6H, t, *meta*-C*H*), 7.28 (3H, t, *para*–C*H*), 1.98 (90H, s, Cp–C*H*_3_) ppm. ^13^C­{^1^H} NMR (toluene-*d*_8_, 100.6 MHz, 26 °C): δ = 161.1 (tet-N*C*), 131.2 (*para*-*C*H), 129.1 (*meta-C*H, overlap with solvent signal), 128.4 (*ortho*-*C*H), 127.1 (*ipso-C*), 120.5 (Cp-*C*CH_3_), 11.7 (Cp-*C*H_3_) ppm. Elemental analysis (%) calcd. for C_81_H_105_La_3_N_12_ (1663.53 g/mol): C 58.48, H 6.36, N 10.10; found, C 58.33, H 6.26, N 9.96.

### Cp*_2_La­(pz^Me,Me^)­(thf) (1^thf^-La)

Cp*_2_LaCl_2_K­(thf) (150 mg, 254 μmol, 1.00 equiv) and Kpz^Me,Me^ (34.0 mg, 254 μmol, 1.00 equiv) were suspended in THF (5 mL) and stirred for 3 d. The reaction mixture was evaporated to dryness. The resulting solid was extracted with *n*-hexane (2 × 5 mL) followed by evaporation of the solvent. Crystallization from *n*-hexane yielded colorless crystals of **1** ^**thf**^ **-La** (102 mg, 177 μmol, 70%). ^1^H NMR (C_6_D_6_, 400.1 MHz, 26 °C): δ = 6.19 (1H, s, pz–C*H*), 3.73 (4H, m, OC*H*_2_), 2.34 (6H, s, pz–C*H*_3_), 1.96 (30H, s, Cp–C*H*_3_), 1.35 (4H, m, OCH_2_C*H*_2_) ppm. ^13^C­{^1^H} NMR (C_6_D_6_, 100.6 MHz, 26 °C): δ = 143.8 (pz-N*C*), 118.3 (Cp-*C*CH_3_), 110.1 (pz-*C*H), 70.8 (O*C*H_2_), 25.4 (OCH_2_ *C*H_2_), 14.0 (pz-*C*H_3_), 11.0 (Cp-*C*H_3_) ppm. DRIFT: ṽ = 3110 (vw), 2966 (s), 2903 (vs), 2857 (vs), 2722 (w), 1519 (m), 1427 (s), 1377 (m), 1314 (w), 1245 (vw), 1177 (vw), 1089 (w), 1027 (s), 1007 (m), 955 (m), 871 (m), 780 (m), 726 (m), 672 (vw), 589 (vw), 431 (w) cm^–1^. Elemental analysis (%) calcd. for C_29_H_45_LaN_2_O (576.60 g/mol): C 60.41, H 7.87, N 4.86; found, C 60.55, H 7.44, N 4.92.

### Reaction of 1^thf^ with Htz^Me,Me^

Cp*_2_Ce­(pz^Me,Me^)­(thf) (14.4 mg, 25 μmol, 1.00 equiv) in THF-*d*_8_ (0.2 mL) was slowly added to Htz^Me,Me^ (2.4 mg, 25 μmol, 1.00 equiv) in THF-*d*_8_ (0.3 mL) and stirred for 30 min resulting in a white suspension. The ^1^H NMR spectrum indicated the formation of HCp* among other unidentified products.

### Reaction of 1^thf^ with Htet^Ph^

Tetrazole Htet^Ph^ (2.9 mg, 20 μmol, 1.00 equiv) in toluene (2 mL) was slowly added to Cp*_2_Ce­(pz^Me,Me^)­(thf) (11.6 mg, 20 μmol, 1.00 equiv) in toluene (2 mL) and stirred for 30 min. The reaction mixture was evaporated to dryness. The ^1^H NMR spectrum indicated the formation of HCp* among other unidentified products.

### Oxidation of 1^thf^ with C_2_Cl_6_

C_2_Cl_6_ (2.4 mg, 10 μmol, 0.50 equiv) in C_6_D_6_ (0.25 mL) was added to Cp*_2_Ce­(pz^Me,Me^)­(thf) (11.6 mg, 20.0 μmol, 1.00 equiv) in C_6_D_6_ (0.25 mL) in a J. Young valved NMR tube resulting in a dark blue solution. The ^1^H NMR spectrum indicated the formation of Cp*_2_.^60^ Cooling a *n*-hexane solution of the reaction mixture to −40 °C resulted in the formation of a few colorless crystals of Cp*_4_Ce_4_Cl_6_(pz^Me,Me^)_2_(thf) (**10**).

### Oxidation of 1^thf^-La with C_2_Cl_6_

C_2_Cl_6_ (2.4 mg, 10 μmol, 0.50 equiv) in C_6_D_6_ (0.25 mL) was added to Cp*_2_La­(pz^Me,Me^)­(thf) (11.5 mg, 20.0 μmol, 1.00 equiv) in C_6_D_6_ (0.25 mL) in a J. Young valved NMR tube resulting in a colorless solution. The ^1^H NMR spectrum after 10 min showed primarily signals of the starting material and small signals of Cp*_2_. The ratio of the Cp*_2_ signals compared to the starting material increased over time.

### Oxidation of KCp* with C_2_Cl_6_

C_2_Cl_6_ (3.6 mg, 15 μmol, 0.50 equiv) in C_6_D_6_ (0.5 mL) was added to KCp* (5.2 mg, 30 μmol, 1.00 equiv) and stirred for 2 h. The ^1^H NMR spectrum indicated the formation of Cp*_2_.

### 3,5-Diphenylpyrazole

Hydrazine hydrate (3.00 g, 60.0 mmol, 1.20 equiv) in EtOH (50 mL) was added to 1,3-diphenylpropane-1,3-dione (11.2 g, 50.0 mmol, 1.00 equiv) in EtOH (100 mL) and heated for 3 h under reflux. Then, saturated NaHCO_3_ solution (100 mL) was added resulting in the precipitation of a white solid. The mixture was concentrated under reduced pressure and subsequently extracted with Et_2_O (4 × 100 mL). The solution was dried over Na_2_SO_4_, filtered and the solvent was removed under reduced pressure. The yield was not determined. The ^1^H NMR data were comparable to the data reported in the literature.^61^

## Supplementary Material



## Notes

Supporting figures, detailed crystallographic data, spectroscopic data (NMR), and analytical details (PDF)
