# Supplementary material for: Cerium(III) Azolate Promoted CO2 Insertion
Source: Inorg Chem. 2025 Nov 5;64(45):22238–50. doi: 10.1021/acs.inorgchem.5c03187 (PMC12628294; doi:10.1021/acs.inorgchem.5c03187)
Supplement: Supplementary file 1 [file ic5c03187_si_001.pdf]

Supporting Information

Cerium(III) Azolate Promoted CO_2 Insertion

Jonas Riedmaier, Cäcilia Maichle-Mössmer, and Reiner Anwander*

Institut für Anorganische Chemie, Eberhard Karls Universität Tübingen, Auf der Morgenstelle 18, 72076 Tübingen, Germany

*Email: reiner.anwander@uni-tuebingen.de

Table of Contents

Crystallographic Data S3

NMR Spectra (solvent signals are marked with *, impurities with #) S17

IR Spectra S43

Thermogravimetry S48

Absorption Spectra S49

Cyclic Voltammetry Experiments S57

Crystallographic Data

Table S1. Crystallographic Data for compounds **1**, **1^{thf}** and **2**.

	**1**	**1^{thf}**	**2**
CCDC	2471873	2471875	2471874
formula	$C_{25}H_{37}CeN_2$	$C_{29}H_{45}CeN_2O$	$C_{58}H_{74}Ce_2D_6N_4O_4$
M_r [g/mol]	505.68	577.79	1183.53
color/shape	duke blue/plate	olive green/plate	yellow/block
crystal dimensions [mm]	0.286 × 0.170 × 0.100	0.196 × 0.132 × 0.096	0.195 × 0.188 × 0.127
crystal system	monoclinic	monoclinic	orthorhombic
space group	*C*2/*c*	$P2_1/c$	*Pbca*
a [Å]	12.8666(3)	16.824(5)	16.9635(11)
b [Å]	16.9309(4)	10.168(3)	17.3580(11)
c [Å]	10.9497(3)	16.359(5)	18.1106(12)
α [°]	90	90	90
β [°]	99.7350(10)	90.892(5)	90
γ [°]	90	90	90
V [Å^3]	2350.97(10)	2798.3(14)	5341.0(6)
Z	4	4	4
T [K]	100(2)	100(2)	100(2)
λ [Å]	0.71073	0.71073	0.71073
ρ_{calcd} [g/cm^3]	1.429	1.371	1.434
μ [mm^{-1}]	1.948	1.649	1.730
F (000)	1036	1196	2344
θ range [°]	2.006–36.414	1.750–29.147	2.019–28.695
unique reflections	5748	7338	6893
total reflections	50166	55635	52052
R_1[a]/wR_2[b] (I>2σ)	0.0189/0.0434	0.0555/0.1502	0.0405/0.1012
R_1[a]/wR_2[b] (all data)	0.0236/0.0455	0.0711/0.1633	0.0522/0.1108
GOF[c]	1.072	1.063	1.033

[a]$R_1 = \Sigma(||F_0| - |F_c||) / \Sigma|F_0|$, $F_0 > 4\sigma(F_0)$, [b]$wR_2 = \{\Sigma[w(F_0^2 - F_c^2)^2 / \Sigma[w(F_0^2)^2]\}^{1/2}$. [c]$GOF = [\Sigma w(F_0^2 - F_c^2)^2 / (n_0 - n_p)]^{1/2}$.

Table S2. Crystallographic Data for compounds **3**, **3**^{thf} and **4**.

	**3**	**3**^{thf}	**4**
CCDC	2471876	2471877	2471882
formula	$C_{35}H_{41}CeN_2$	$C_{39}H_{49}CeN_2O$	$C_{71}H_{82}Ce_2N_4O_2$
M_r [g/mol]	629.82	701.92	1303.64
color/shape	blue/block	colorless/column	red/column
crystal dimensions [mm]	0.162 × 0.141 × 0.064	0.351 × 0.164 × 0.121	0.194 × 0.117 × 0.066
crystal system	tetragonal	monoclinic	monoclinic
space group	$P4_12_12$	$C2/c$	$C2/c$
a [Å]	10.1799(4)	15.9991(16)	36.697(16)
b [Å]	10.1799(4)	13.5300(13)	15.276(7)
c [Å]	29.1385(19)	31.682(3)	28.855(13)
α [°]	90	90	90
β [°]	90	98.2080(10)	119.932(6)
γ [°]	90	90	90
V [$Å^3$]	3019.6(3)	6788.0(12)	14018(11)
Z	4	8	8
T [K]	100(2)	100(2)	100(2)
λ [Å]	0.71073	0.71073	0.71073
ρ_{calcd} [g/cm^3]	1.385	1.374	1.235
μ [mm^{-1}]	1.532	1.373	1.325
F (000)	1292	2904	5344
θ range [°]	2.119–26.429	1.980–28.759	1.281–30.621
unique reflections	3101	8824	21534
total reflections	59920	68008	148526
R_1[a]/wR_2[b] (I>2σ)	0.0220/0.0432	0.0273/0.0616	0.0473/0.1034
R_1[a]/wR_2[b] (all data)	0.0255/0.0446	0.0303/0.0631	0.0916/0.1242
GOF[c]	1.163	1.063	1.003

[a]$R_1 = \Sigma(||F_0| - |F_c||) / \Sigma|F_0|$, $F_0 > 4\sigma(F_0)$, [b]$wR_2 = \{\Sigma[w(F_0^2 - F_c^2)^2 / \Sigma[w(F_0^2)^2]\}^{1/2}$. [c]$GOF = [\Sigma w(F_0^2 - F_c^2)^2 / (n_0 - n_p)]^{1/2}$.

Table S3. Crystallographic Data for compounds **5**, **6** and **7**.

	**5**	**6**	**7**
CCDC	2471871	2471870	2471878
formula	$C_{28}H_{45}CeN_4O_2$	$C_{60}H_{90}Ce_2N_8O_{12}$	$C_{31}H_{46}CeN_5{\cdot}0.5\ (OC_4H_{10})$
M_r [g/mol]	609.80	1395.63	665.90
color/shape	colorless/block	yellow/plate	colorless/needle
crystal dimensions [mm]	0.411 × 0.342 × 0.212	0.186 × 0.116 × 0.077	0.175 × 0.114 × 0.064
crystal system	monoclinic	triclinic	monoclinic
space group	$P2_1$	$P\bar{1}$	$P2_1/c$
a [Å]	9.8120(6)	10.417(4)	9.8366(5)
b [Å]	16.0139(10)	12.333(5)	16.3771(8)
c [Å]	10.0322(6)	14.803(6)	41.0034(19)
α [°]	90	100.881(5)	90
β [°]	112.4120(10)	106.419(5)	94.4780(10)
γ [°]	90	110.924(5)	90
V [$Å^3$]	1457.28(15)	1612.5(12)	6585.3(6)
Z	2	1	8
T [K]	100(2)	100(2)	100(2)
λ [Å]	0.71073	0.71073	0.71073
ρ_{calcd} [g/cm^3]	1.390	1.437	1.343
μ [mm^{-1}]	1.591	1.457	1.412
F (000)	630	718	2768
θ range [°]	2.196–30.477	1.516–25.680	1.339–25.587
unique reflections	8829	6119	12330
total reflections	26407	13439	84836
R_1[a]/wR_2[b] (I>2σ)	0.0222/0.0527	0.0432/0.0898	0.0428/0.0742
R_1[a]/wR_2[b] (all data)	0.0234/0.0534	0.0661/0.0978	0.0832/0.0882
GOF[c]	1.050	0.996	1.010

[a]$R_1 = \Sigma(||F_0| - |F_c||) / \Sigma|F_0|$, $F_0 > 4\sigma(F_0)$, [b]$wR_2 = \{\Sigma[w(F_0^2 - F_c^2)^2 / \Sigma[w(F_0^2)^2]\}^{1/2}$. [c]$GOF = [\Sigma w(F_0^2 - F_c^2)^2 / (n_0 - n_p)]^{1/2}$.

Table S4. Crystallographic Data for compounds **8**, **8^thf^** and **9-Ce**.

	**8**	**8^{thf}**	**9-Ce**
CCDC	2471881	2471883	2471884
formula	$C_{34}H_{40}CeN_3·0.25\ (C_6H_{14})$	$C_{38}H_{48}CeN_3O$	$C_{84}H_{112}Ce_3N_{12}$
M_r [g/mol]	652.35	702.91	1710.21
color/shape	blue/block	colorless/column	yellow/column
crystal dimensions [mm]	0.214 × 0.198 × 0.078	0.249 × 0.138 × 0.126	0.597 × 0.239 × 0.154
crystal system	triclinic	monoclinic	monoclinic
space group	$P\bar{1}$	$P2_1/c$	$P2_1/c$
a [Å]	10.1276(4)	14.4006(9)	11.3865(17)
b [Å]	17.0026(7)	13.5860(9)	26.641(4)
c [Å]	20.2691(8)	17.1113(11)	28.957(4)
α [°]	71.5430(10)	90	90
β [°]	87.1460(10)	94.853(3)	94.416(3)
γ [°]	75.5410(10)	90	90
V [$Å^3$]	3204.2(2)	3335.8(4)	8758(2)
Z	4	4	4
T [K]	100(2)	100(2)	100(2)
λ [Å]	0.71073	0.71073	0.71073
ρ_{calcd} [g/cm^3]	1.352	1.400	1.297
μ [mm^{-1}]	1.447	1.398	1.579
F (000)	1342	1452	3496
θ range [°]	1.303–30.729	1.419–29.130	1.040–28.754
unique reflections	19766	8955	22704
total reflections	154630	187258	167466
R_1[a]/wR_2[b] (I>2σ)	0.0275/0.0580	0.0672/0.1635	0.0460/0.1031
R_1[a]/wR_2[b] (all data)	0.0362/0.0620	0.0735/0.1659	0.0548/0.1077
GOF[c]	1.053	1.403	1.119

[a]$R_1 = \Sigma(||F_0| - |F_c||) / \Sigma|F_0|$, $F_0 > 4\sigma(F_0)$, [b]$wR_2 = \{\Sigma[w(F_0^2 - F_c^2)^2 / \Sigma[w(F_0^2)^2]\}^{1/2}$. [c]$GOF = [\Sigma w(F_0^2 - F_c^2)^2 / (n_0 - n_p)]^{1/2}$.

Table S5. Crystallographic Data for compounds **9-La**, **1[thf]-La** and **10**.

	**9-La**	**1[thf]-La**	**10**
CCDC	2471880	2471872	2471879
formula	$C_{90}H_{126}La_3N_{12}$	$C_{29}H_{45}LaN_2O$	$C_{63}H_{103}Ce_4Cl_6N_4O$
M_r [g/mol]	1792.75	576.58	1705.67
color/shape	colorless/block	colorless/block	colorless/plate
crystal dimensions [mm]	0.193 × 0.166 × 0.133	0.191 × 0.154 × 0.124	0.309 × 0.194 × 0.187
crystal system	monoclinic	monoclinic	monoclinic
space group	$P2_1/c$	$P2_1/c$	$P2_1/c$
a [Å]	11.3342(7)	16.8171(8)	14.9419(14)
b [Å]	26.6060(17)	10.1888(5)	25.021(2)
c [Å]	28.7539(18)	16.3736(8)	20.7964(19)
α [°]	90	90	90
β [°]	94.0570(10)	91.5130(10)	100.2930(10)
γ [°]	90	90	90
V [$Å^3$]	8649.2(9)	2804.6(2)	7649.8(12)
Z	4	4	4
T [K]	100(2)	100(2)	100(2)
λ [Å]	0.71073	0.71073	0.71073
ρ_{calcd} [g/cm^3]	1.377	1.366	1.481
μ [mm^{-1}]	1.505	1.545	2.581
F (000)	3684	1192	3404
θ range [°]	1.044–28.289	2.337–30.548	1.755–28.422
unique reflections	21465	8559	19134
total reflections	137126	37450	116005
R_1[a]/wR_2[b] (I>2σ)	0.0276/0.0653	0.0360/0.0875	0.0535/0.1218
R_1[a]/wR_2[b] (all data)	0.0337/0.0689	0.0440/0.0925	0.0790/0.1433
GOF[c]	1.041	1.044	1.165

[a]$R_1 = \Sigma(||F_0| - |F_c||) / \Sigma|F_0|$, $F_0 > 4\sigma(F_0)$, [b]$wR_2 = \{\Sigma[w(F_0^2 - F_c^2)^2 / \Sigma[w(F_0^2)^2]\}^{1/2}$. [c]$GOF = [\Sigma w(F_0^2 - F_c^2)^2 / (n_0 - n_p)]^{1/2}$.

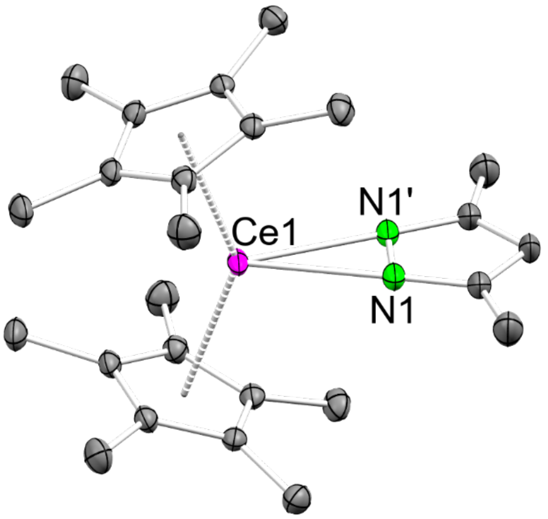

Figure S1. Crystal structure of Cp*$_2$Ce(pz^{Me,Me}) (**1**). Ellipsoids are shown at a 50% probability level. Hydrogen atoms are omitted for clarity. Selected interatomic distances [Å]: Ce1–Ct 2.492, Ce1–N1 2.4334(8). Selected angles [°]: Ct–Ce1–Ct 140.46.

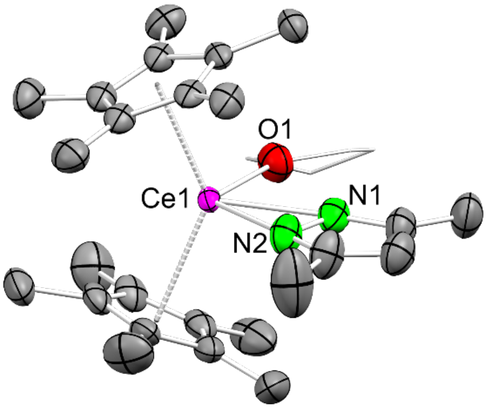

Figure S2. Crystal structure of Cp*$_2$Ce(pz^{Me,Me})(thf) (**1^{thf}**). Ellipsoids are shown at a 50% probability level. Hydrogen atoms and disordered Cp* and THF are omitted for clarity. Part of the THF is displayed as a wireframe model for improved visibility. Selected interatomic distances [Å]: Ce1–Ct1 2.532, Ce1–Ct2 2.535, Ce1–N1 2.468(5), Ce1–N2 2.411(6), Ce1–O1 2.601(5). Selected angles [°]: Ct1–Ce1–Ct2 134.36.

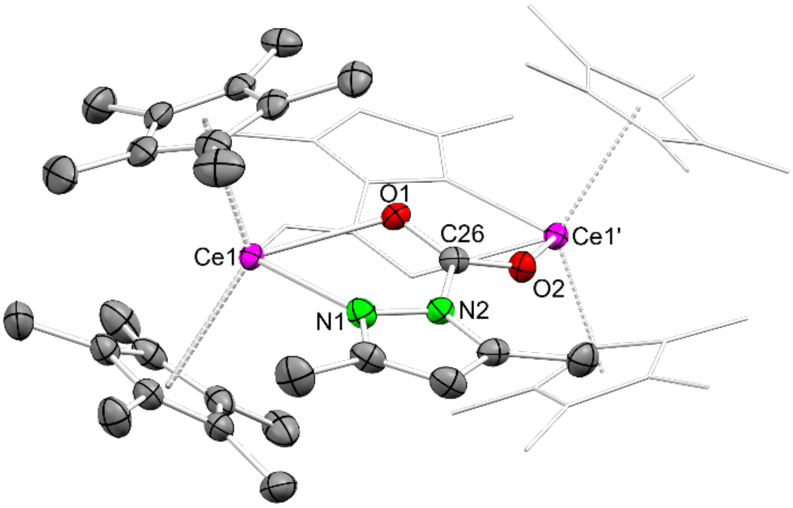

Figure S3. Crystal structure of [Cp*$_2$Ce(pz^{Me,Me}·CO_2)]$_2$ (**2**). Ellipsoids are shown at a 50% probability level. Hydrogen atoms and lattice C_6D_6 are omitted for clarity. A part of the molecule is displayed as a wireframe model for improved visibility. Selected interatomic distances [Å]: Ce1–Ct1 2.542, Ce1–Ct2 2.577, Ce1–N1 2.708(3), Ce1–O1 2.469(3), Ce1–O2 2.532(2), C26–N2 1.441(4), C26–O1 1.242(4), C26–O2 1.247. Selected angles [°]: Ct–Ce1–Ct 129.90, N1–Ce1–O1 (bite) 60.03(9).

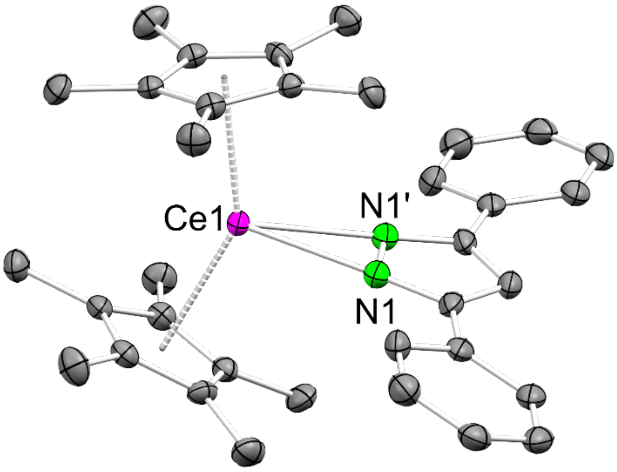

Figure S4. Crystal structure of Cp*$_2$Ce(pz^{Ph,Ph}) (**3**). Ellipsoids are shown at a 50% probability level. Hydrogen atoms are omitted for clarity. Selected interatomic distances [Å]: Ce1–Ct 2.493, Ce1–N1 2.444(3). Selected angles [°]: Ct–Ce1–Ct 145.40.

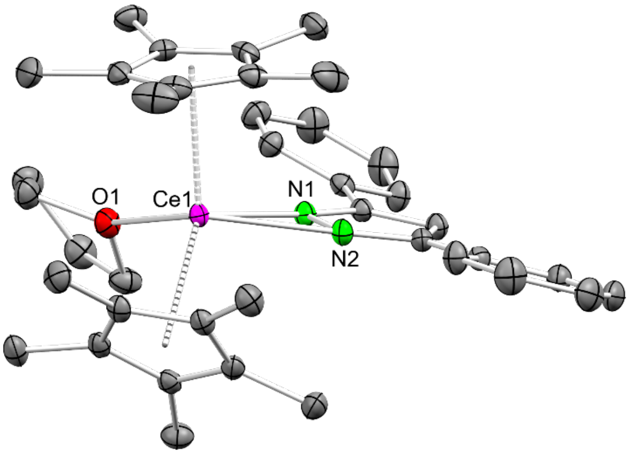

Figure S5. Crystal structure of Cp*$_2$Ce(pz^{Ph,Ph})(thf) (**3^{thf}**). Ellipsoids are shown at a 50% probability level. Hydrogen atoms are omitted for clarity. Selected interatomic distances [Å]: Ce1–Ct1 2.539, Ce1–Ct2 2.536, Ce1–N1 2.5027(16), Ce1–N2 2.4788(16), Ce1–O1 2.5807(14). Selected angles [°]: Ct1–Ce1–Ct2 133.09.

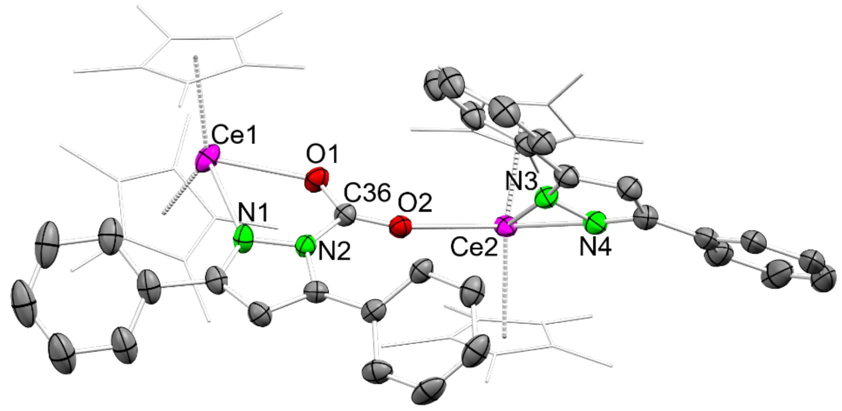

Figure S6. Crystal structure of Cp*$_2$Ce(μ-pz^{Ph,Ph}·CO_2)CeCp*$_2$(pz^{Ph,Ph}) (**4**). Ellipsoids are shown at a 50% probability level. Hydrogen atoms and disordered Cp* are omitted for clarity. A part of the molecule is displayed as a wireframe model for improved visibility. Selected interatomic distances [Å]: Ce1–Ct1 2.495, Ce1–Ct2 2.533, Ce2–Ct3 2.557, Ce2–Ct4 2.555, Ce1–N1 2.563(3), Ce1–O1 2.429(2), Ce2–N3 2.498(3), Ce2–N4 2.509(3), Ce2–O2 2.588(2), C36–N2 1.457(4), C36–O1 1.254(4), C36–O2 1.230(4). Selected angles [°]: Ct1–Ce1–Ct2 138.29, Ct3–Ce2–Ct4 130.77, N1–Ce1–O1 (bite) 61.59(8).

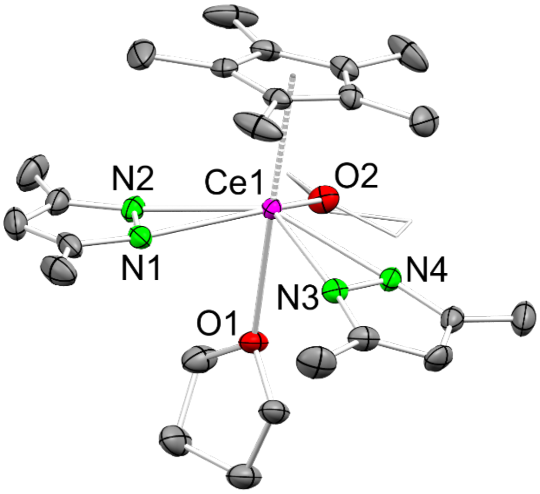

Figure S7. Crystal structure of Cp*Ce(pz^{Me,Me})$_2$(thf)$_2$ (**5**). Ellipsoids are shown at a 50% probability level. Hydrogen atoms are omitted for clarity. Part of one THF is displayed as a wireframe model for improved visibility. Selected interatomic distances [Å]: Ce1–Ct 2.505, Ce1–N1 2.473(3), Ce1–N2 2.483(4), Ce1–N3 2.432(2), Ce1–N4 2.500(3), Ce1–O1 2.6195(18), Ce1–O2 2.591(3). Selected angles [°]: Ct–Ce1–O1 174.89.

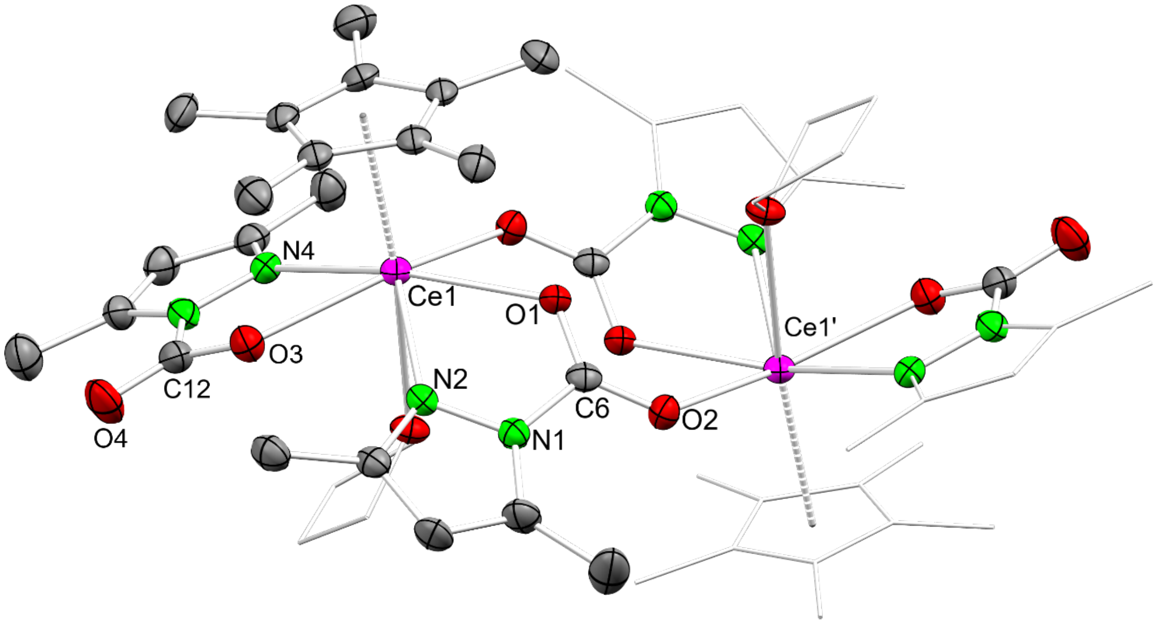

Figure S8. Crystal structure of [Cp*Ce(pz^{Me,Me}·CO_2)$_2$(thf)]$_2$ (**6**). Ellipsoids are shown at a 50% probability level. Hydrogen atoms and lattice THF are omitted for clarity. A part of the molecule is displayed as a wireframe model for improved visibility. Selected interatomic distances [Å]: Ce1–Ct 2.522, Ce1–N2 2.743(4), Ce1–N4 2.727(4), Ce1–O1 2.479(3), Ce1'–O2 2.564(3), Ce1–O3 2.389(3), C6–O1 1.246(5), C6–O2 1.250(5), C12–O3 1.263(6), C12–O4 1.222(5). Selected angles [°]: N2–Ce1–O1 (bite) 59.64(10), N4–Ce1–O3 (bite) 61.53(11). ‘ = -x+1, -y+1, -z+1

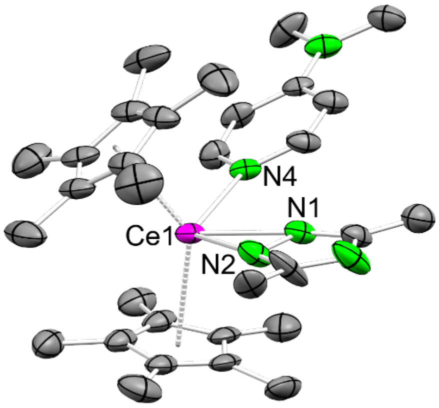

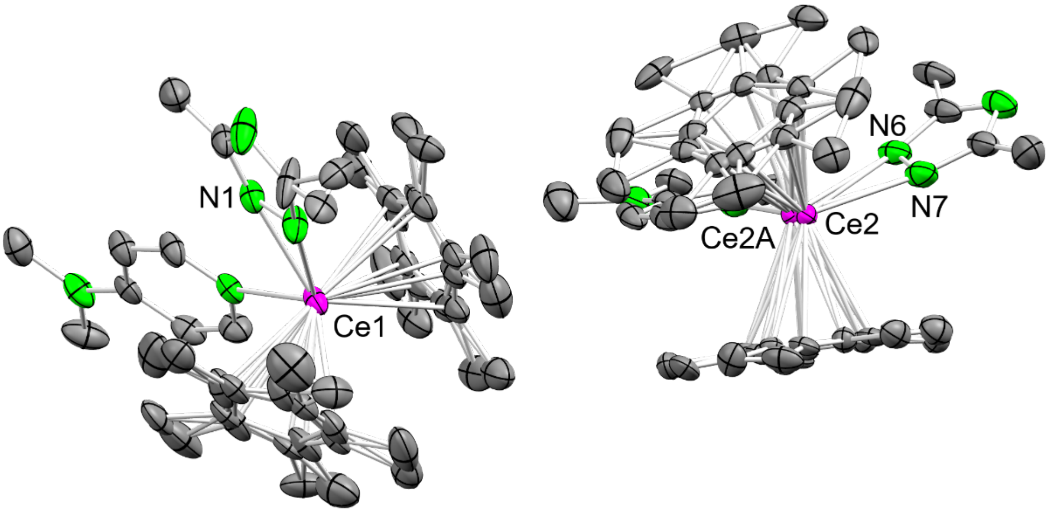

Figure S9. Crystal structure of $Cp^*_2Ce(tz^{Me,Me})(dmap)$ (**7**). Ellipsoids are shown at a 50% probability level. Top: Molecule 1: Hydrogen atoms, the second molecule in the asymmetric unit, disordered Cp* and lattice Et_2O are omitted for clarity. Selected interatomic distances [Å]: Ce1–Ct1 2.526, Ce1–Ct2 2.548, Ce1–N1 2.537(4), Ce1–N2 2.458(4), Ce1–N4 2.592(4). Selected angles [°]: Ct1–Ce1–Ct2 135.91. Bottom: the two molecules in the asymmetric unit, showing disorder. Selected interatomic distances [Å] for the second, severely disordered molecule: Ce2–N6 2.507(5), Ce2A–N6 2.549(5), Ce2–N7 2.375(5), Ce2A–N7 2.623(5).

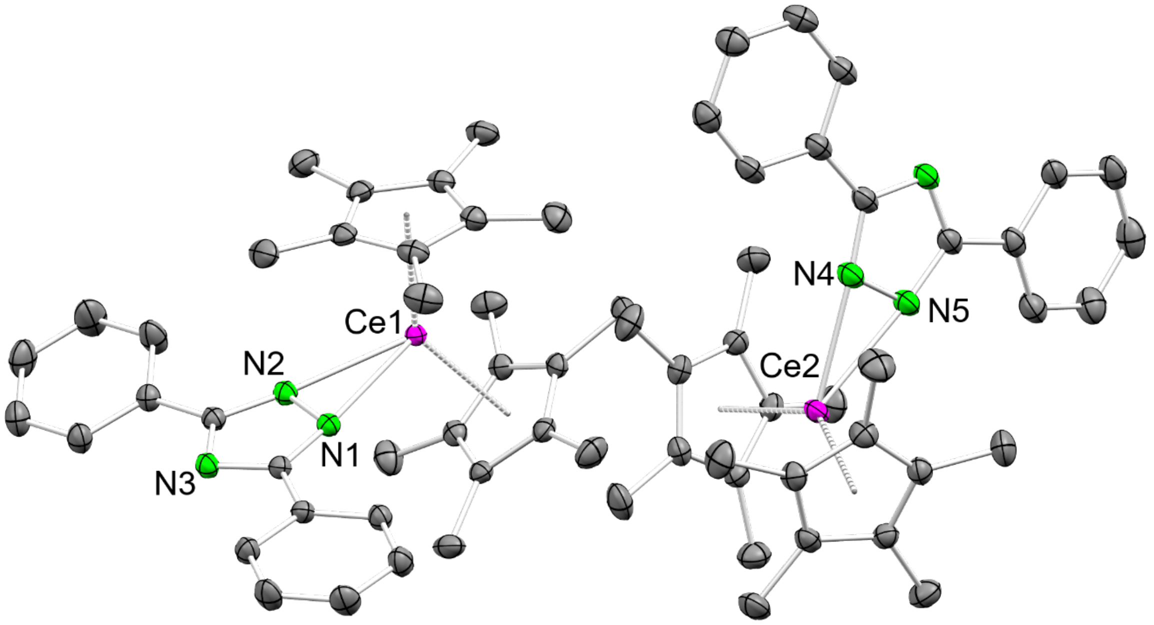

Figure S10. Crystal structure of $Cp*_2Ce(tz^{Ph,Ph})$ (**8**) with two molecules in the asymmetric unit. Ellipsoids are shown at a 50% probability level. Hydrogen atoms and lattice *n*-hexane are omitted for clarity. Selected interatomic distances [Å]: Ce–Ct 2.465–2.487, Ce1–N1 2.4611(15), Ce1–N2 2.4889(15), Ce2–N4 2.4777(16), Ce2–N5 2.4947(16). Selected angles [°]: Ct–Ce–Ct 140.10–141.95.

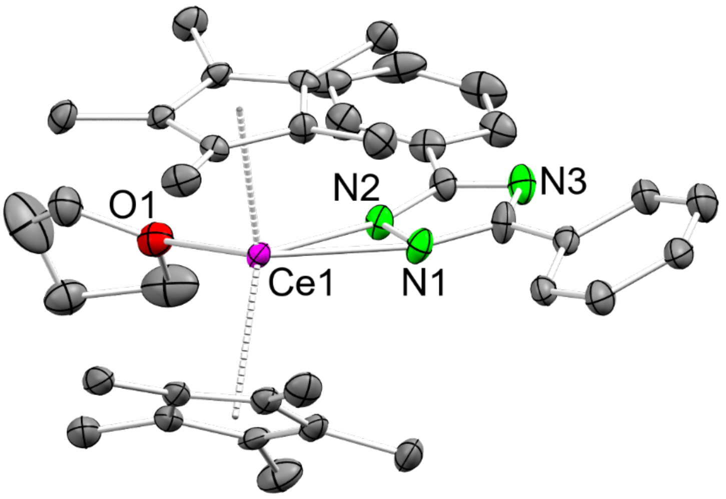

Figure S11. Crystal structure of $Cp*_2Ce(tz^{Ph,Ph})(thf)$ (**8**$^{\mathbf{thf}}$). Ellipsoids are shown at a 50% probability level. Hydrogen atoms and the disordered THF and phenyl ring are omitted for clarity. Selected interatomic distances [Å]: Ce1–Ct1 2.540, Ce1–Ct2 2.553, Ce1–N1 2.501(5), Ce1–N2 2.538(5), Ce1–O1 2.578(5). Selected angles [°]: Ct1–Ce1–Ct2 134.95.

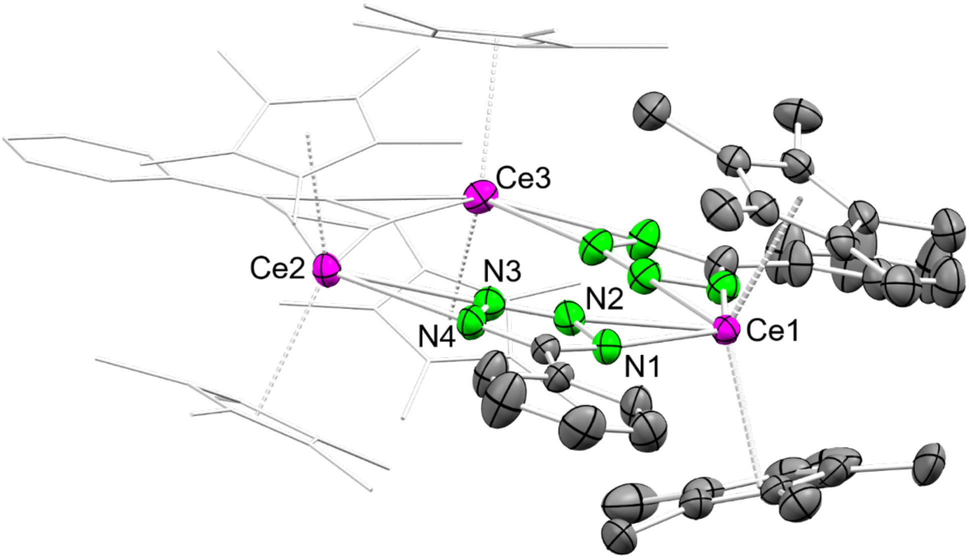

Figure S12. Crystal structure of $[Cp^*{}_2Ce(tet^{Ph})]_3$ (**9-Ce**). Ellipsoids are shown at a 50% probability level. Hydrogen atoms, lattice *n*-hexane and the disordered phenyl ring are omitted for clarity. A part of the molecule is displayed as a wireframe model for improved visibility. Selected interatomic distances [Å]: Ce–Ct 2.519–2.549, Ce1–N1 2.723(3), Ce1–N2 2.612(3), Ce2–N3 2.606(3), Ce2–N4 2.728(3), Ce1–Ce2 6.4458(10), Ce1–Ce3 6.5247(10), Ce2–Ce3 6.5201(10). Selected angles [°]: Ct–Ce–Ct 136.40–137.94, Ce1–Ce2–Ce3 60.42.

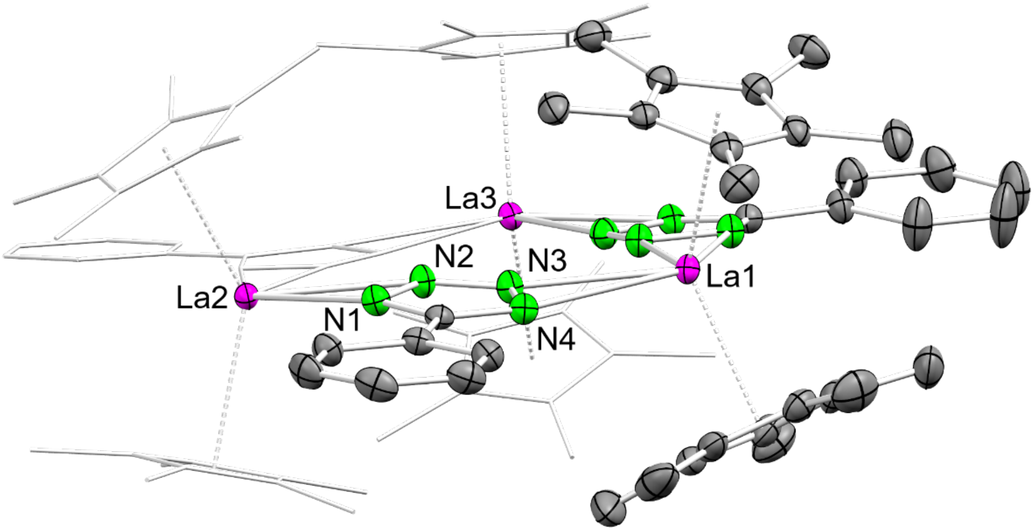

Figure S13. Crystal structure of $[Cp^*{}_2La(tet^{Ph})]_3$ (**9-La**). Ellipsoids are shown at a 50% probability level. Hydrogen atoms, lattice *n*-hexane and the disordered phenyl ring are omitted for clarity. A part of the molecule is displayed as a wireframe model for improved visibility. Selected interatomic distances [Å]: La–Ct 2.550–2.573, La1–N1 2.8151(18), La1–N2 2.6190(18), La2–N3 2.6838(18), La2–N4 2.6444(18), La1–La2 6.5419(4), La1–La3 6.5370(4), La2–La3 6.4652(4). Selected angles [°]: Ct–La–Ct 136.12–137.11, La1–La2–La3 60.34.

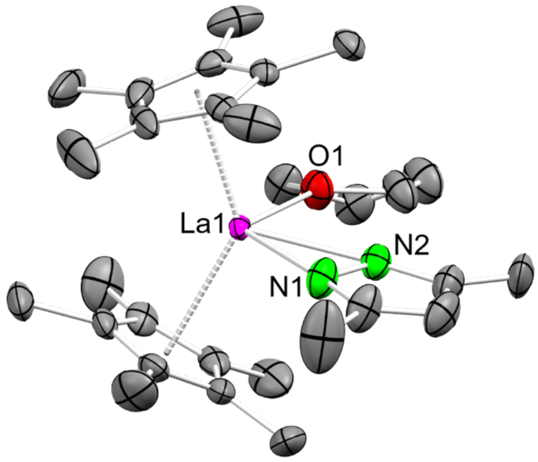

Figure S14. Crystal structure of $Cp^{*}_{2}La(pz^{Me,Me})(thf)$ ($\mathbf{1^{thf}}$**-La**). Ellipsoids are shown at a 50% probability level. Hydrogen atoms and disordered THF are omitted for clarity. Selected interatomic distances [Å]: La1–Ct1 2.568, La1–Ct2 2.570, La1–N1 2.431(3), La1–N2 2.502(3), La1–O1 2.615(2). Selected angles [°]: Ct1–La1–Ct2 134.84.

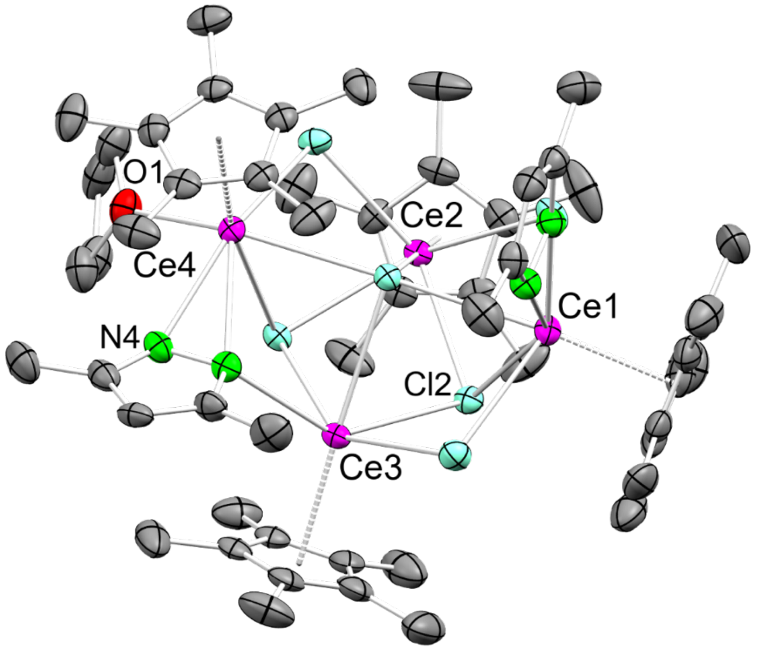

Figure S15. Crystal structure of $Cp^{*}_{4}Ce_{4}Cl_{6}(pz^{Me,Me})_{2}(thf)$ (**10**). Ellipsoids are shown at a 50% probability level. Hydrogen atoms, disordered Cp* and lattice *n*-hexane molecules are omitted for clarity.

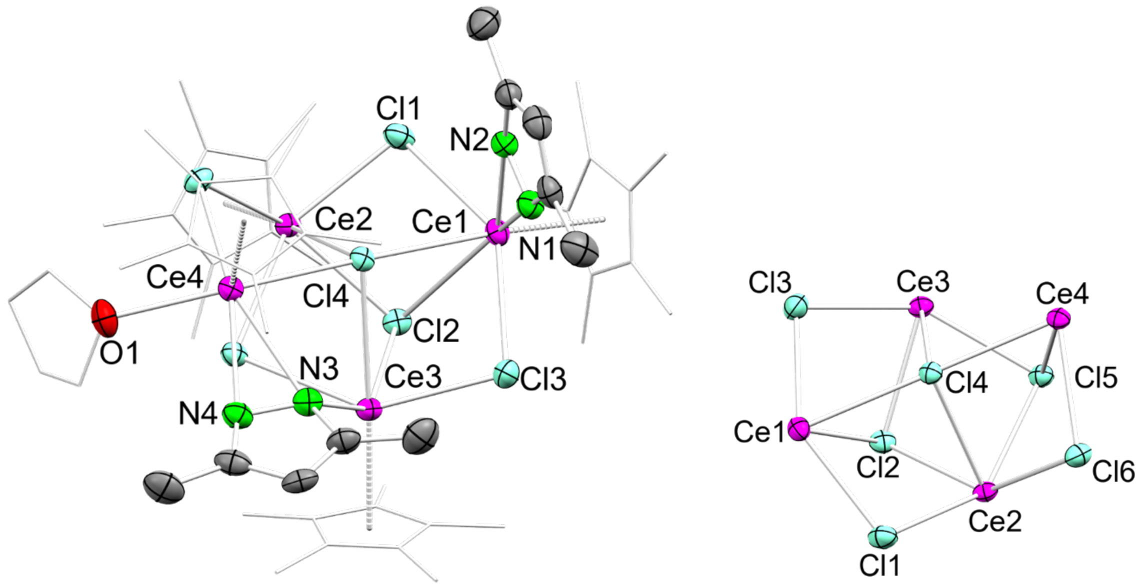

Figure S16. Crystal structure of $Cp*_4Ce_4Cl_6(pz^{Me,Me})_2(thf)$ (**10**) with Ce_4Cl_6 cluster core. Ellipsoids are shown at a 50% probability level. Hydrogen atoms, disordered Cp* and lattice *n*-hexane molecules are omitted for clarity. A part of the molecule is displayed as a wireframe model for improved visibility. Selected interatomic distances [Å]: Ce–Ct 2.455–2.480, Ce1–N1 2.403(5), Ce1–N2 2.428(5), Ce3–N3 2.632(5), Ce4–N3 2.665(5), Ce4–N4 2.504(5), Ce4–O1 2.531(5), Ce1–Cl1 2.9019(16), Ce1–Cl2 3.1121(15), Ce1–Cl3 2.9104(16), Ce1–Cl4 3.0138(14), Ce2–Cl1 2.7529(16), Ce2–Cl2 2.8695(15), Ce2–Cl4 3.0291(13), Ce2–Cl6 2.7831(15), Ce3–Cl3 2.7674(16), Ce3–Cl4 3.0079(3), Ce3–Cl5 2.9028(3), Ce4–Cl4 2.9164(14), Ce4–Cl5 3.0373(14), Ce4–Cl6 2.8958(15). Selected angles [°]: Ce3–N3–Ce4 101.70(17), Ce1–Cl1–Ce2 96.64(5), Ce1–Cl2–Ce2 89.77(4), Ce1–Cl2–Ce3 90.12(4), Ce1–Cl3–Ce3 97.92(5), Ce1–Cl4–Ce2 88.71(4), Ce1–Cl4–Ce3 90.69(4), Ce1–Cl4–Ce4 178.35(4), Ce2–Cl2–Ce3 95.56(4), Ce2–Cl4–Ce3 90.84(4), Ce2–Cl4–Ce4 90.68(4), Ce2–Cl5–Ce3 94.84(4), Ce2–Cl5–Ce4 90.13(4), Ce2–Cl6–Ce4 96.27(4), Ce3–Cl4–Ce4 87.79(4), Ce3–Cl5–Ce4 87.48(4).

NMR Spectra (solvent signals are marked with *, impurities with #)

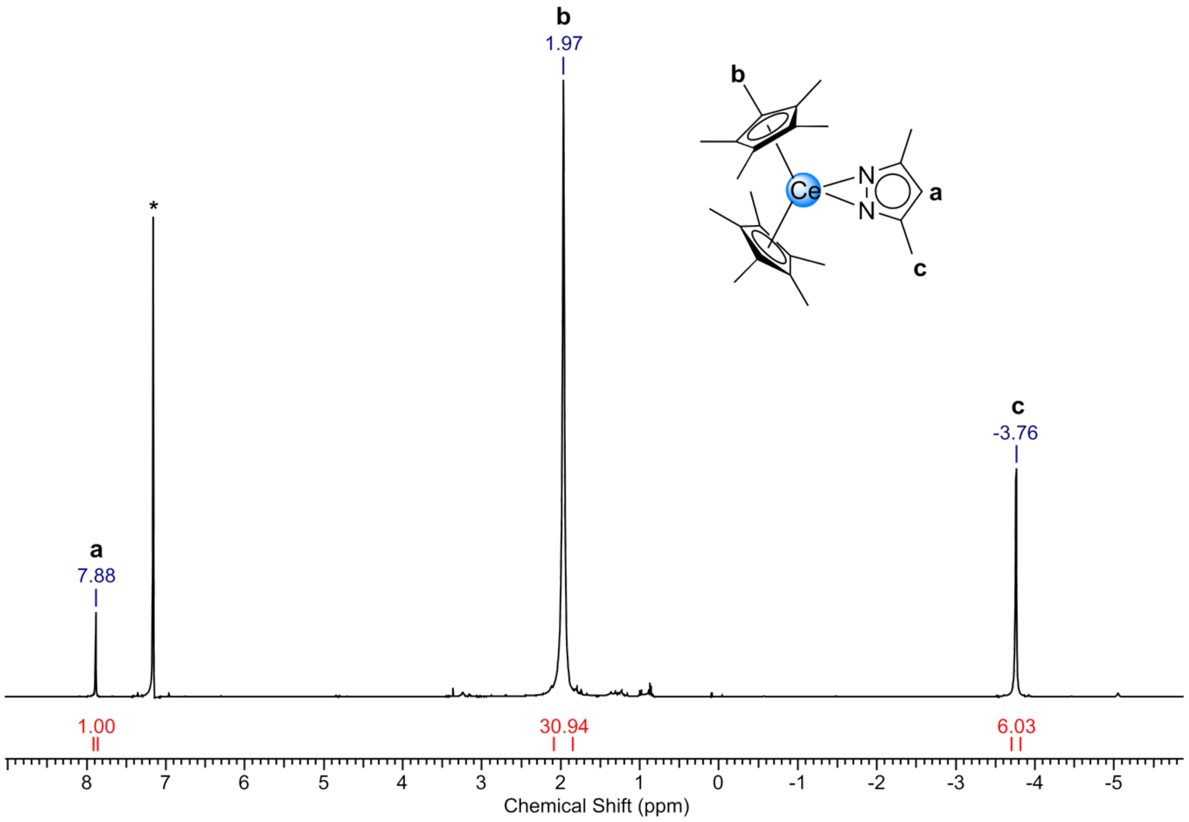

Figure S17. ^{1}H NMR spectrum (C_6D_6, 400.1 MHz, 26 °C) of $Cp*_2Ce(pz^{Me,Me})$ (**1**).

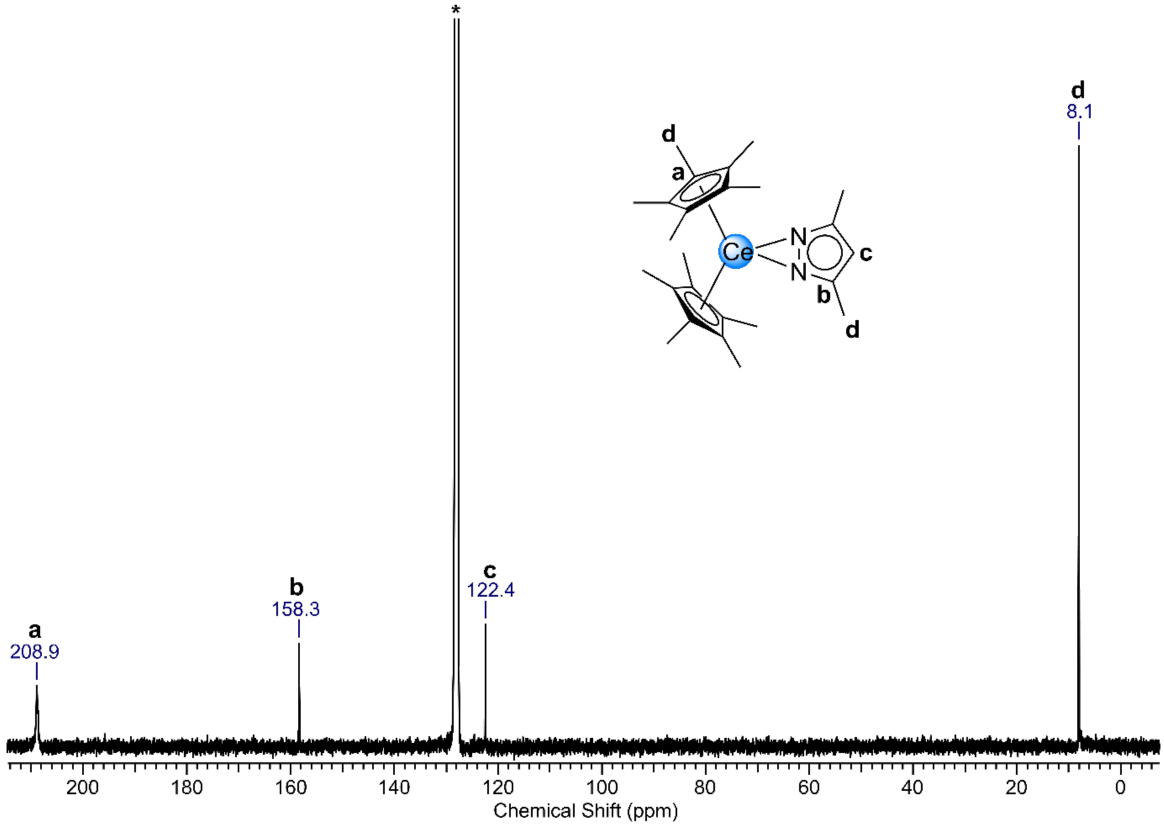

Figure S18. $^{13}C\{^{1}H\}$ NMR spectrum (C_6D_6, 100.6 MHz, 26 °C) of $Cp*_2Ce(pz^{Me,Me})$ (**1**).

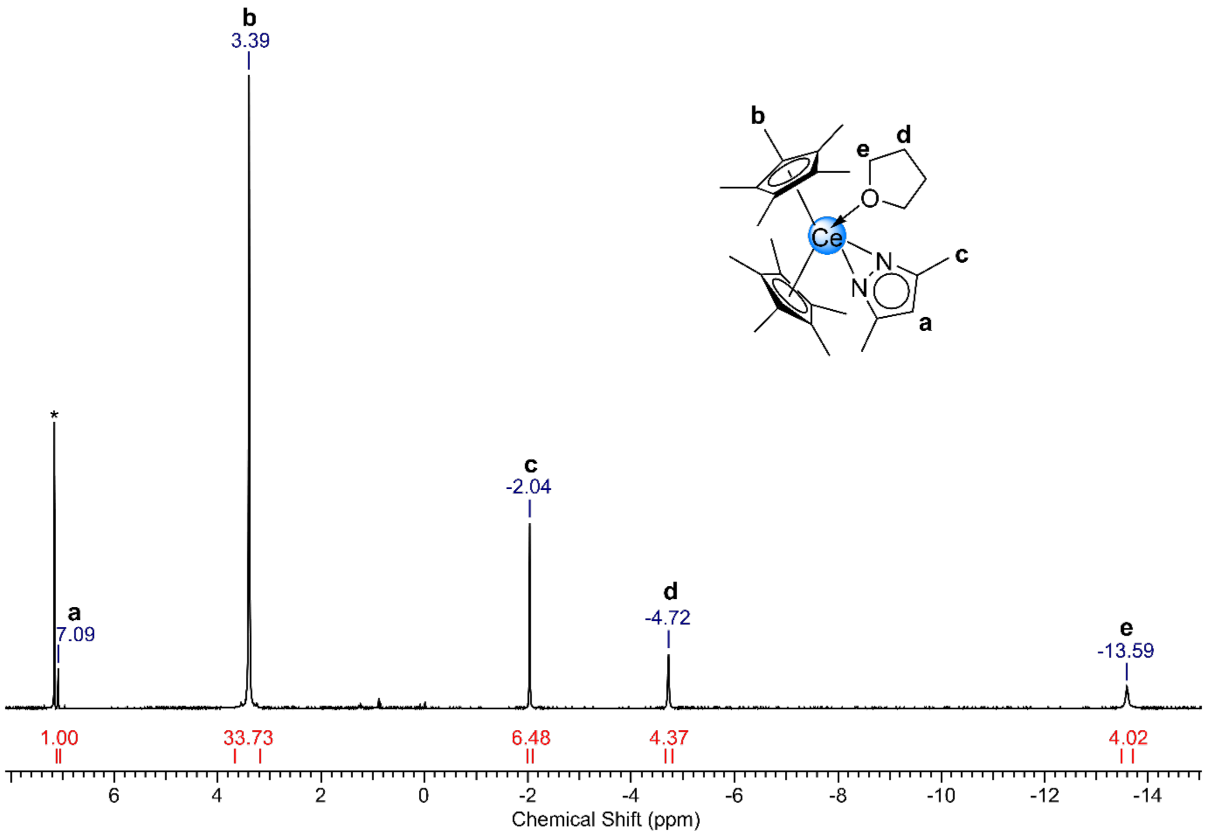

Figure S19. ^{1}H NMR spectrum (C_6D_6, 400.1 MHz, 26 °C) of Cp*$_2$Ce(pz^{Me,Me})(thf) ($\mathbf{1}^{\mathbf{thf}}$).

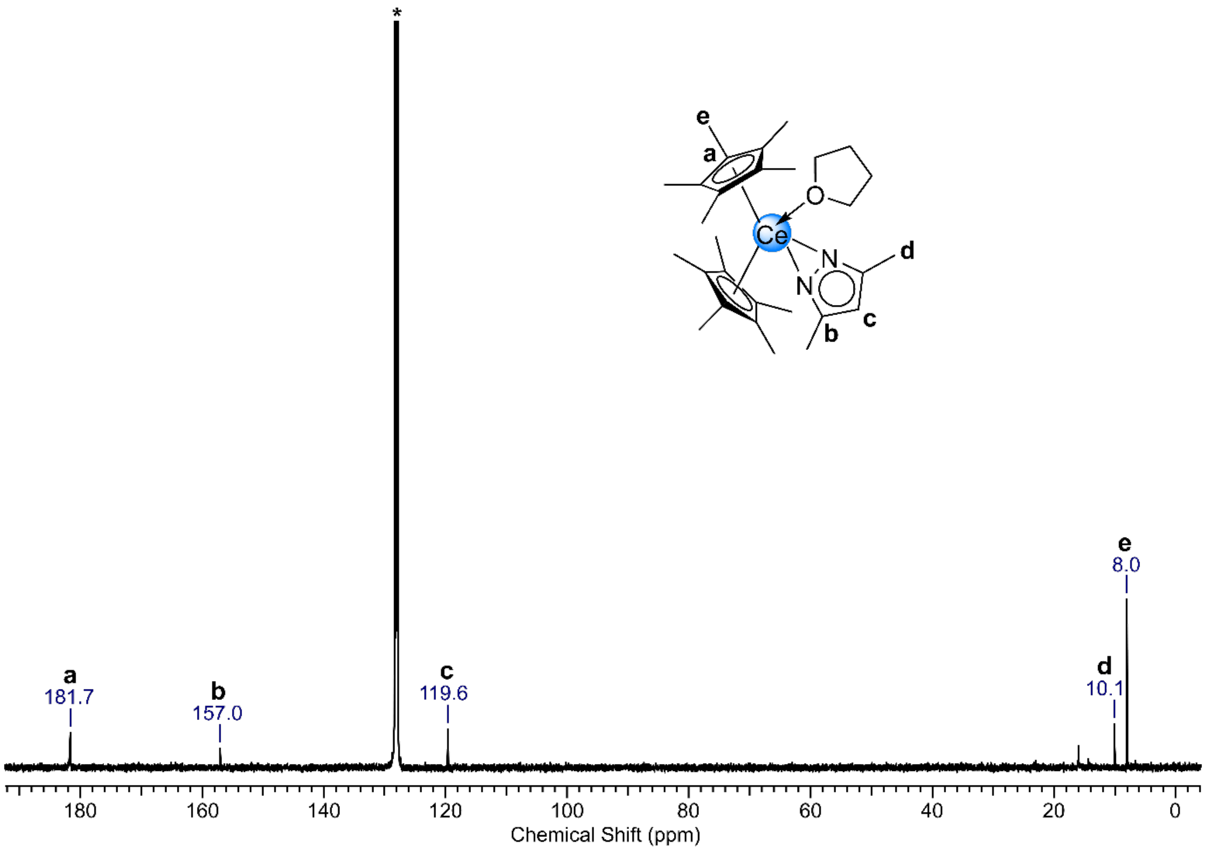

Figure S20. $^{13}C\{^{1}H\}$ NMR spectrum (C_6D_6, 100.6 MHz, 26 °C) of Cp*$_2$Ce(pz^{Me,Me})(thf) ($\mathbf{1}^{\mathbf{thf}}$).

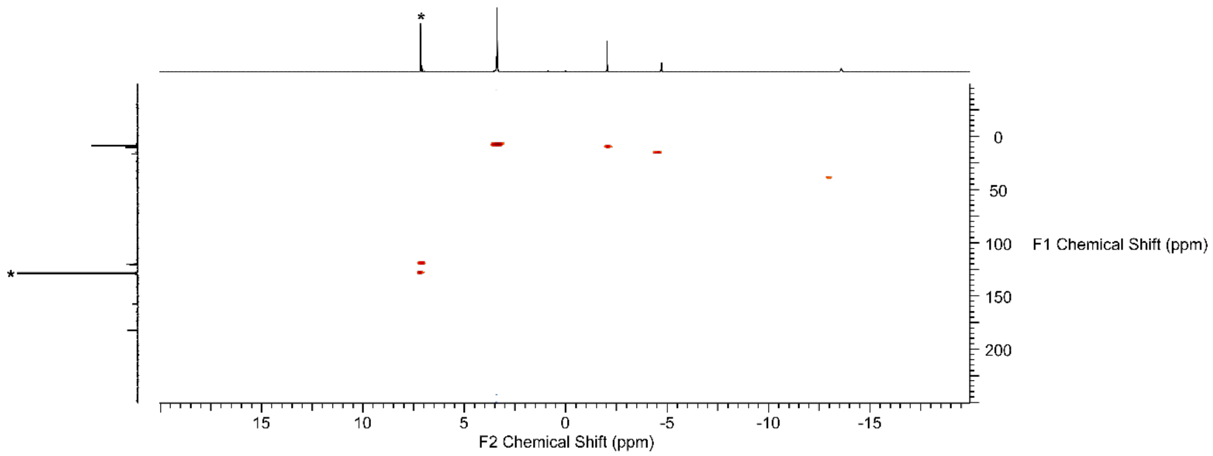

Figure S21. ^{1}H-^{13}C HSQC NMR spectrum (C_6D_6, 400.1 MHz, 100.6 MHz, 26 °C) of $Cp^*{}_2Ce(pz^{Me,Me})(thf)$ (**1**$^{\mathbf{thf}}$).

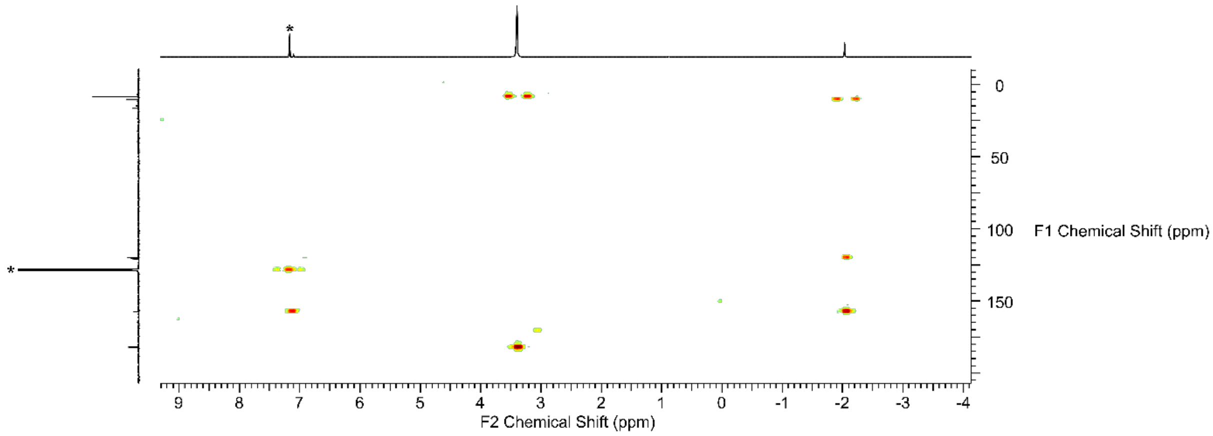

Figure S22. ^{1}H-^{13}C HMBC NMR spectrum (C_6D_6, 400.1 MHz, 100.6 MHz, 26 °C) of $Cp^*{}_2Ce(pz^{Me,Me})(thf)$ (**1**$^{\mathbf{thf}}$).

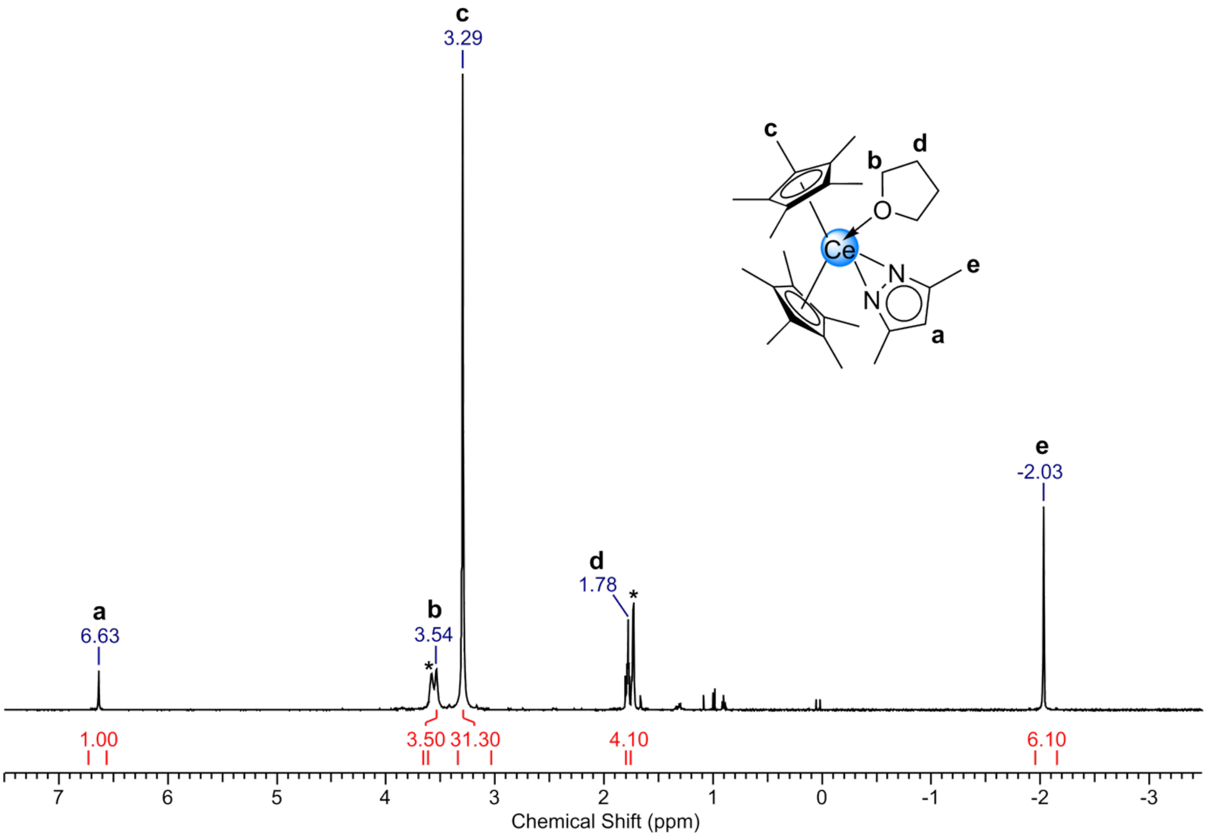

Figure S23. ^{1}H NMR spectrum (THF-d_8, 400.1 MHz, 26 °C) of $Cp^*{}_2Ce(pz^{Me,Me})(thf)$ ($\mathbf{1}^{\mathbf{thf}}$).

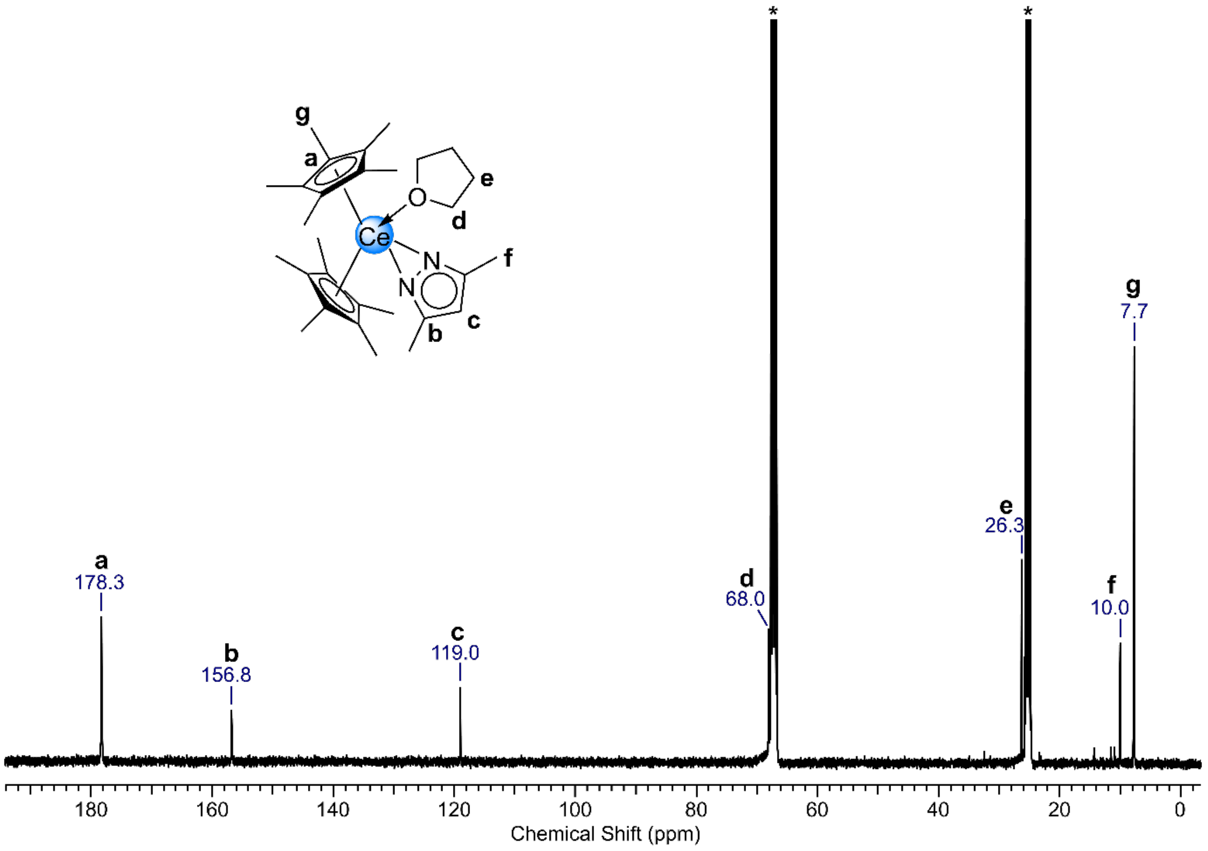

Figure S24. $^{13}C\{^{1}H\}$ NMR spectrum (THF-d_8, 100.6 MHz, 26 °C) of $Cp^*{}_2Ce(pz^{Me,Me})(thf)$ ($\mathbf{1}^{\mathbf{thf}}$).

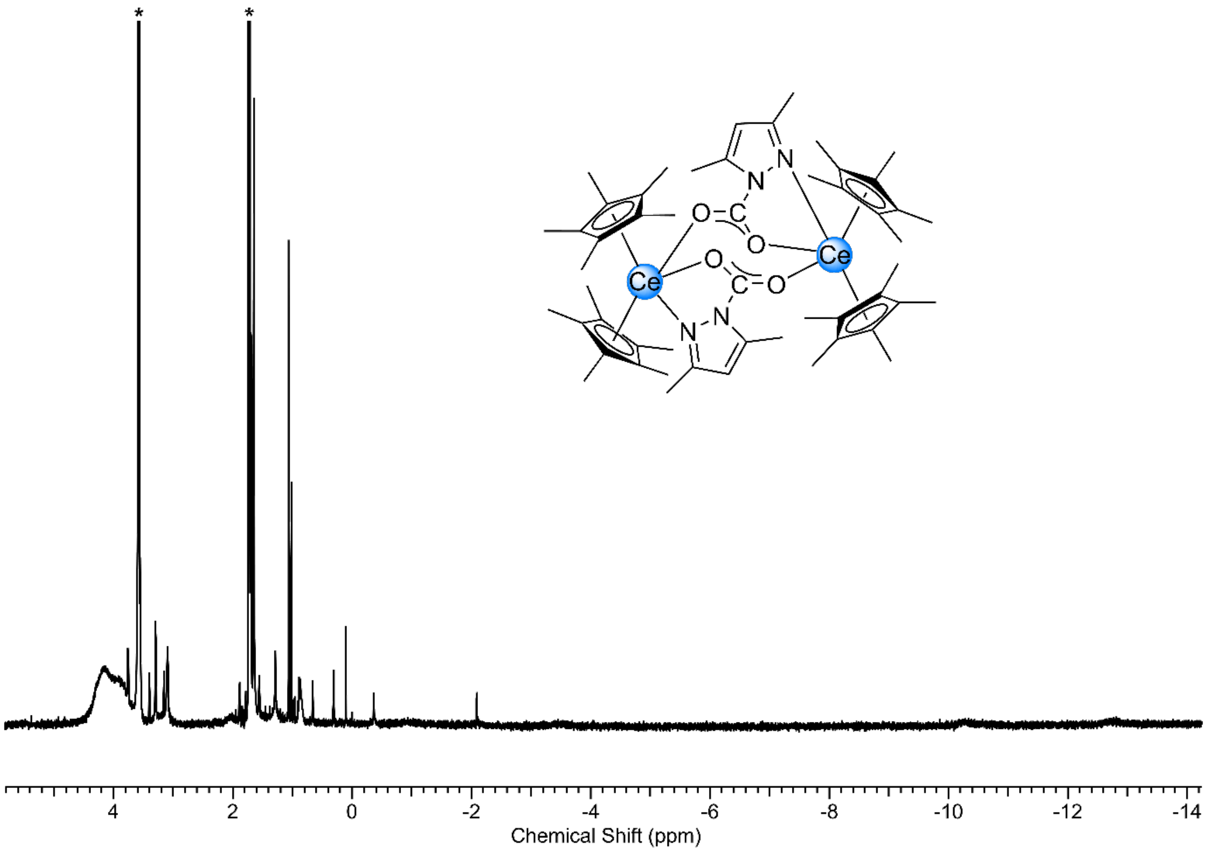

Figure S25. ^{1}H NMR spectrum (THF-d_8, 400.1 MHz, 26 °C) of $[Cp^*_2Ce(pz^{Me,Me}\cdot CO_2)]_2$ (**2**).

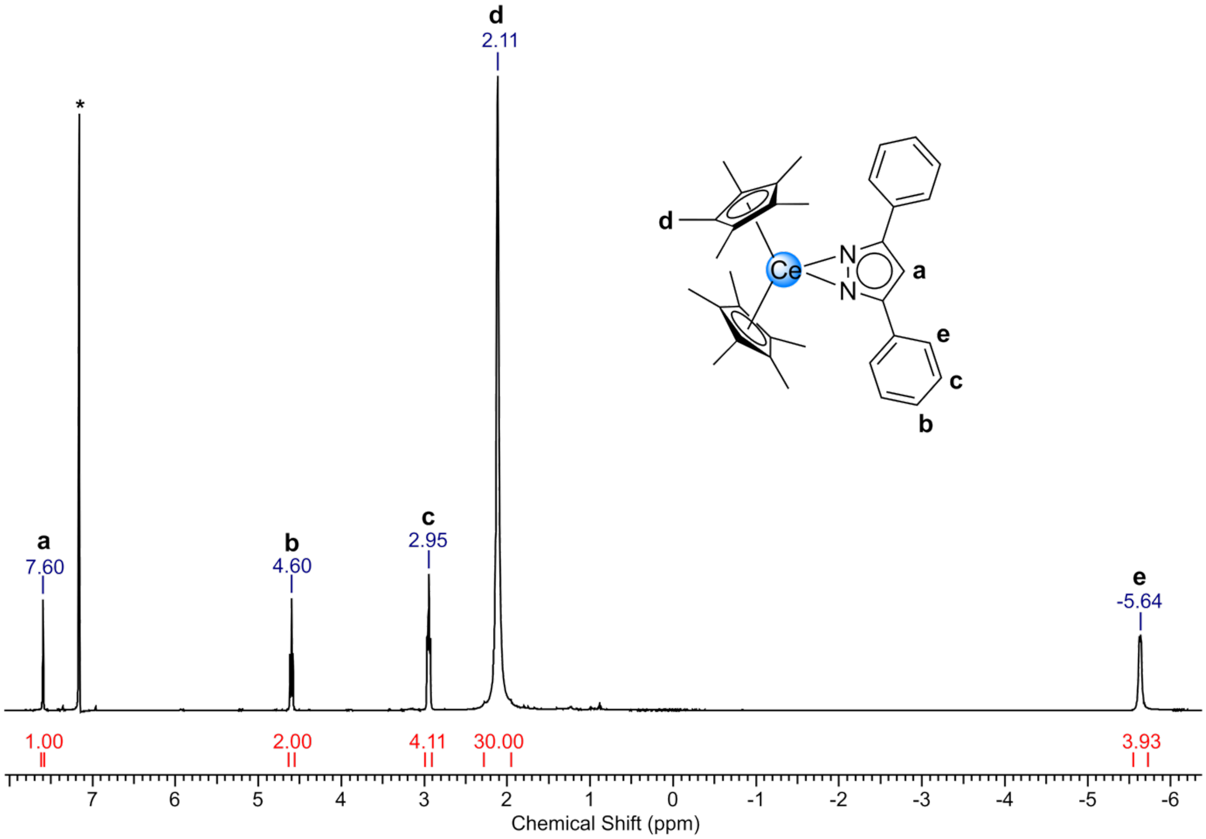

Figure S26. ^{1}H NMR spectrum (C_6D_6, 400.1 MHz, 26 °C) of $Cp^*_2Ce(pz^{Ph,Ph})$ (**3**).

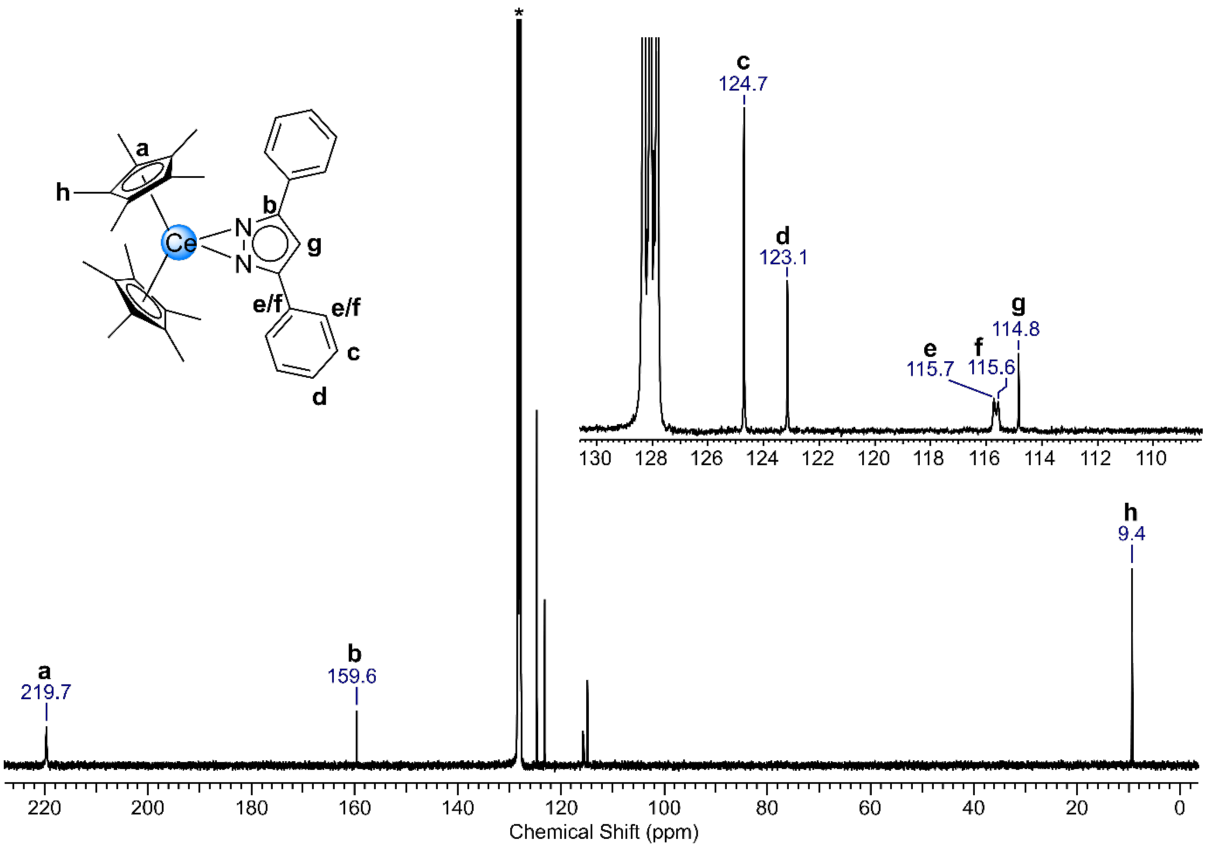

Figure S27. $^{13}C\{^1H\}$ NMR spectrum (C_6D_6, 100.6 MHz, 26 °C) of Cp*$_2$Ce(pz^{Ph,Ph}) (**3**).

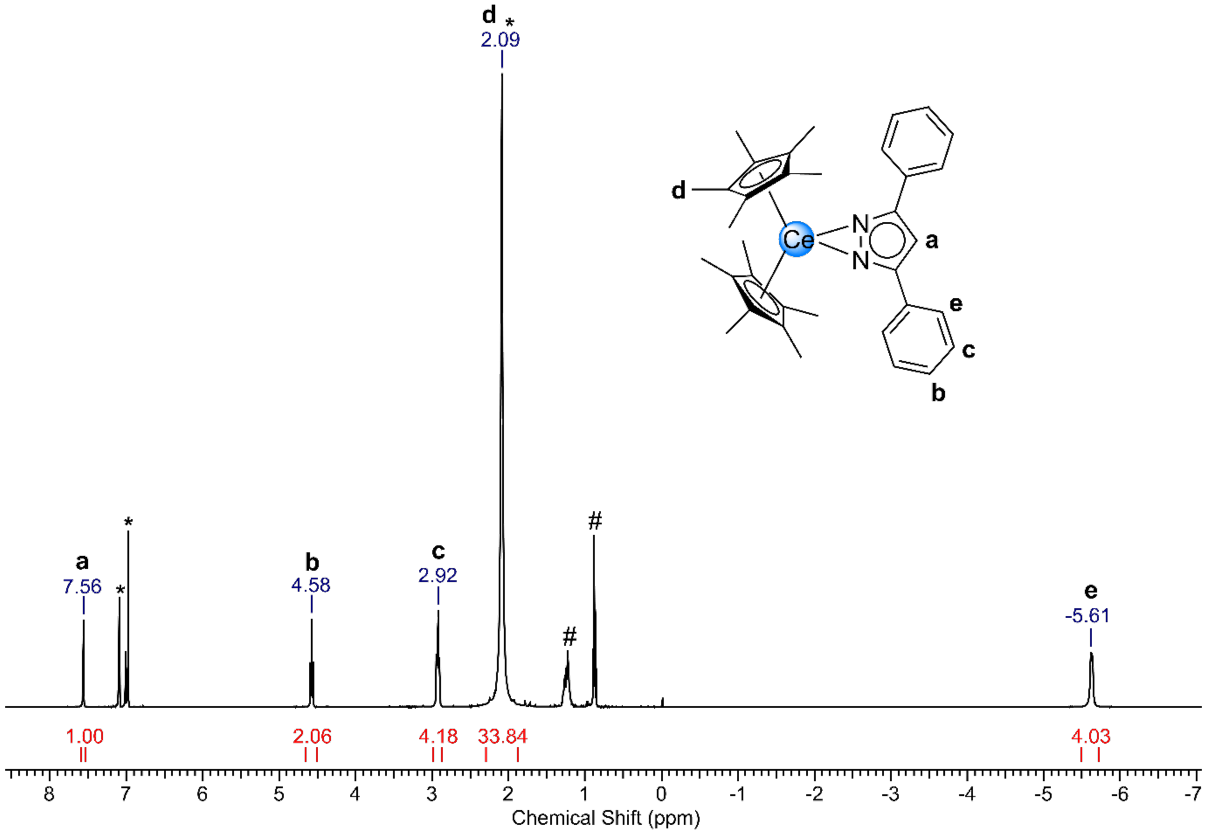

Figure S28. ^1H NMR spectrum (toluene-d_8, 400.1 MHz, 26 °C) of Cp*$_2$Ce(pz^{Ph,Ph}) (**3**).

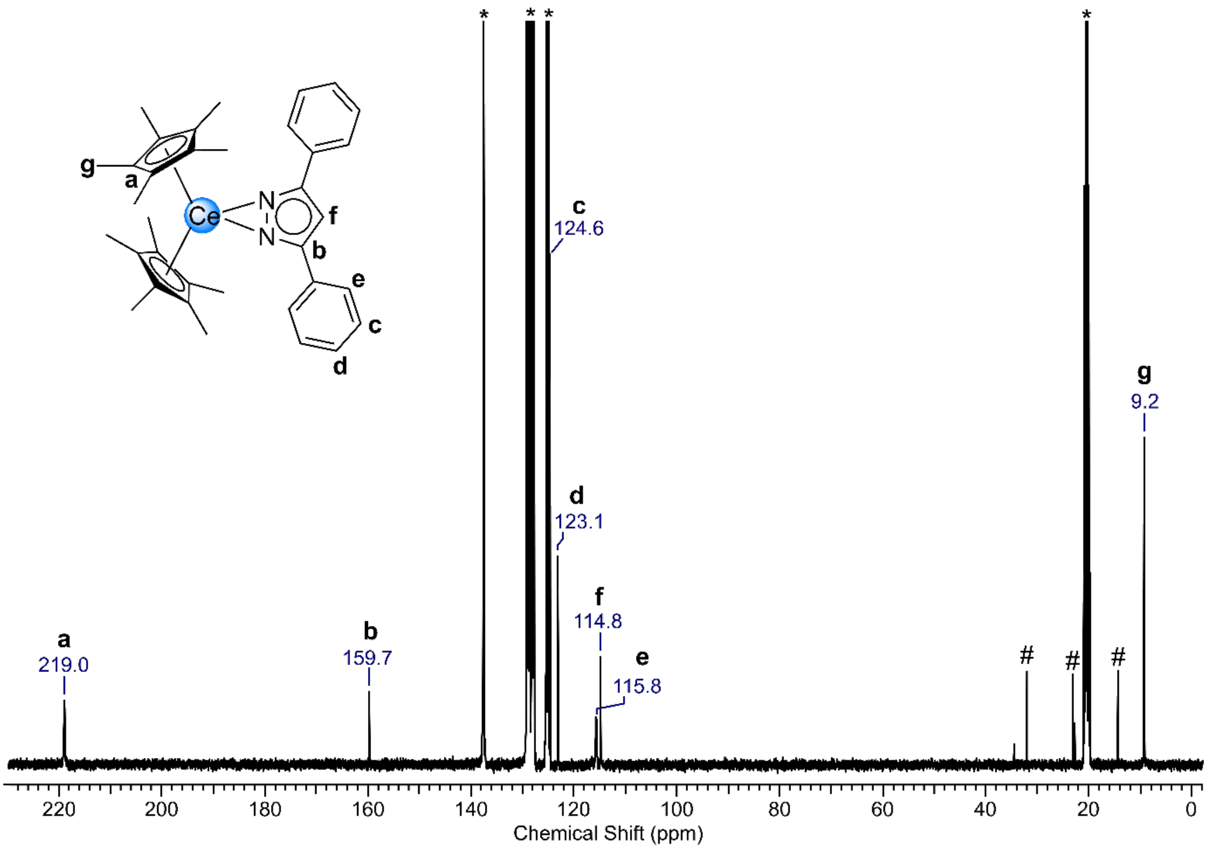

Figure S29. $^{13}C\{^{1}H\}$ NMR spectrum (toluene-d_8, 100.6 MHz, 26 °C) of Cp*$_2$Ce(pz^{Ph,Ph}) (**3**).

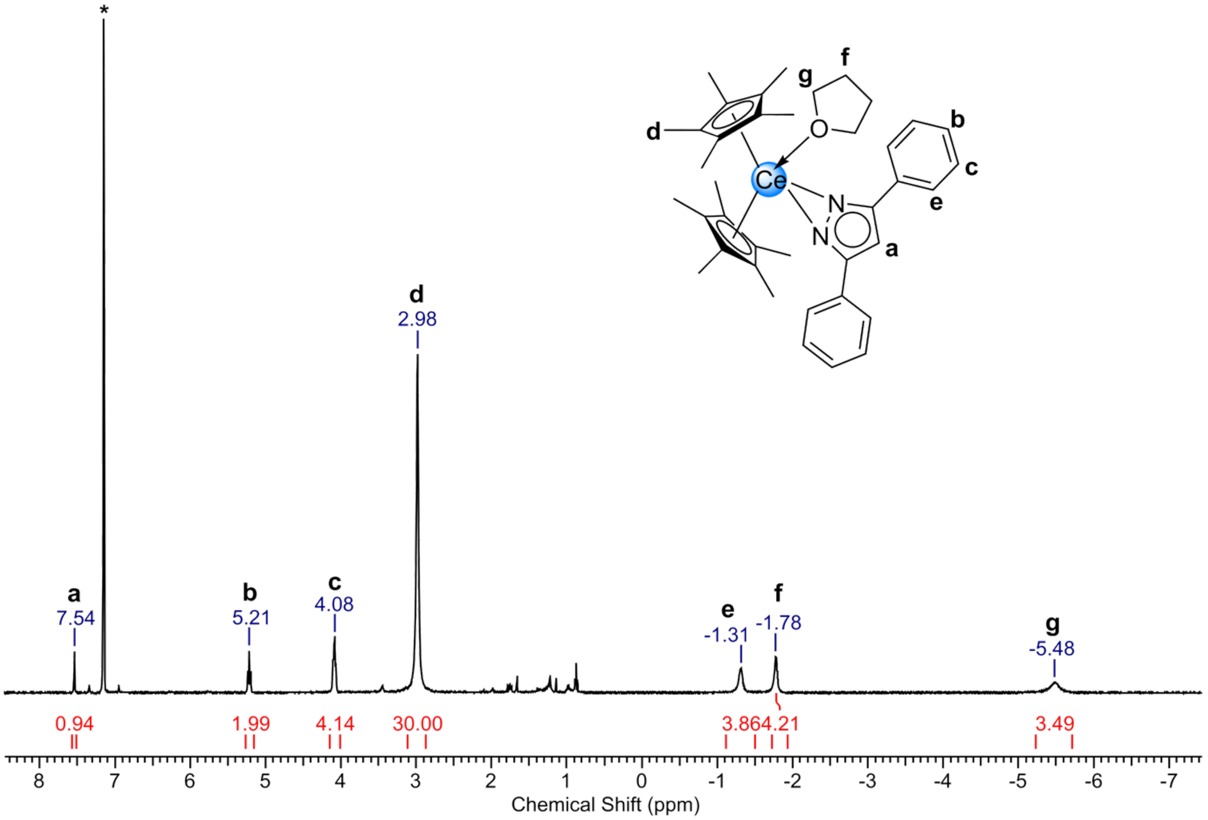

Figure S30. ^{1}H NMR spectrum (C_6D_6, 400.1 MHz, 26 °C) of Cp*$_2$Ce(pz^{Ph,Ph})(thf) (**3^{thf}**).

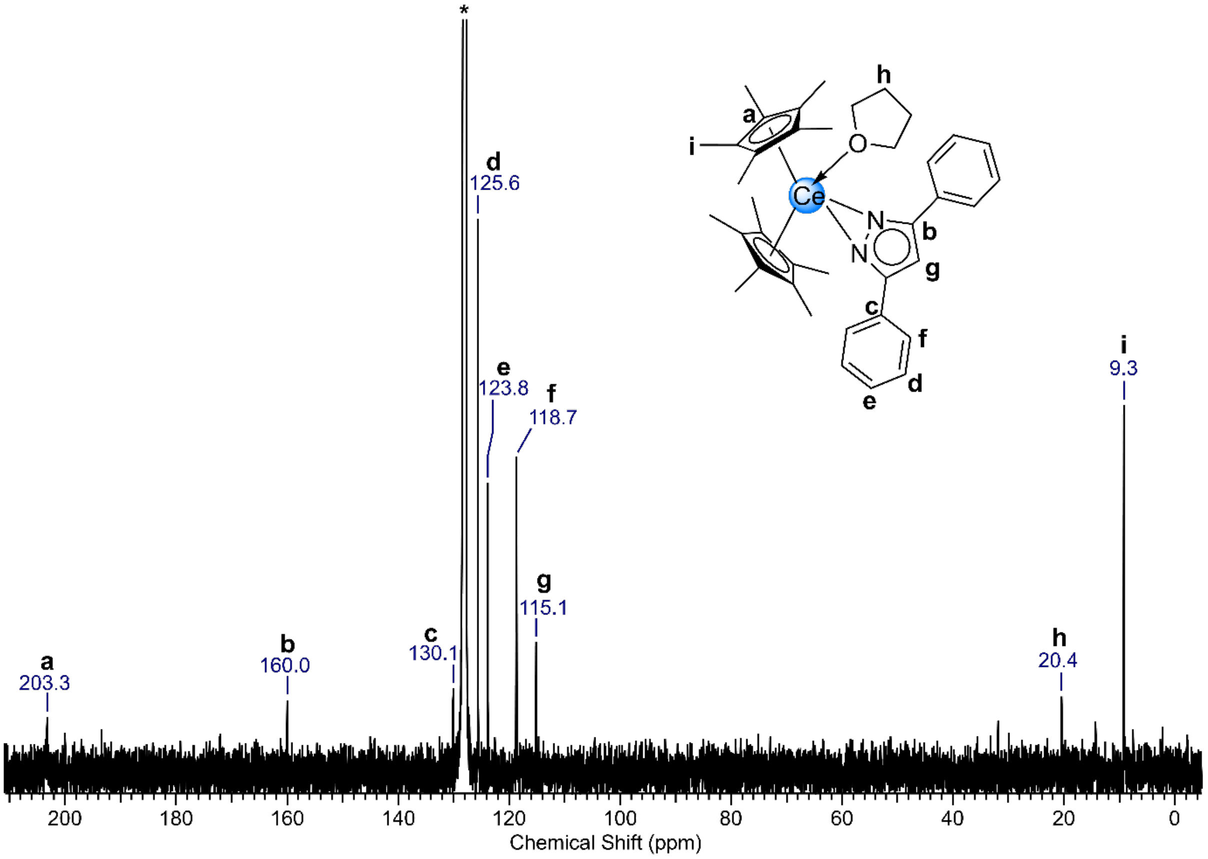

Figure S31. $^{13}C\{^{1}H\}$ NMR spectrum (C_6D_6, 100.6 MHz, 26 °C) of $Cp^*{}_2Ce(pz^{Ph,Ph})(thf)$ (**3^{thf}**).

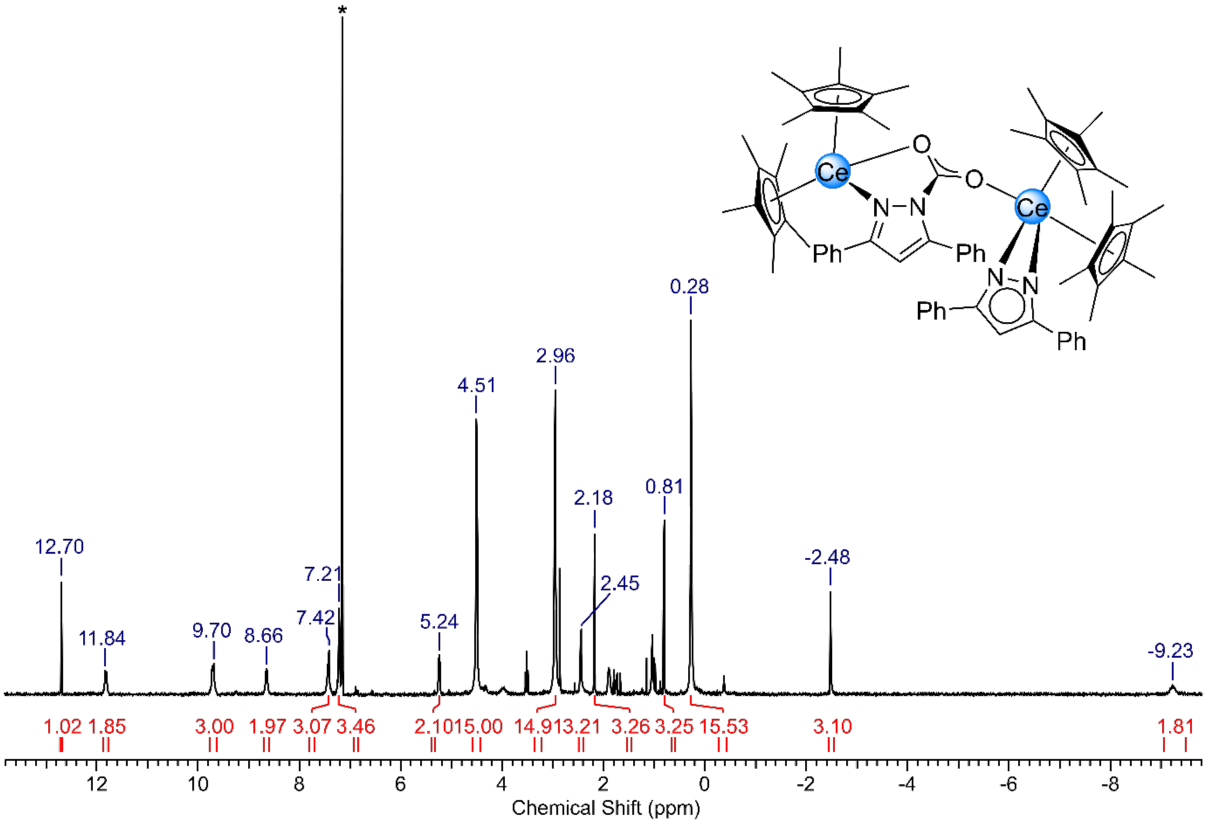

Figure S32. ^{1}H NMR spectrum (C_6D_6, 400.1 MHz, 26 °C) of $Cp^*{}_2Ce(pz^{Ph,Ph}\cdot CO_2)CeCp^*{}_2(pz^{Ph,Ph})$ (**4**).

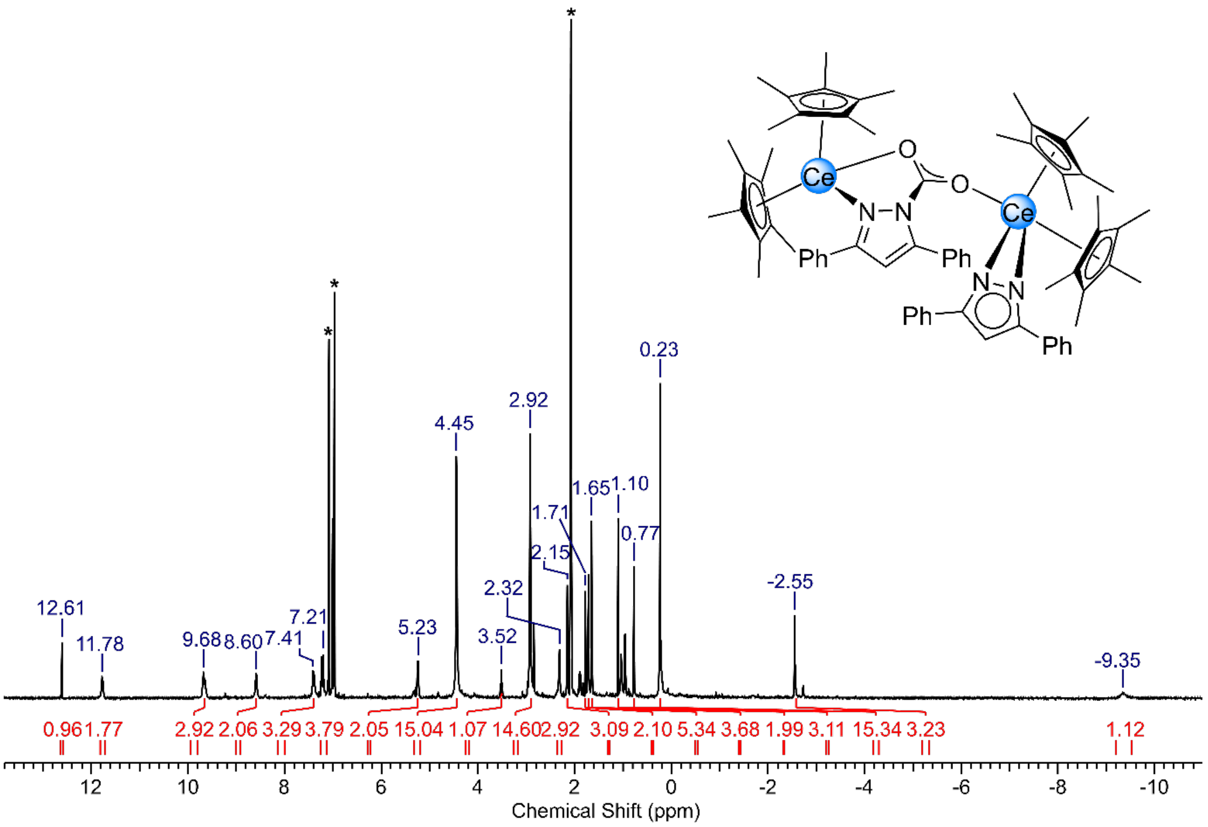

Figure S33. ^{1}H NMR spectrum (toluene-d$_8$, 400.1 MHz, 26 °C) of Cp*$_2$Ce(pz^{Ph,Ph}·CO_2)CeCp*$_2$(pz^{Ph,Ph}) (**4**).

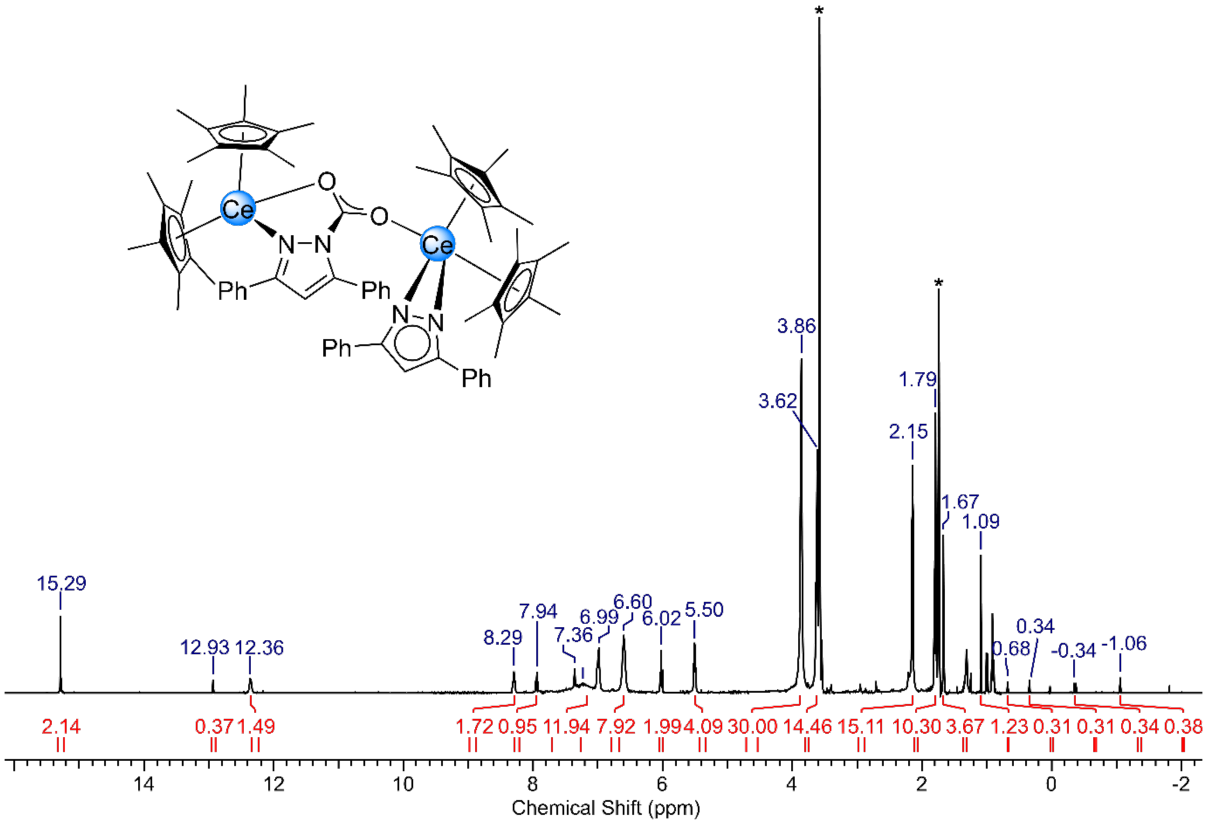

Figure S34. ^{1}H NMR spectrum (THF-d$_8$, 400.1 MHz, 26 °C) of Cp*$_2$Ce(pz^{Ph,Ph}·CO_2)CeCp*$_2$(pz^{Ph,Ph}) (**4**).

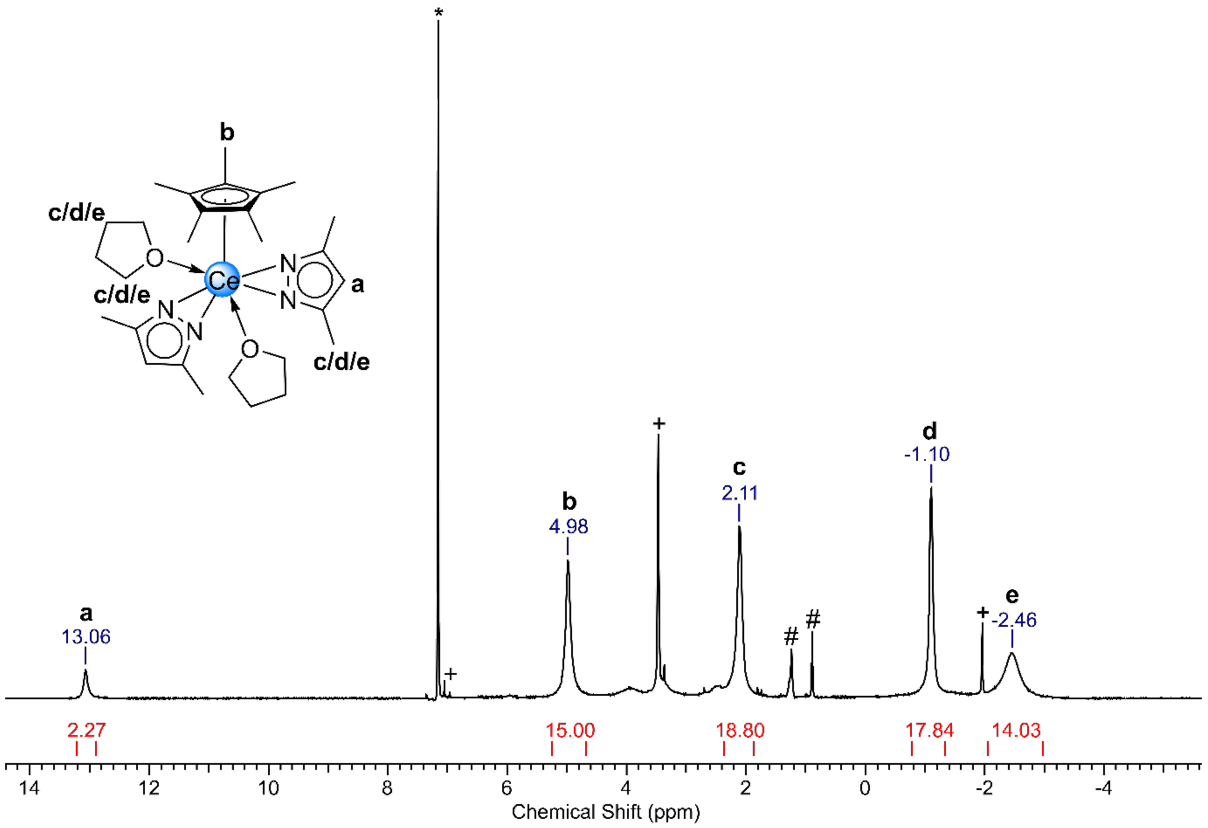

Figure S35. ^{1}H NMR spectrum (C_6D_6, 400.1 MHz, 26 °C) of Cp*Ce(pz^{Me,Me})$_2$(thf)$_2$ (**5**) (+ = Signals of sandwich complex **1^{thf}**).

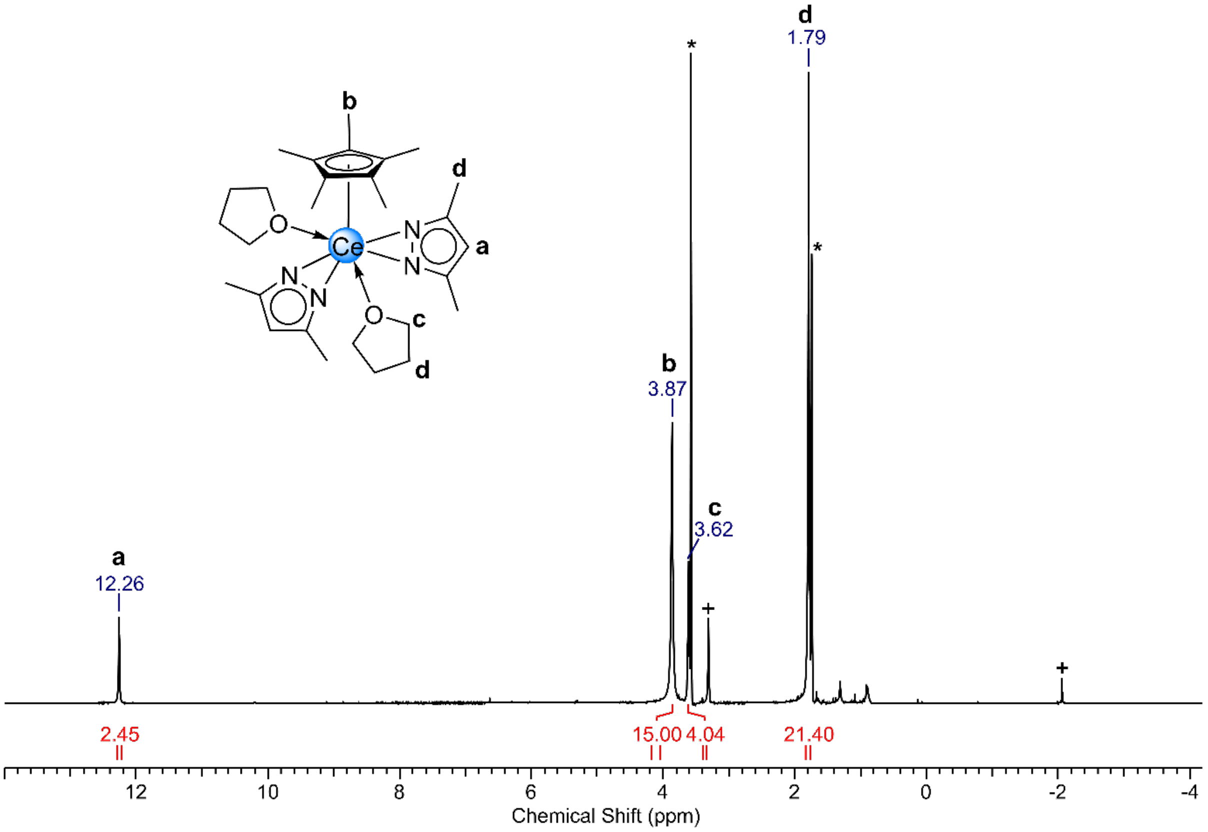

Figure S36. ^{1}H NMR spectrum (THF-d_8, 400.1 MHz, 26 °C) of Cp*Ce(pz^{Me,Me})$_2$(thf)$_2$ (**5**) (+ = Signals of sandwich complex **1^{thf}**).

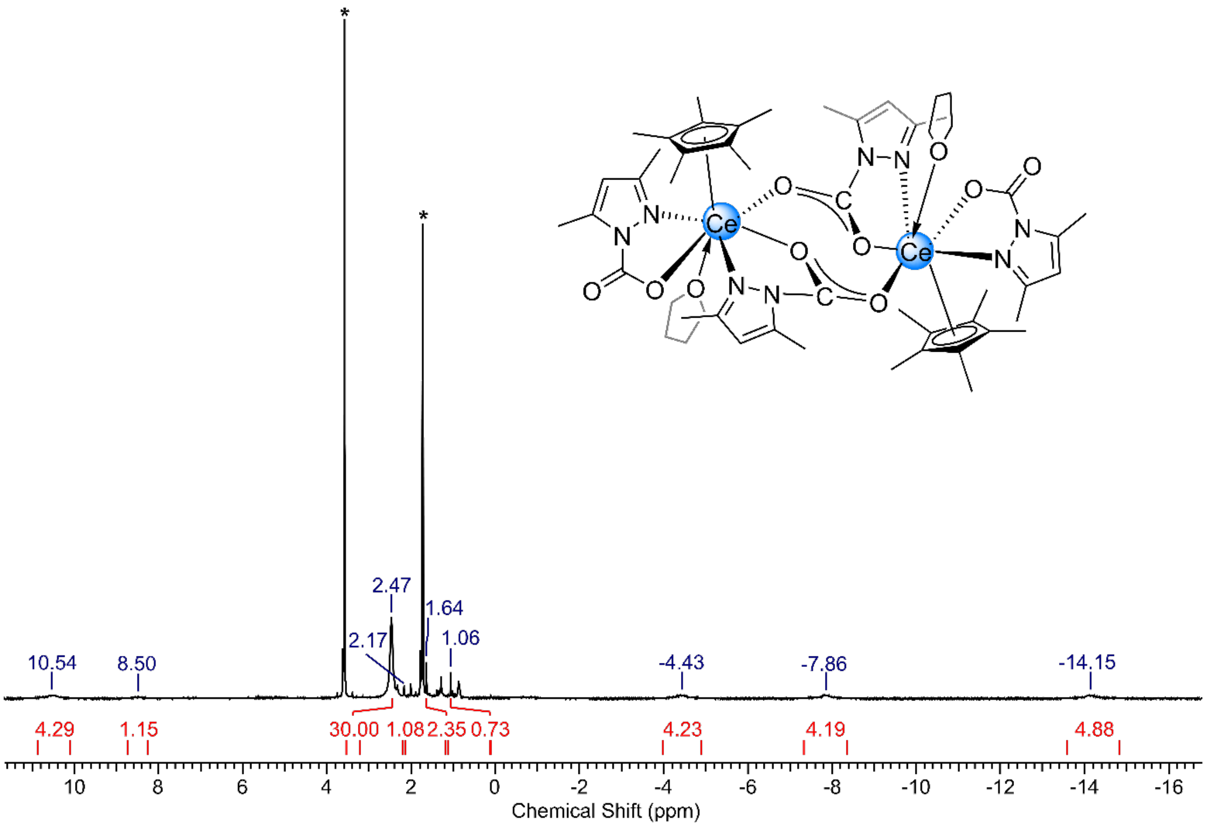

Figure S37. ^{1}H NMR spectrum (THF-d_8, 400.1 MHz, 26 °C) of [Cp*Ce(pz^{Me,Me}·CO_2)$_2$(thf)]$_2$ (**6**).

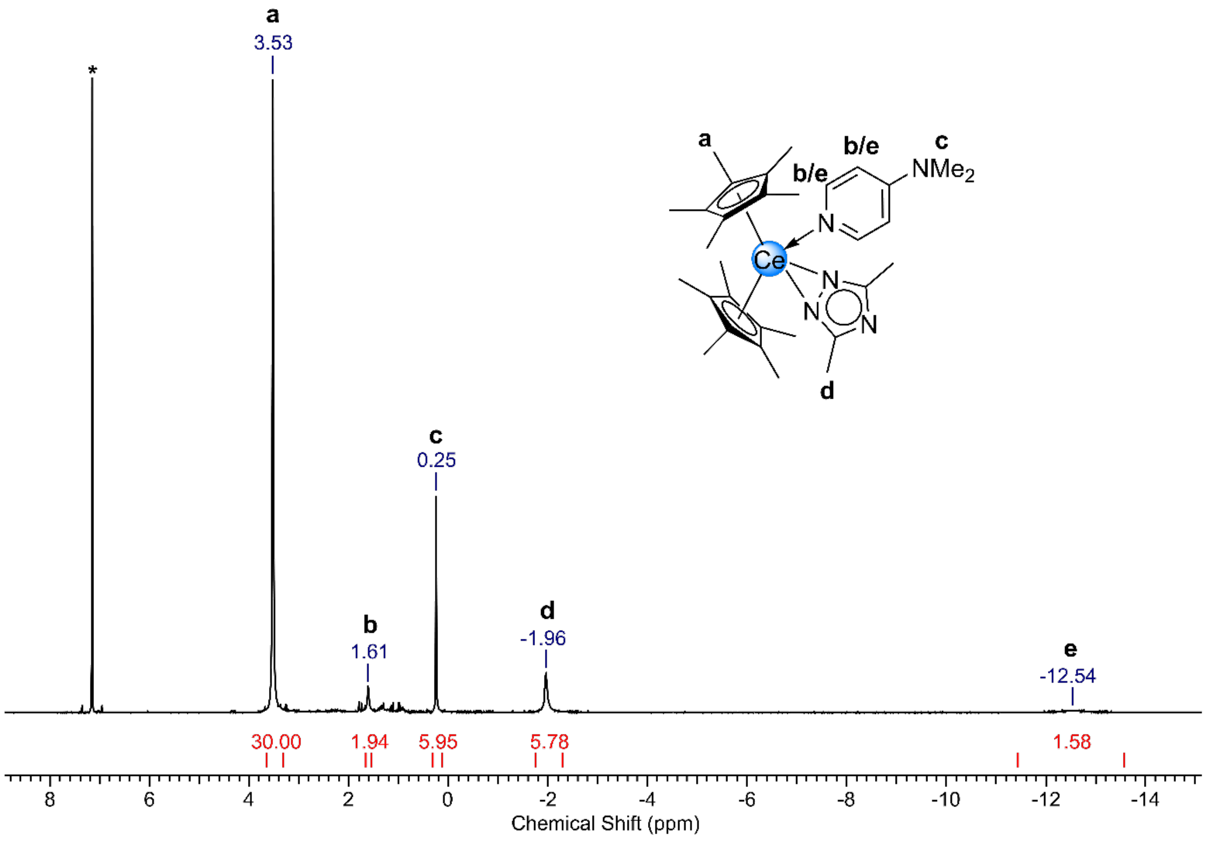

Figure S38. ^{1}H NMR spectrum (C_6D_6, 400.1 MHz, 26 °C) of Cp*$_2$Ce(tz^{Me,Me})(dmap) (**7**).

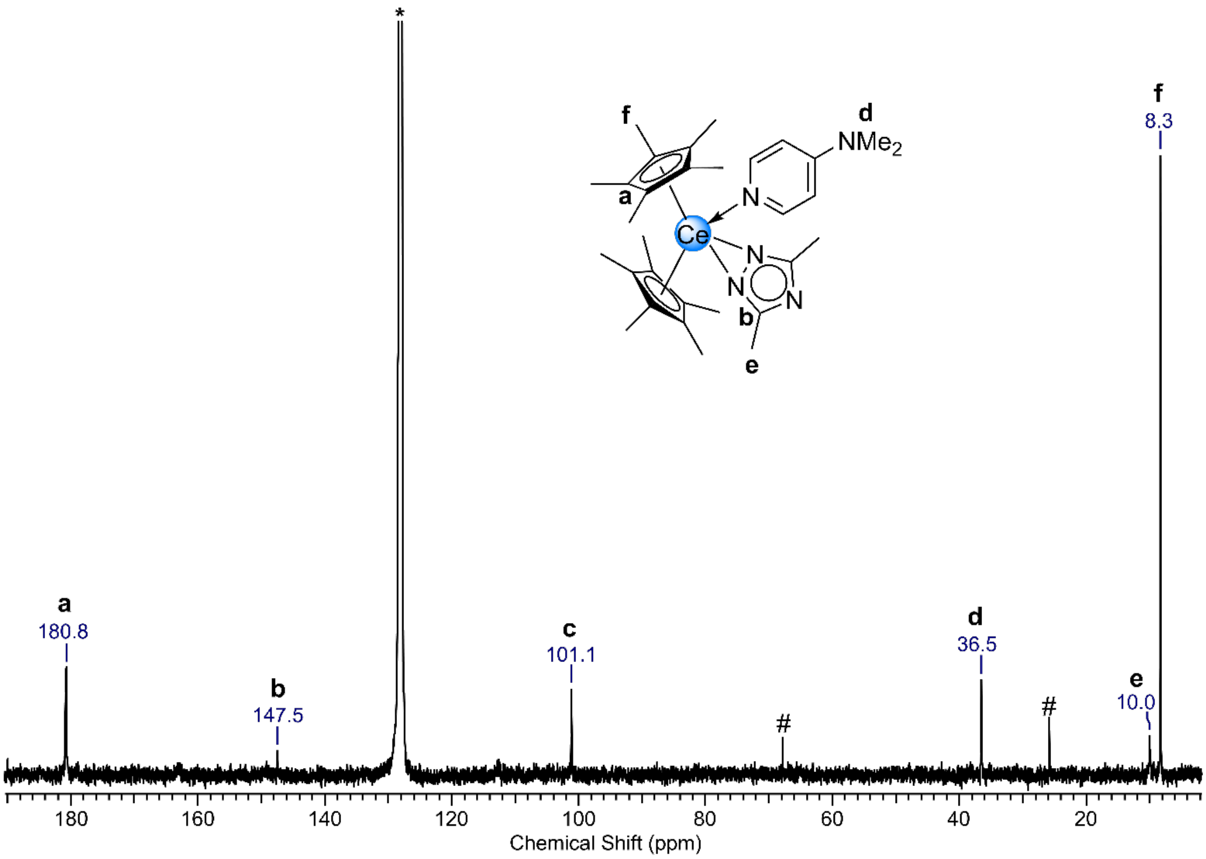

Figure S39. $^{13}C\{^{1}H\}$ NMR spectrum (C_6D_6, 125.8 MHz, 26 °C) of $Cp^*_2Ce(tz^{Me,Me})(dmap)$ (**7**).

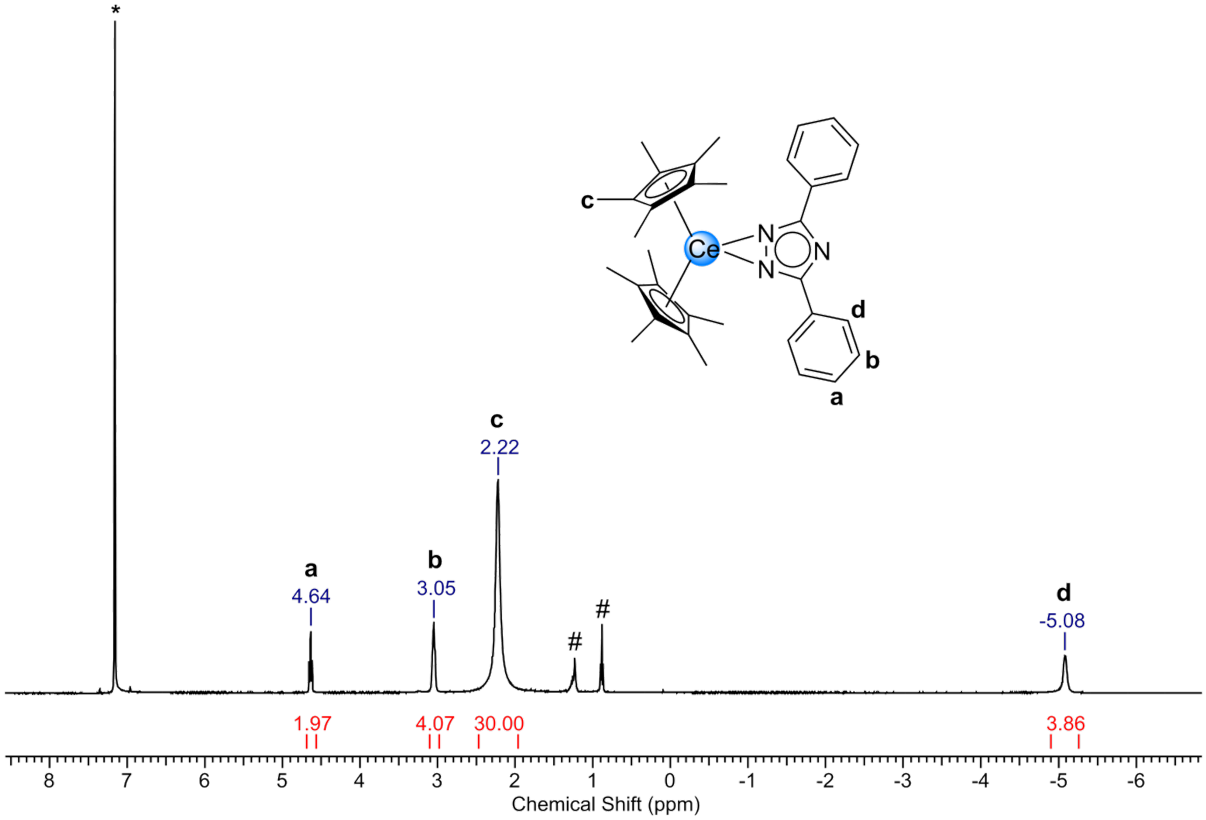

Figure S40. ^{1}H NMR spectrum (C_6D_6, 400.1 MHz, 26 °C) of $Cp^*_2Ce(tz^{Ph,Ph})$ (**8**).

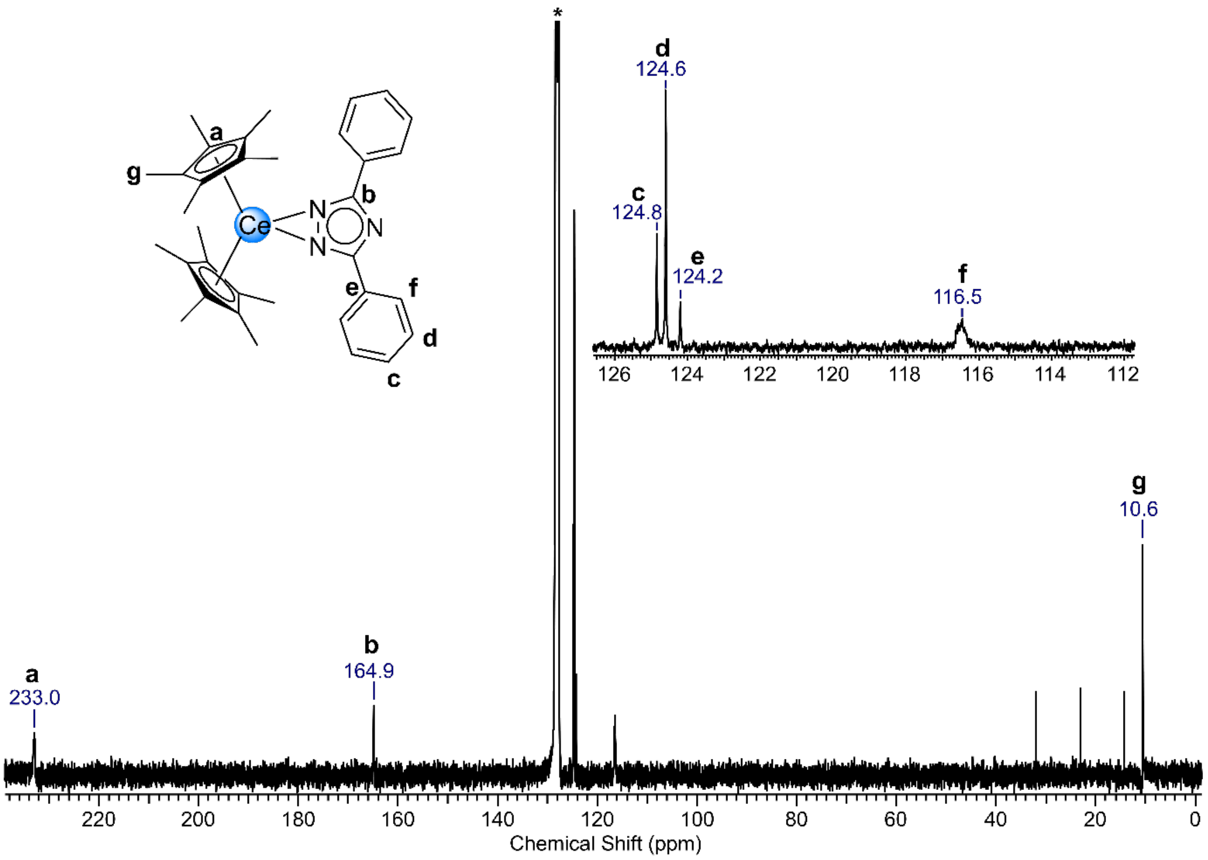

Figure S41. $^{13}C\{^{1}H\}$ NMR spectrum (C_6D_6, 100.6 MHz, 26 °C) of $Cp^*{}_2Ce(tz^{Ph,Ph})$ (**8**).

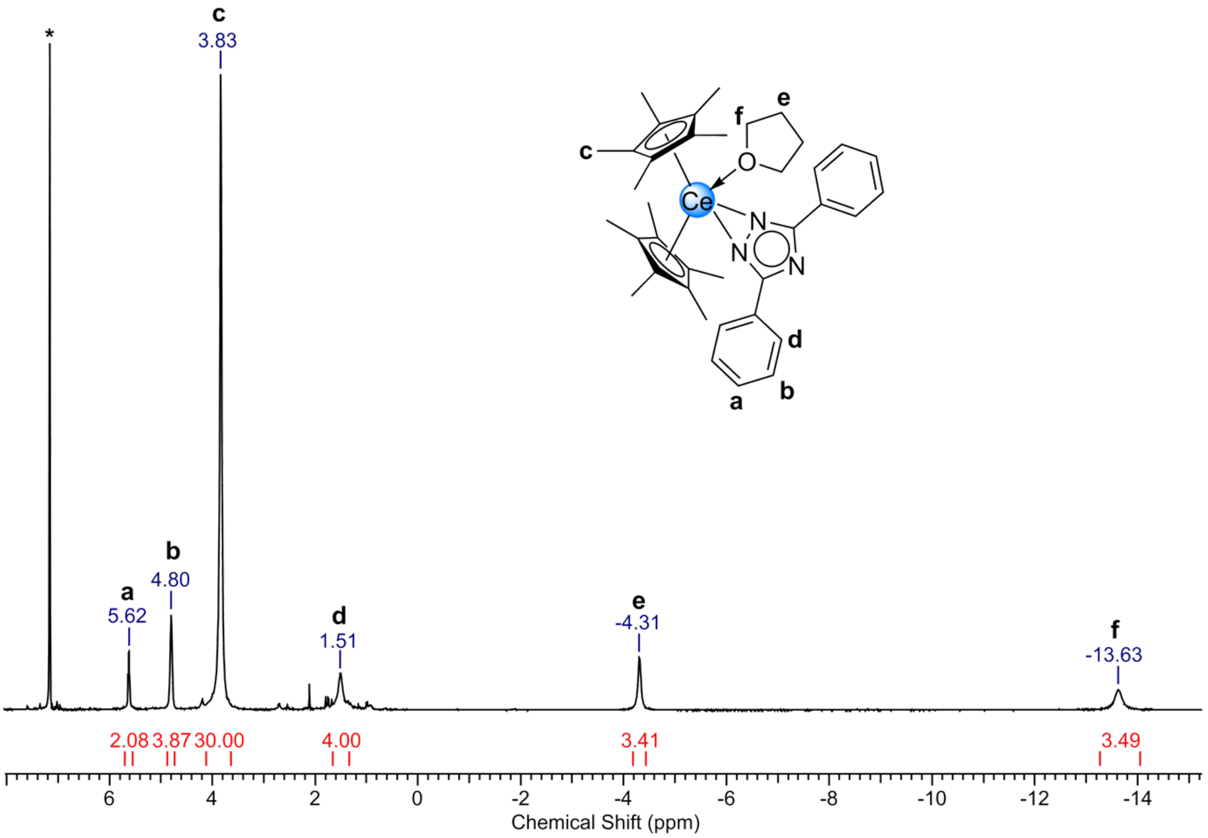

Figure S42. ^{1}H NMR spectrum (C_6D_6, 400.1 MHz, 26 °C) of $Cp^*{}_2Ce(tz^{Ph,Ph})(thf)$ (**8^{thf}**).

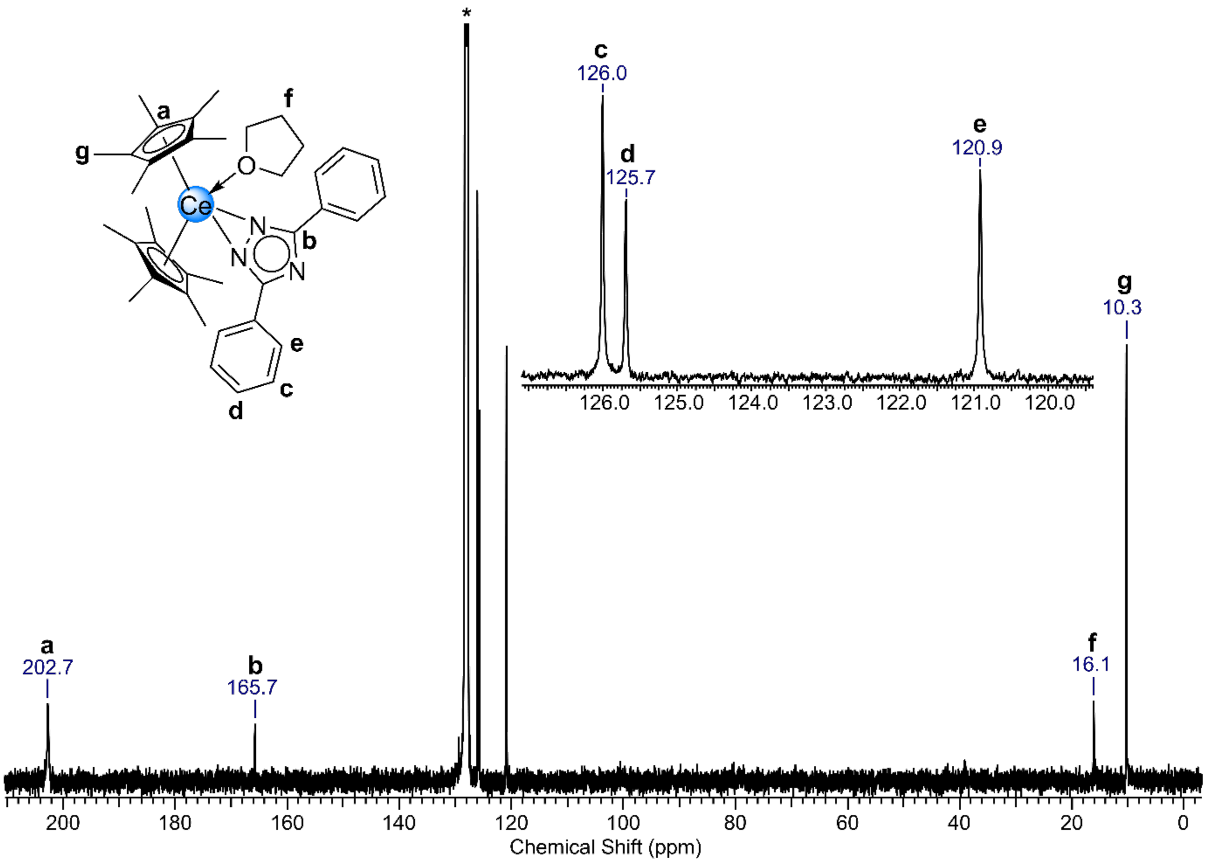

Figure S43. $^{13}C\{^1H\}$ NMR spectrum (C_6D_6, 100.6 MHz, 26 °C) of $Cp^*_2Ce(tz^{Ph,Ph})(thf)$ (**8^{thf}**).

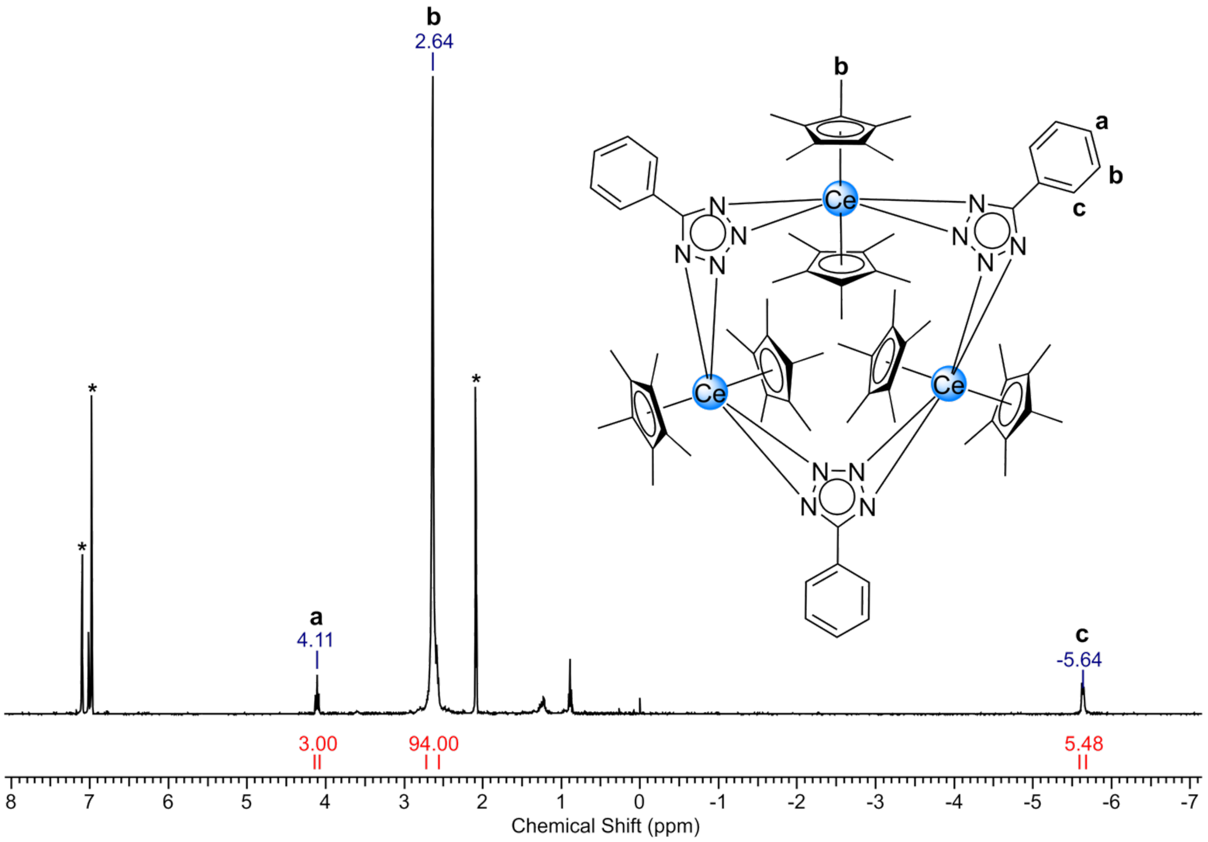

Figure S44. ^1H NMR spectrum (toluene-d_8, 400.1 MHz, 26 °C) of $[Cp^*_2Ce(tet^{Ph})]_3$ (**9-Ce**).

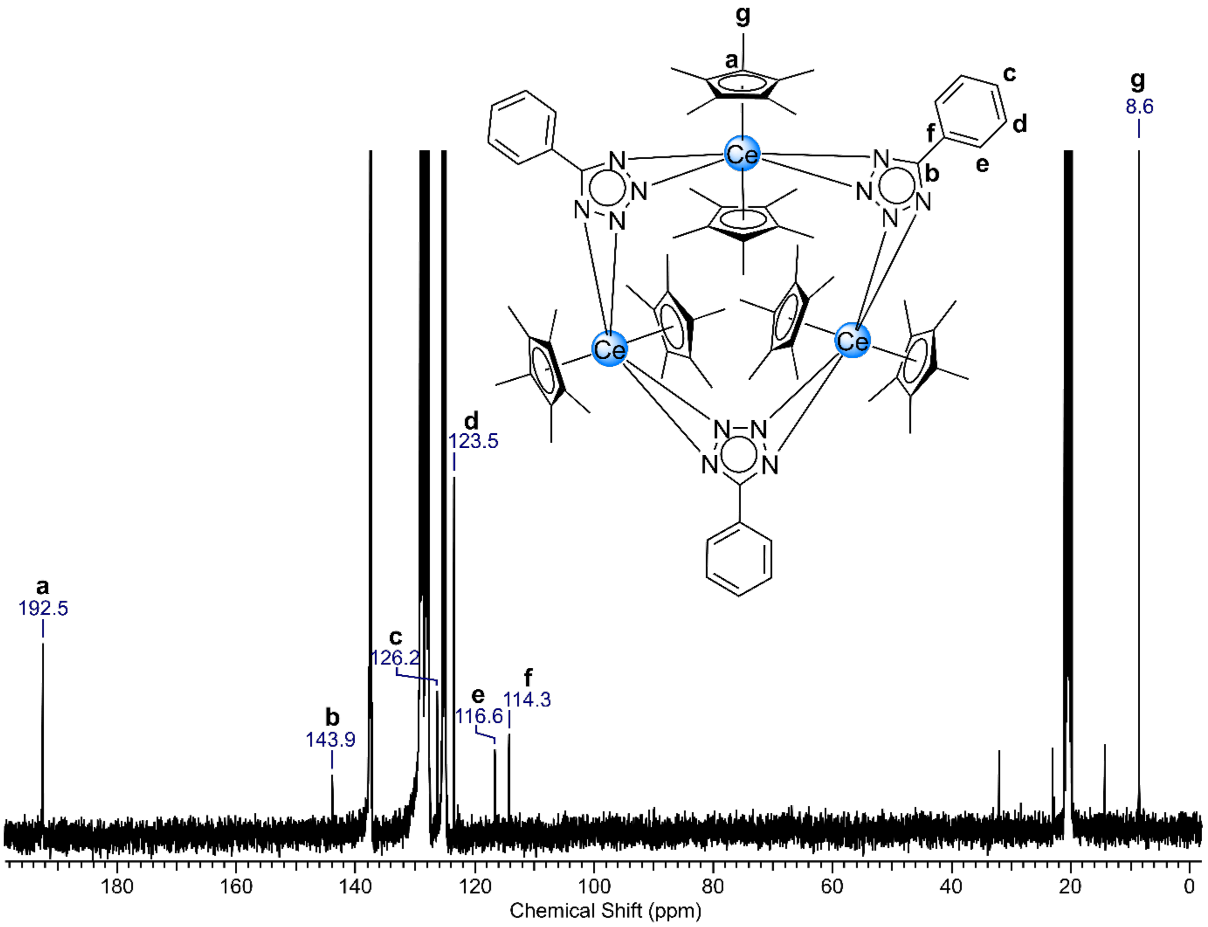

Figure S45. $^{13}C\{^{1}H\}$ NMR spectrum (toluene-d$_8$, 100.6 MHz, 26 °C) of [Cp*$_2$Ce(tet^{Ph})]$_3$ (**9-Ce**).

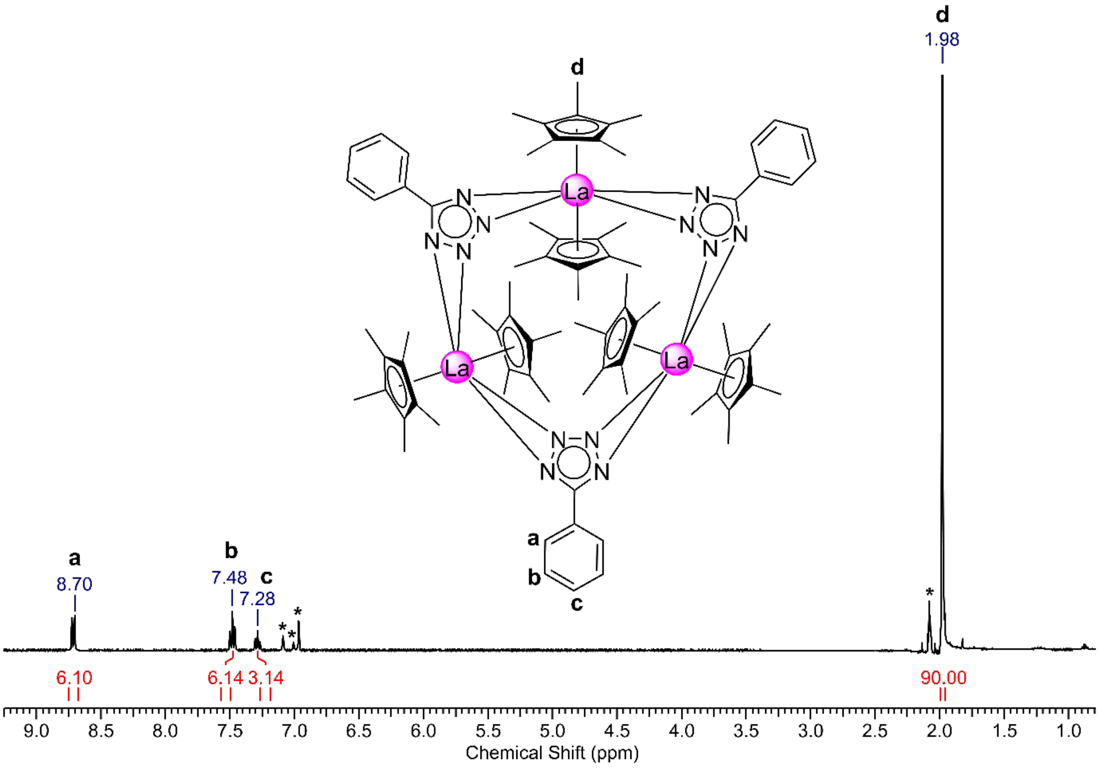

Figure S46. ^{1}H NMR spectrum (toluene-d$_8$, 400.1 MHz, 26 °C) of [Cp*$_2$La(tet^{Ph})]$_3$ (**9-La**).

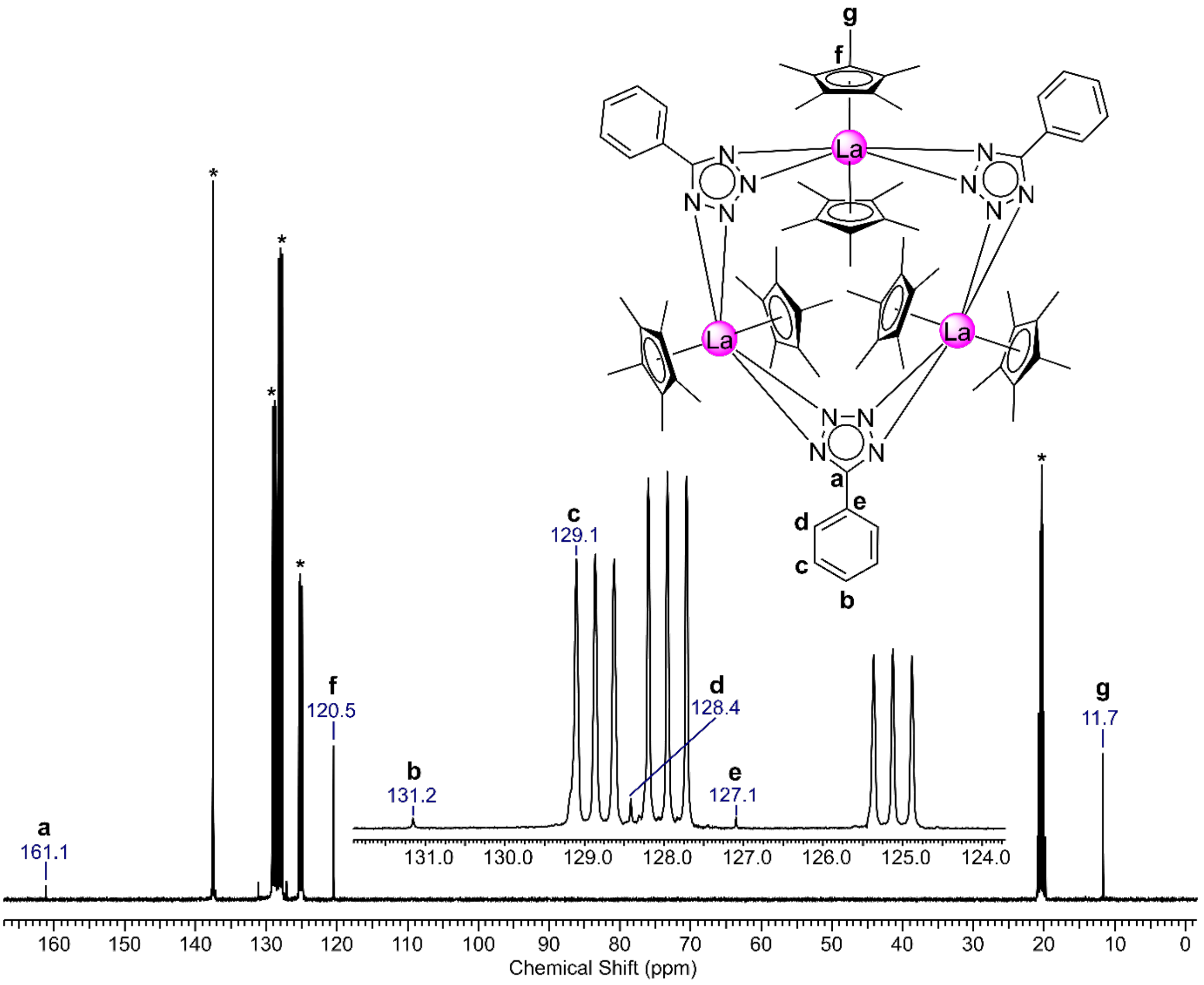

Figure S47. $^{13}C\{^{1}H\}$ NMR spectrum (toluene-d_8, 100.6 MHz, 26 °C) of $[Cp^*_2La(tet^{Ph})]_3$ (**9-La**).

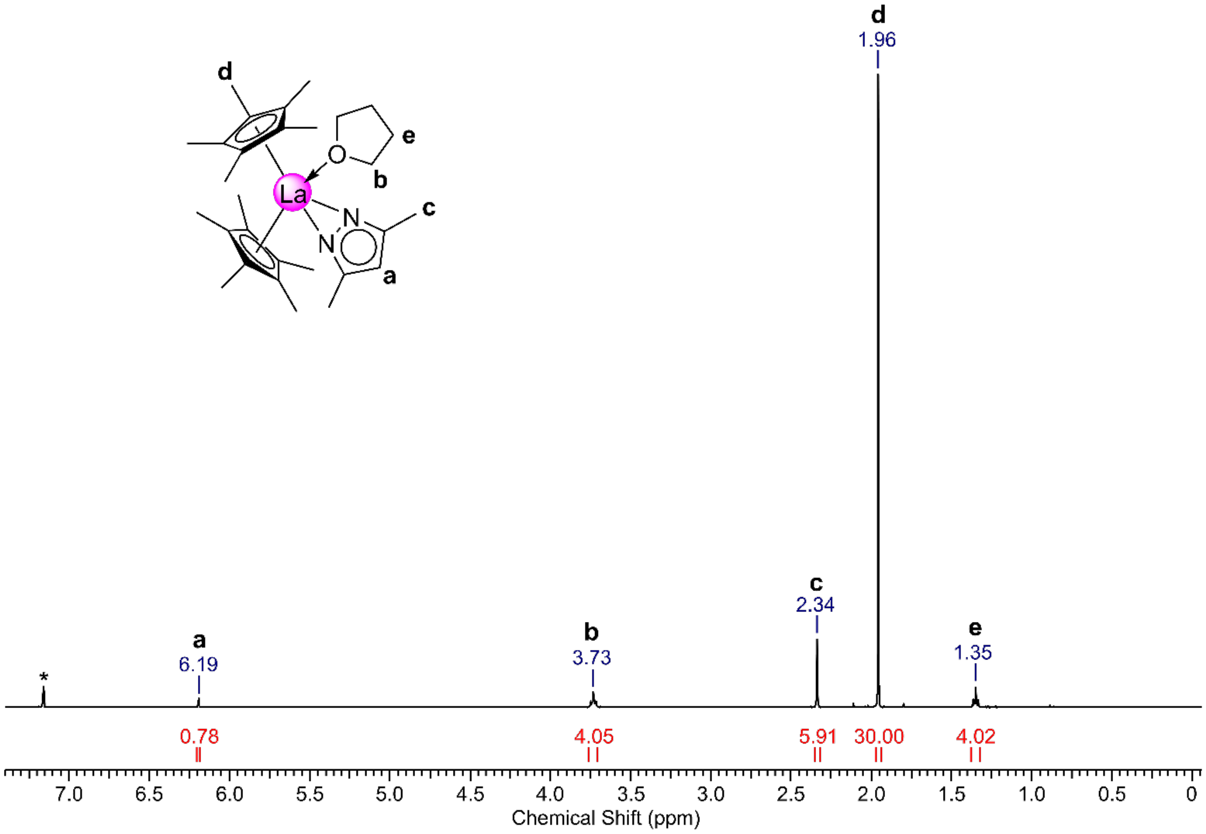

Figure S48. ^{1}H NMR spectrum (C_6D_6, 400.1 MHz, 26 °C) of $Cp^*_2La(pz^{Me,Me})(thf)$ (**1^{thf}-La**).

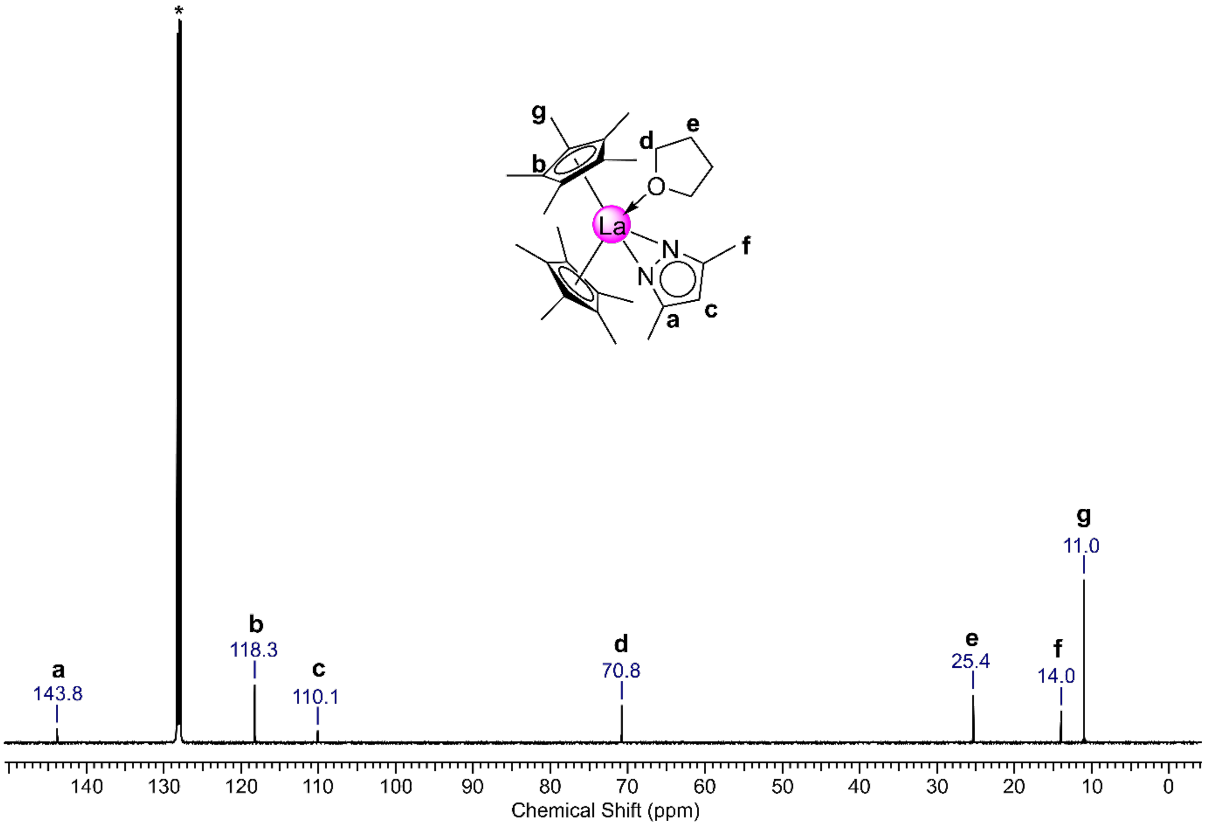

Figure S49. $^{13}C\{^{1}H\}$ NMR spectrum (C_6D_6, 100.6 MHz, 26 °C) of $Cp^*_2La(pz^{Me,Me})(thf)$ (**1^{thf}-La**).

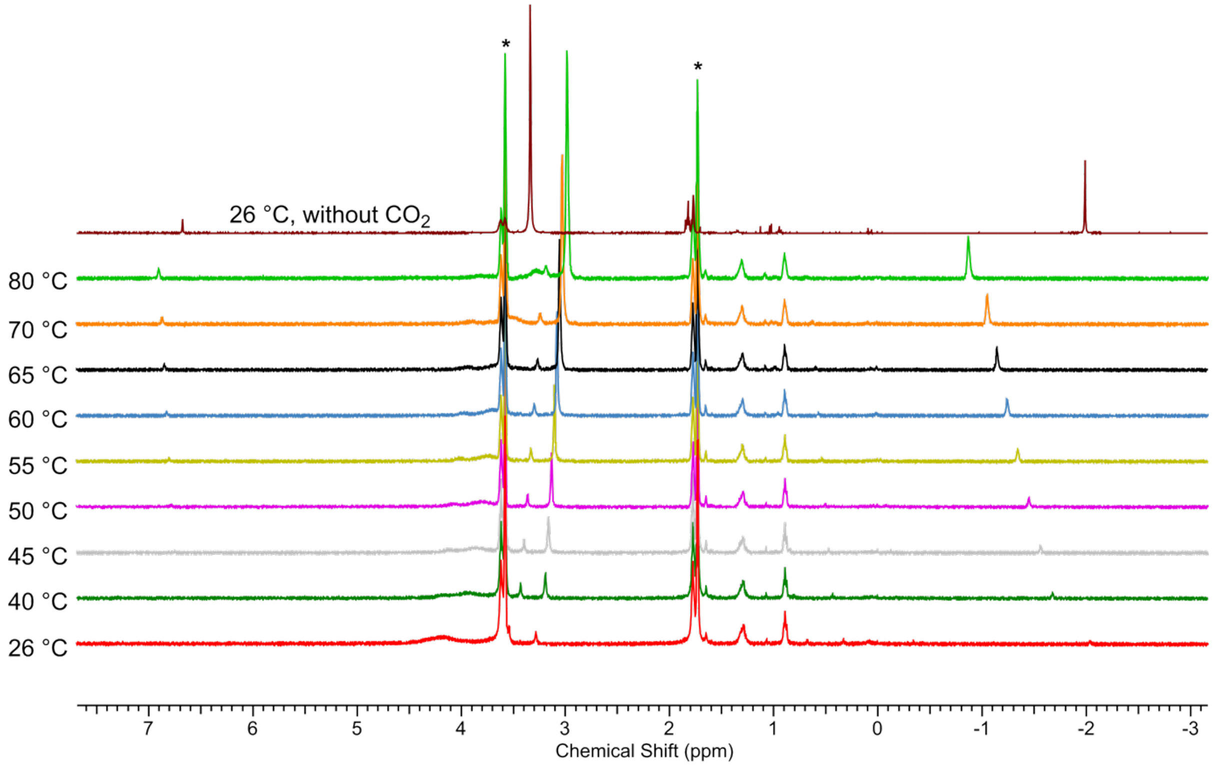

Figure S50. ^{1}H VT NMR spectrum (THF-d$_8$, 500.1 MHz) of $[Cp^*_2Ce(pz^{Me,Me}\cdot CO_2)]_2$ (**2**) in the range from 26 °C to 80 °C.

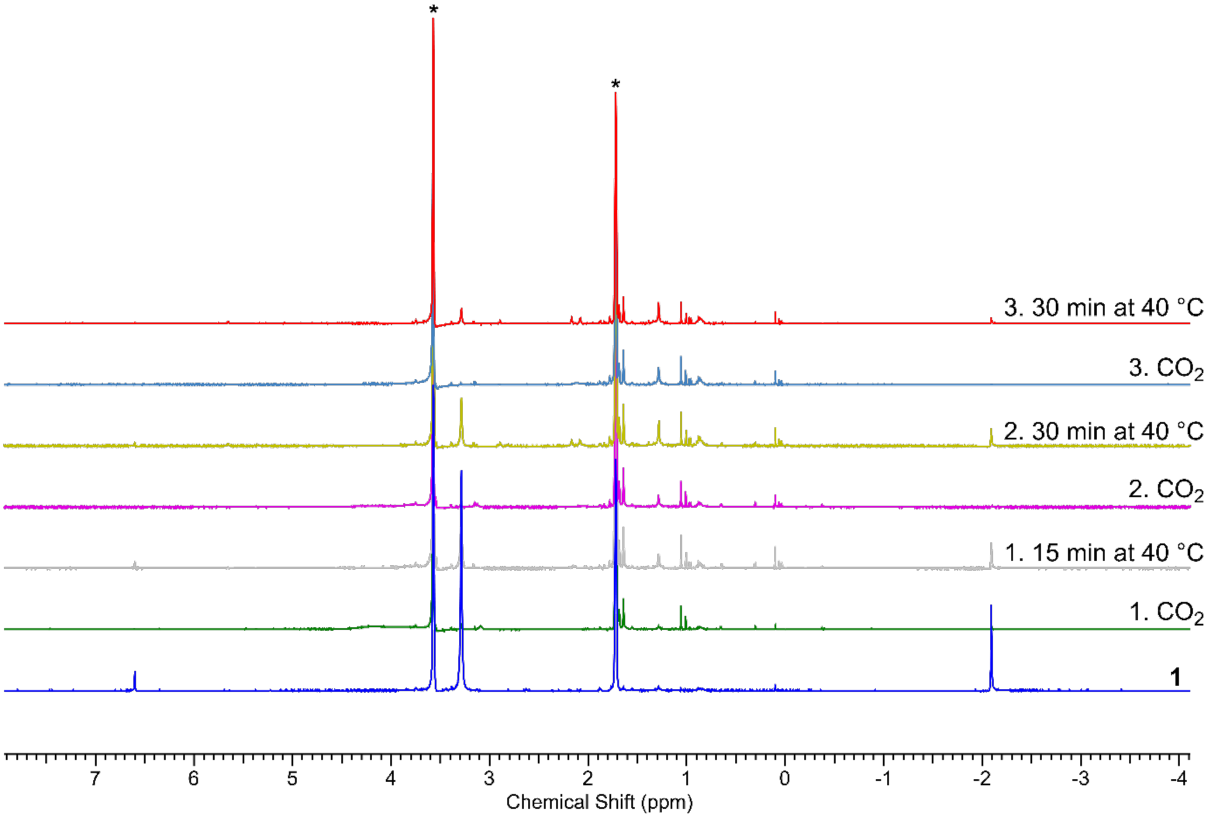

Figure S51. ^{1}H NMR spectra (THF-d$_8$, 400.1 MHz, 26 °C) of repeated addition of CO_2 to $Cp^*_2Ce(pz^{Me,Me})$ (**1**) and heating at 40 °C.

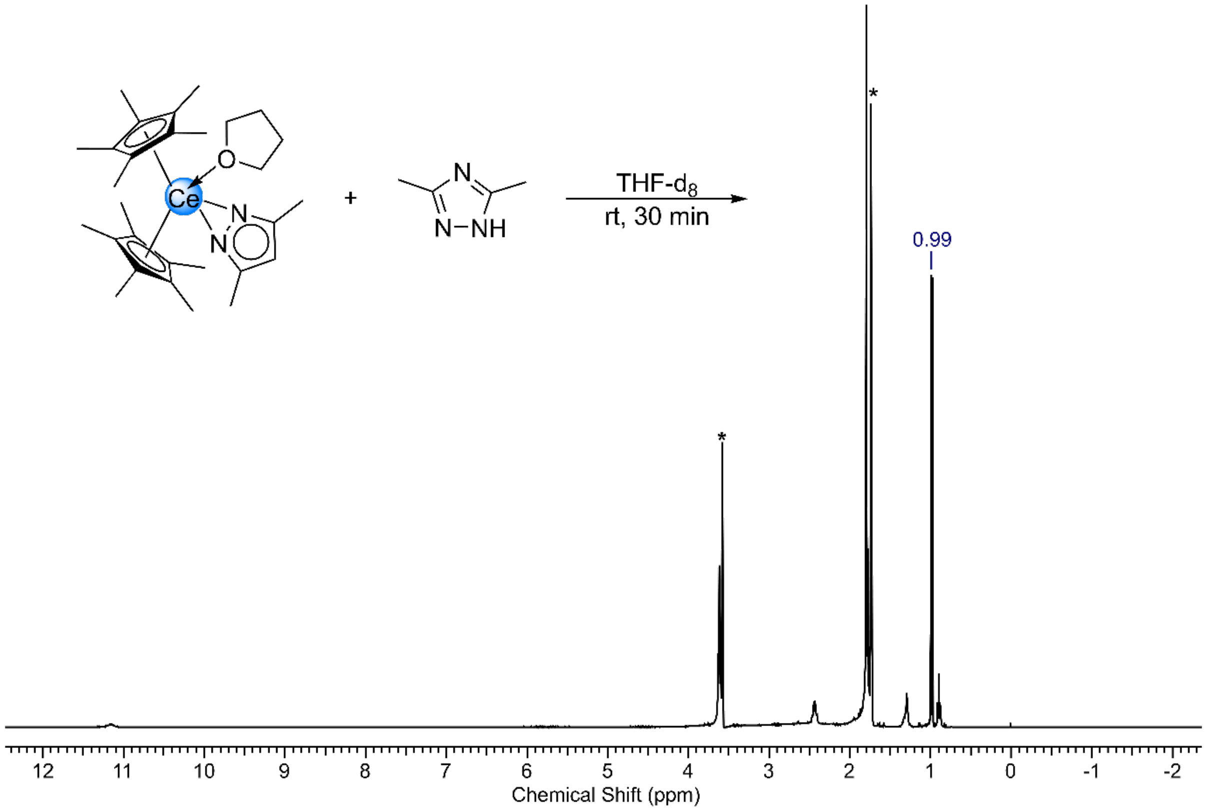

Figure S52. ^{1}H NMR spectrum (THF-d_8, 400.1 MHz, 26 °C) of the reaction of $\mathbf{1^{thf}}$ and $Htz^{Me,Me}$.

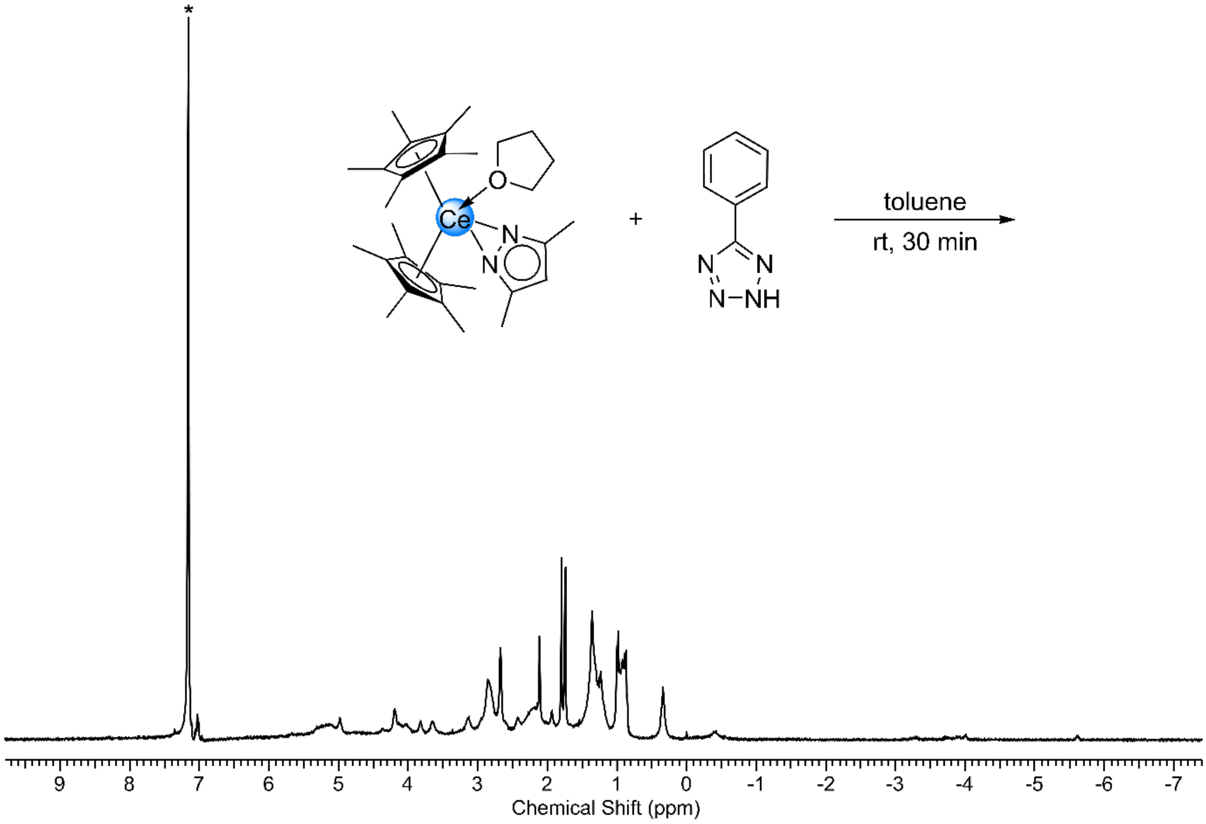

Figure S53. ^{1}H NMR spectrum (C_6D_6, 400.1 MHz, 26 °C) of the reaction of $\mathbf{1^{thf}}$ and $Htet^{Ph}$.

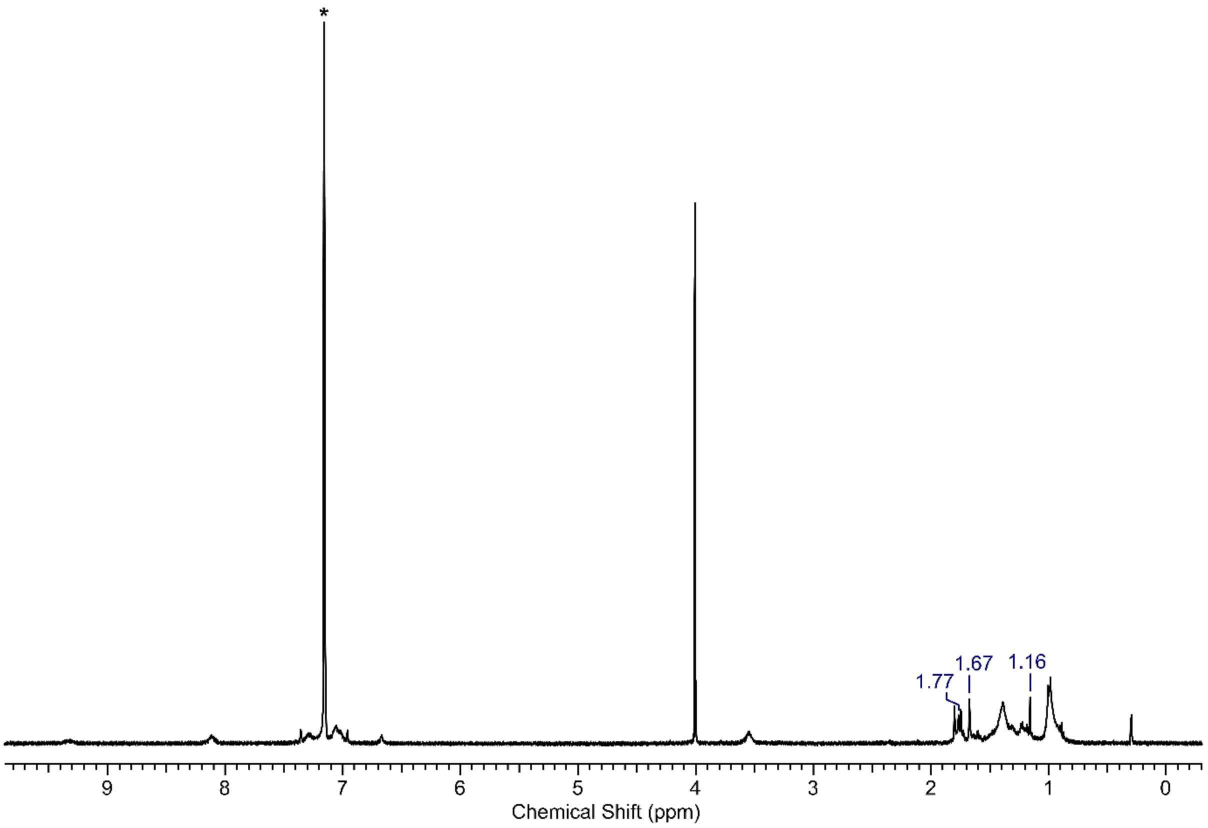

Figure S54. ^{1}H NMR spectrum (C_6D_6, 400.1 MHz, 26 °C) after the CV measurement of Cp*$_2$Ce(μ-pz^{Ph,Ph}·CO_2)CeCp*$_2$(pz^{Ph,Ph}) (**4**) in THF showing signals of Cp*$_2$.

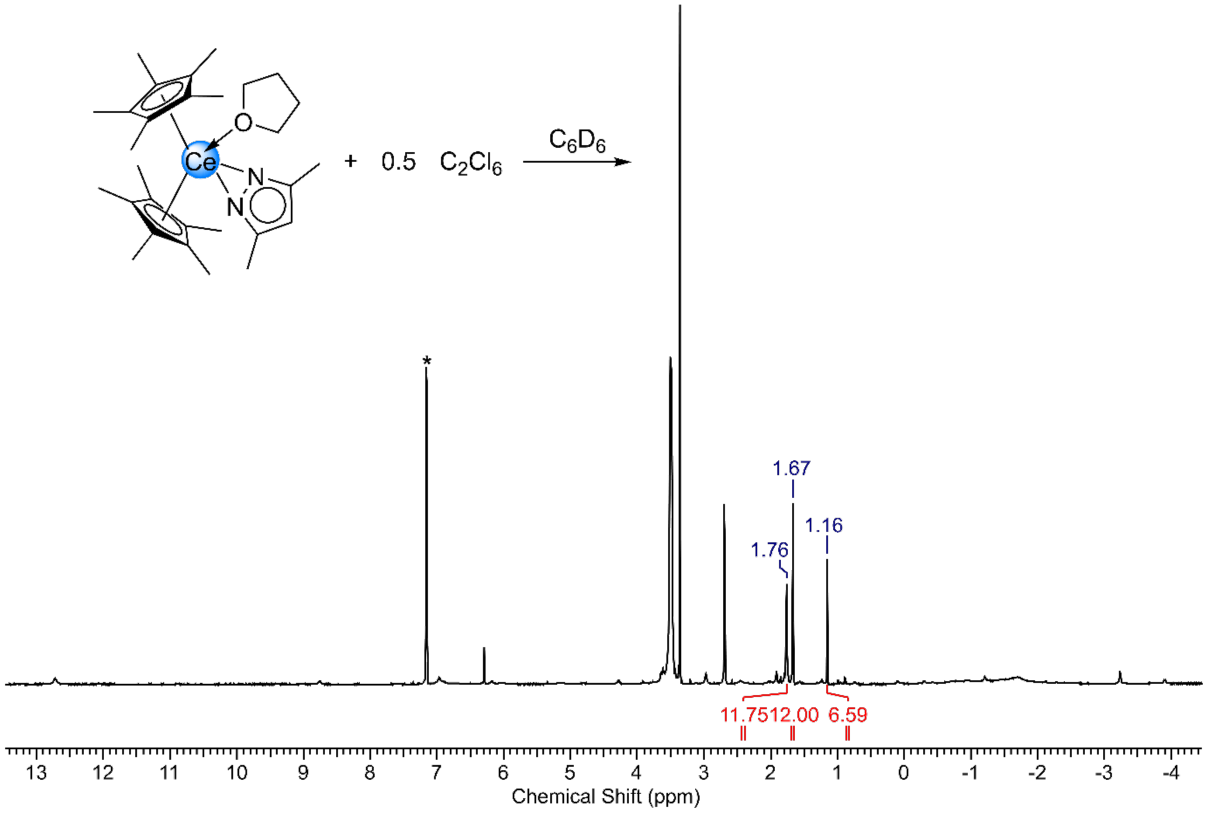

Figure S55. ^{1}H NMR spectrum (C_6D_6, 400.1 MHz, 26 °C) of the reaction of **1^{thf}** and C_2Cl_6.

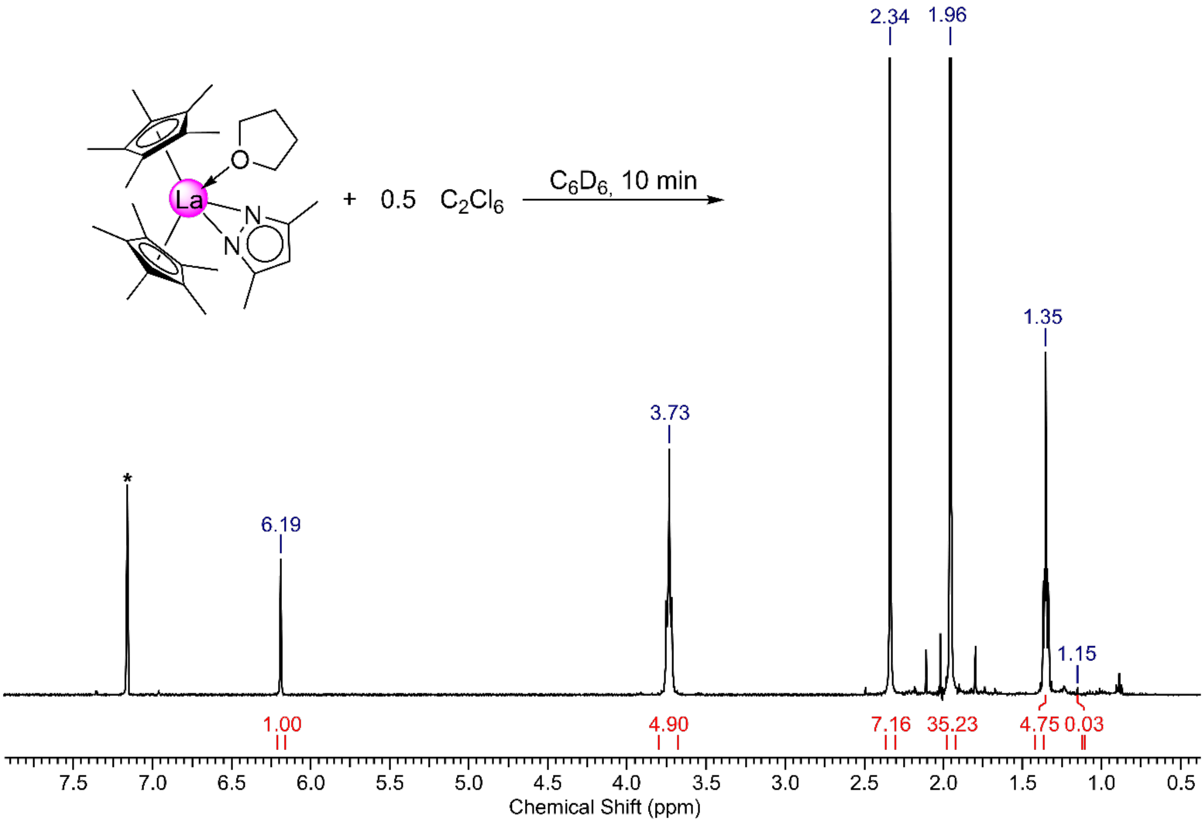

Figure S56. ^{1}H NMR spectrum (C_6D_6, 400.1 MHz, 26 °C) of the reaction of **10** and C_2Cl_6 after 10 min.

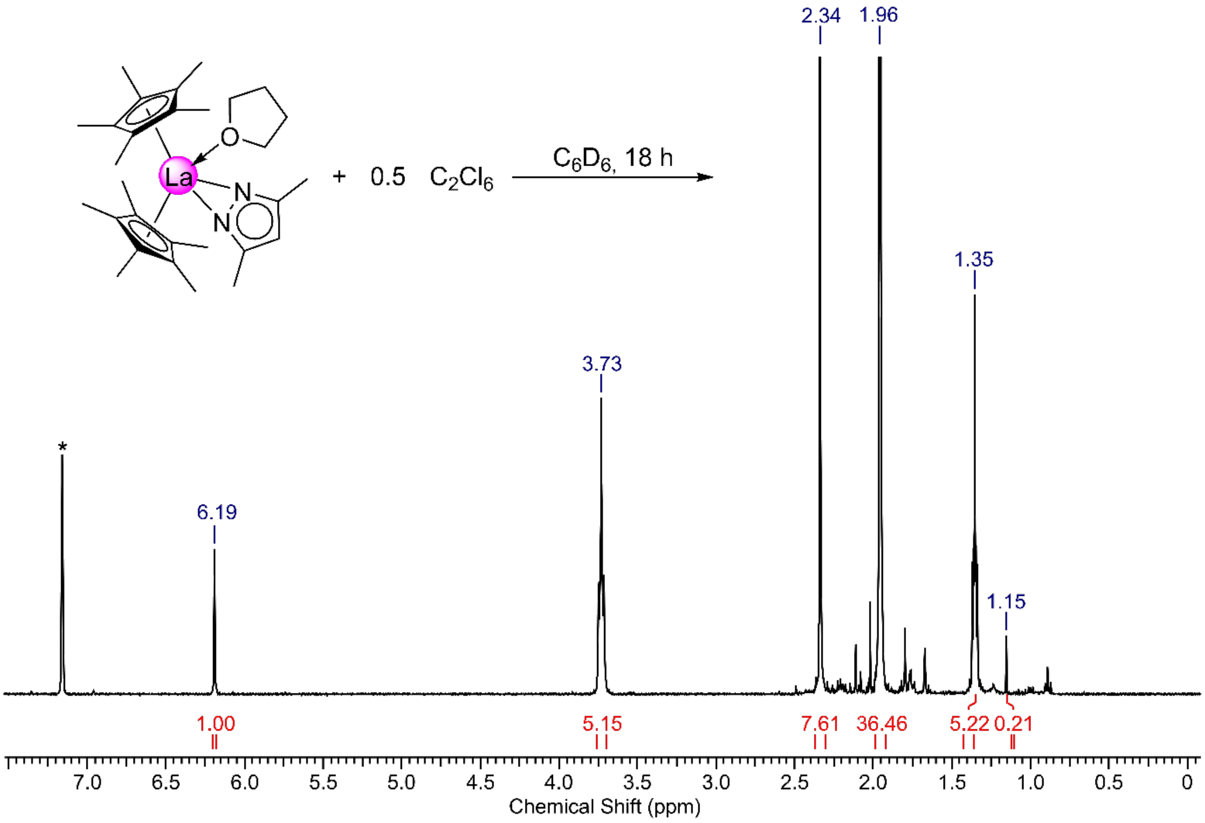

Figure S57. ^{1}H NMR spectrum (C_6D_6, 400.1 MHz, 26 °C) of the reaction of **10** and C_2Cl_6 after 18 h.

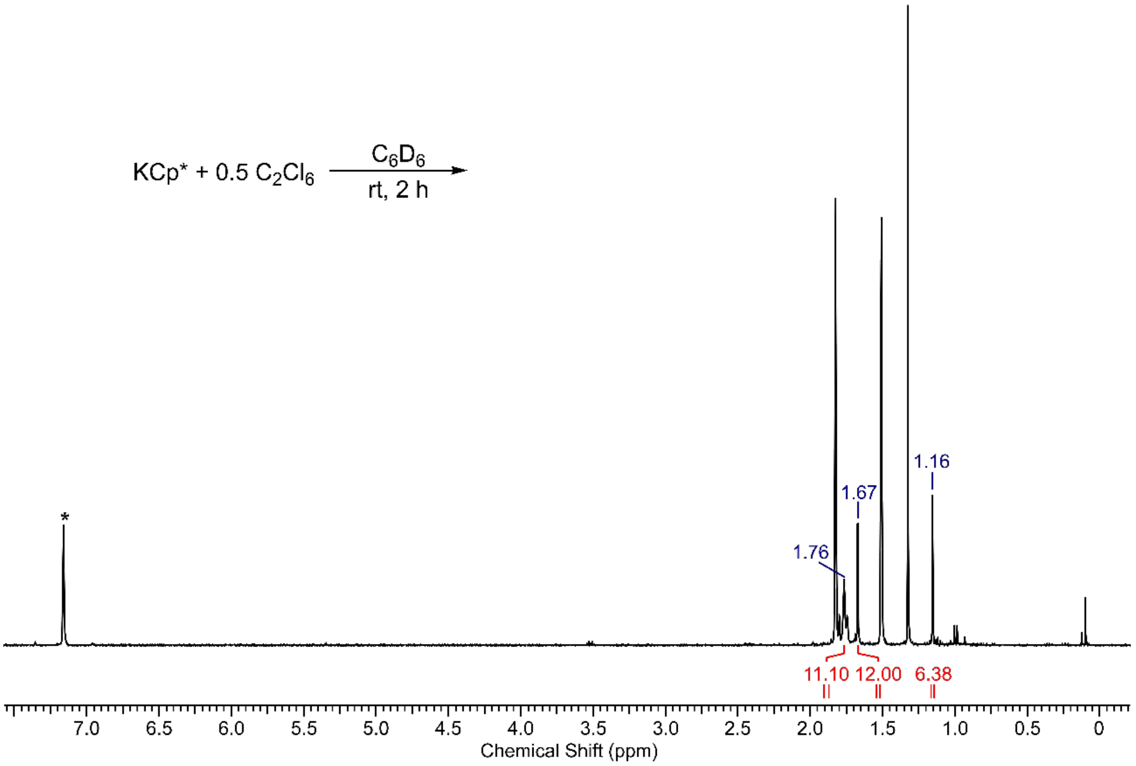

Figure S58. ^{1}H NMR spectrum (C_6D_6, 400.1 MHz, 26 °C) of the reaction of KCp* and C_2Cl_6.

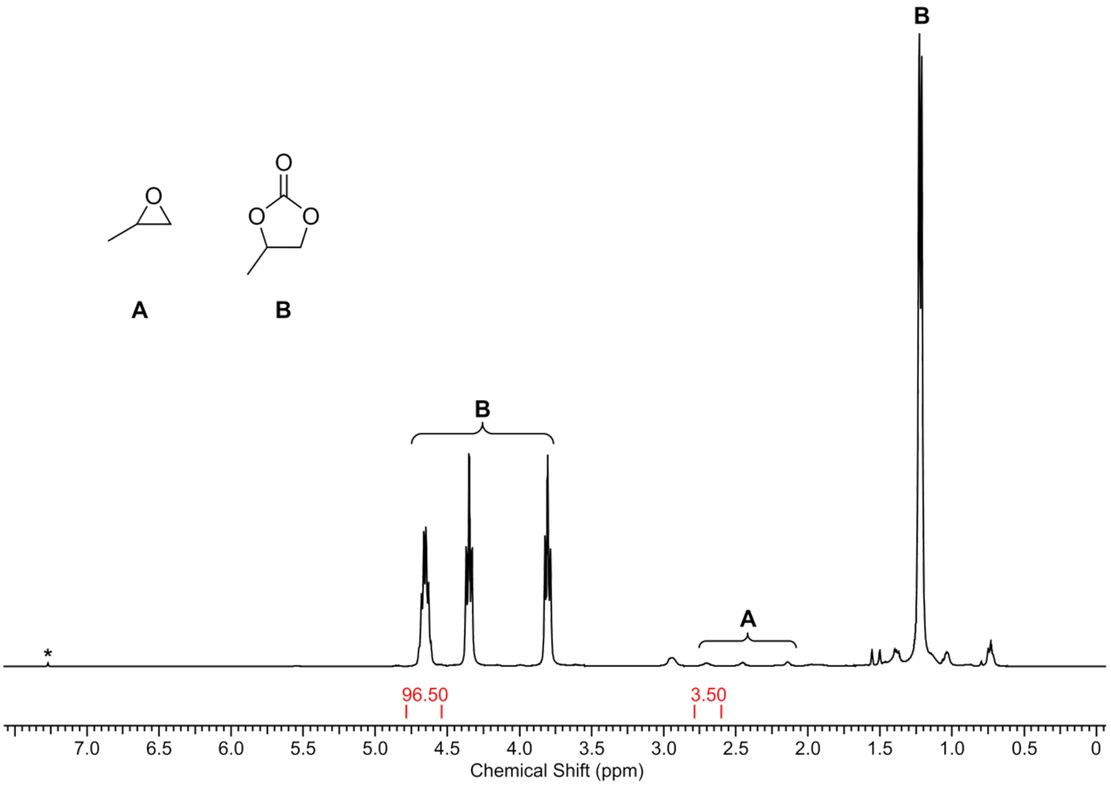

Figure S59. ^{1}H NMR spectrum ($CDCl_3$, 400.1 MHz, 26 °C) of the reaction mixture of the catalytic conversion of propylene oxide and CO_2 to propylene carbonate by using 0.5 mol% of $Cp^*{}_2Ce(pz^{Me,Me})$ (**1**) and 1 mol% TBAB.

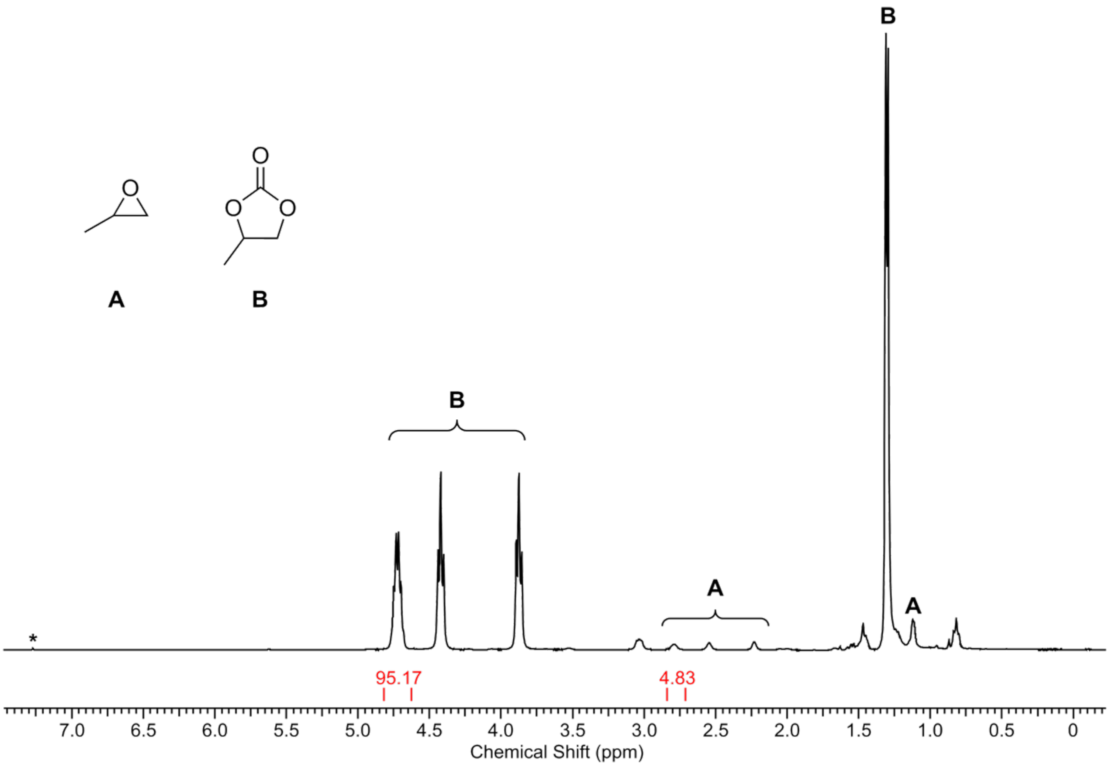

Figure S60. ^{1}H NMR spectrum ($CDCl_3$, 400.1 MHz, 26 °C) of the reaction mixture of the catalytic conversion of propylene oxide and CO_2 to propylene carbonate by using 0.5 mol% of $Cp^*{}_2Ce(pz^{Me,Me})(thf)$ ($\mathbf{1^{thf}}$) and 1 mol% TBAB.

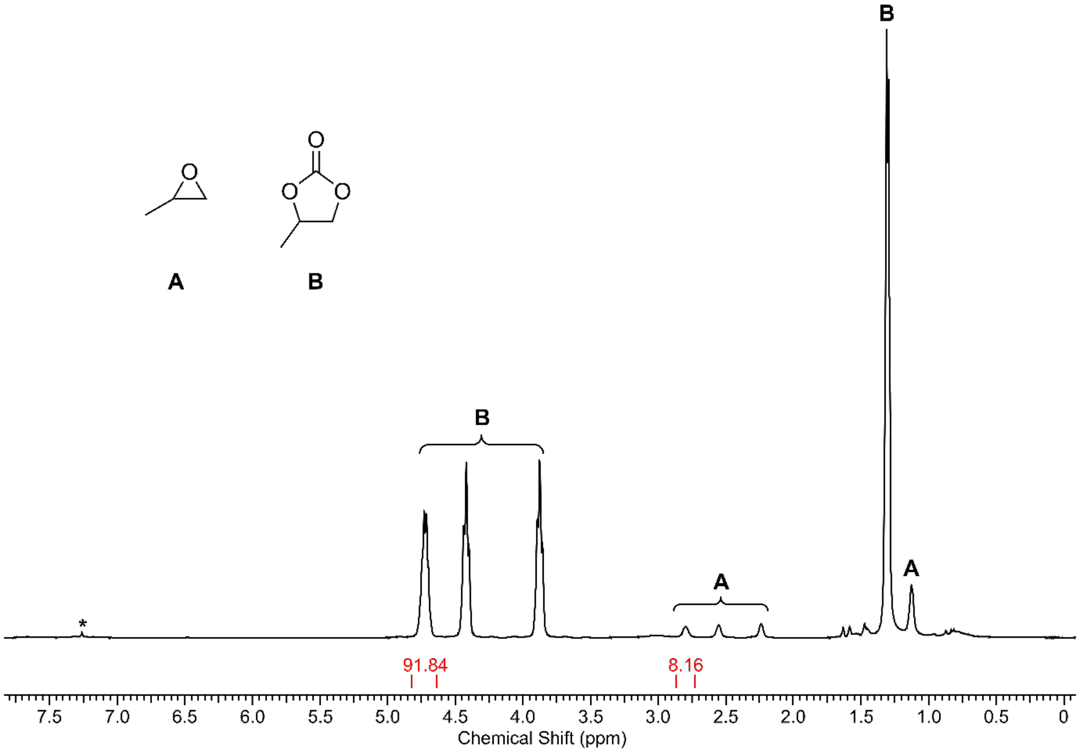

Figure S61. ^{1}H NMR spectrum ($CDCl_3$, 400.1 MHz, 26 °C) of the reaction mixture of the catalytic conversion of propylene oxide and CO_2 to propylene carbonate by using 0.5 mol% of $Cp^*{}_2Ce(pz^{Ph,Ph})$ (**3**) and 1 mol% TBAB.

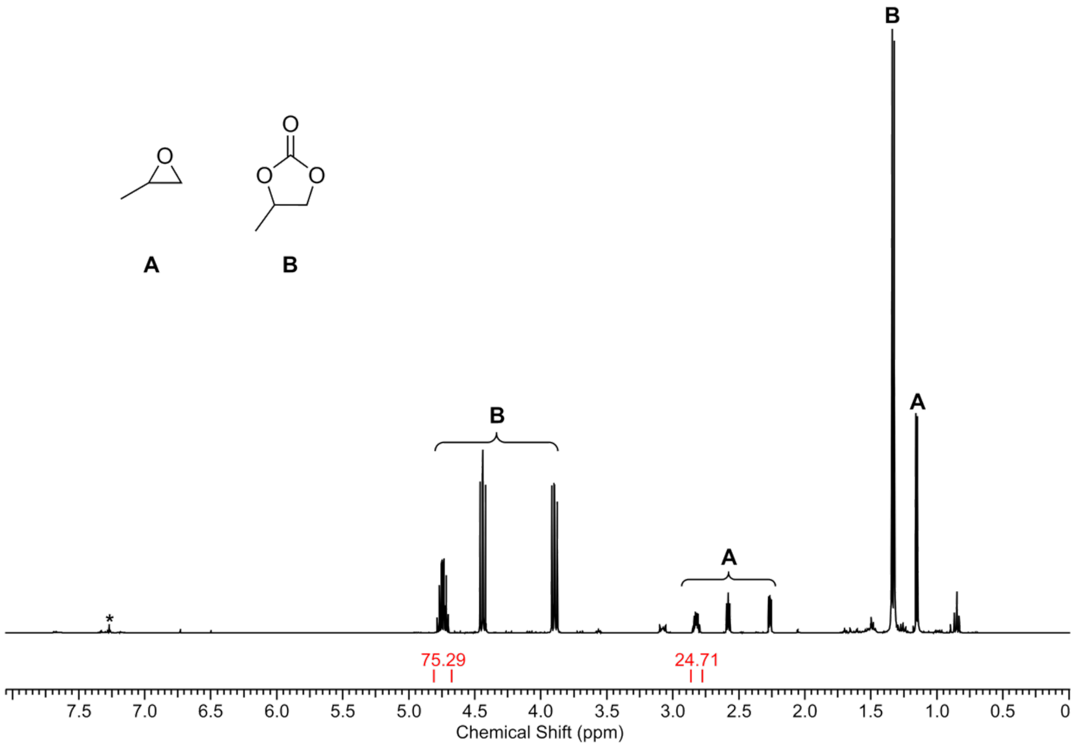

Figure S62. ^{1}H NMR spectrum ($CDCl_3$, 400.1 MHz, 26 °C) of the reaction mixture of the catalytic conversion of propylene oxide and CO_2 to propylene carbonate by using 0.5 mol% of Cp*$_2$Ce(pz^{Ph,Ph})(thf) (**3^{thf}**) and 1 mol% TBAB.

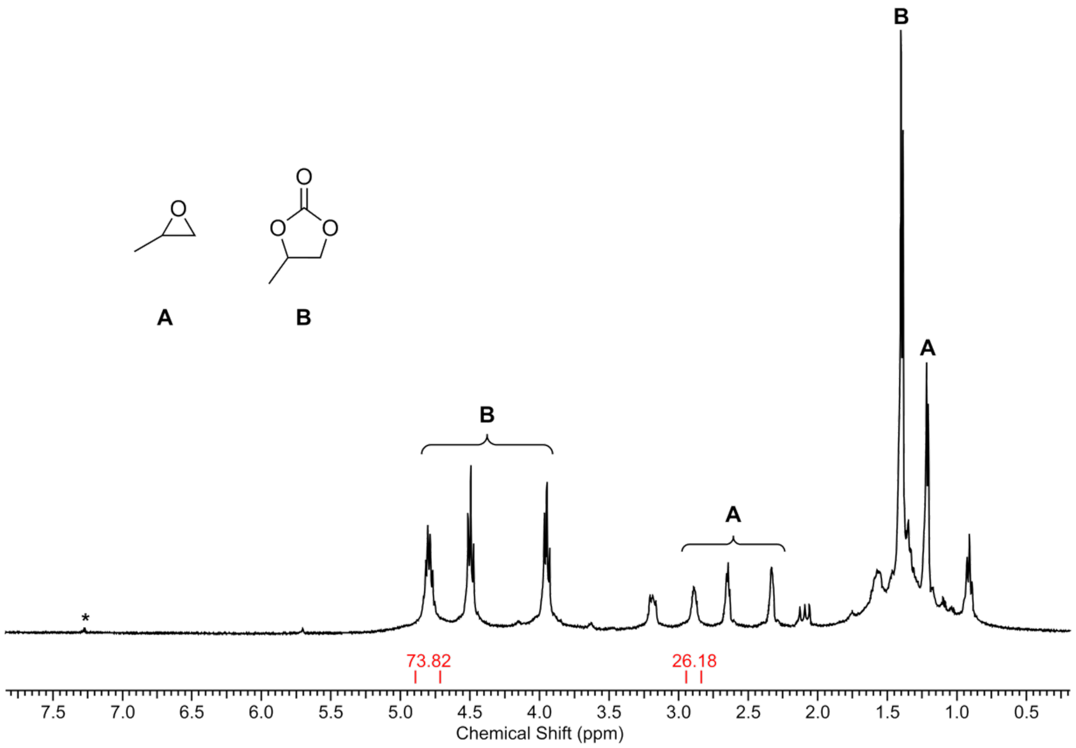

Figure S63. ^{1}H NMR spectrum ($CDCl_3$, 400.1 MHz, 26 °C) of the reaction mixture of the catalytic conversion of propylene oxide and CO_2 to propylene carbonate by using 0.5 mol% of Cp*Ce(pz^{Me,Me})$_2$(thf)$_2$ (**5**) and 1 mol% TBAB.

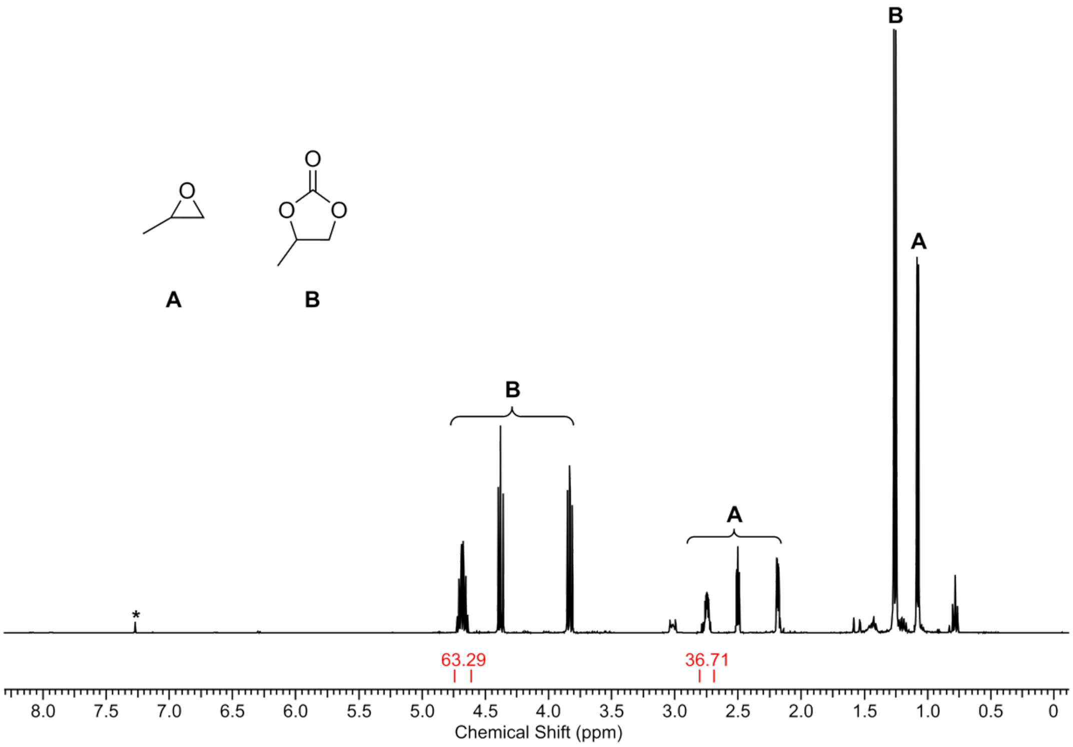

Figure S64. ^{1}H NMR spectrum ($CDCl_3$, 400.1 MHz, 26 °C) of the reaction mixture of the catalytic conversion of propylene oxide and CO_2 to propylene carbonate by using 0.5 mol% of Cp*$_2$Ce(tz^{Me,Me})(dmap) (**7**) and 1 mol% TBAB.

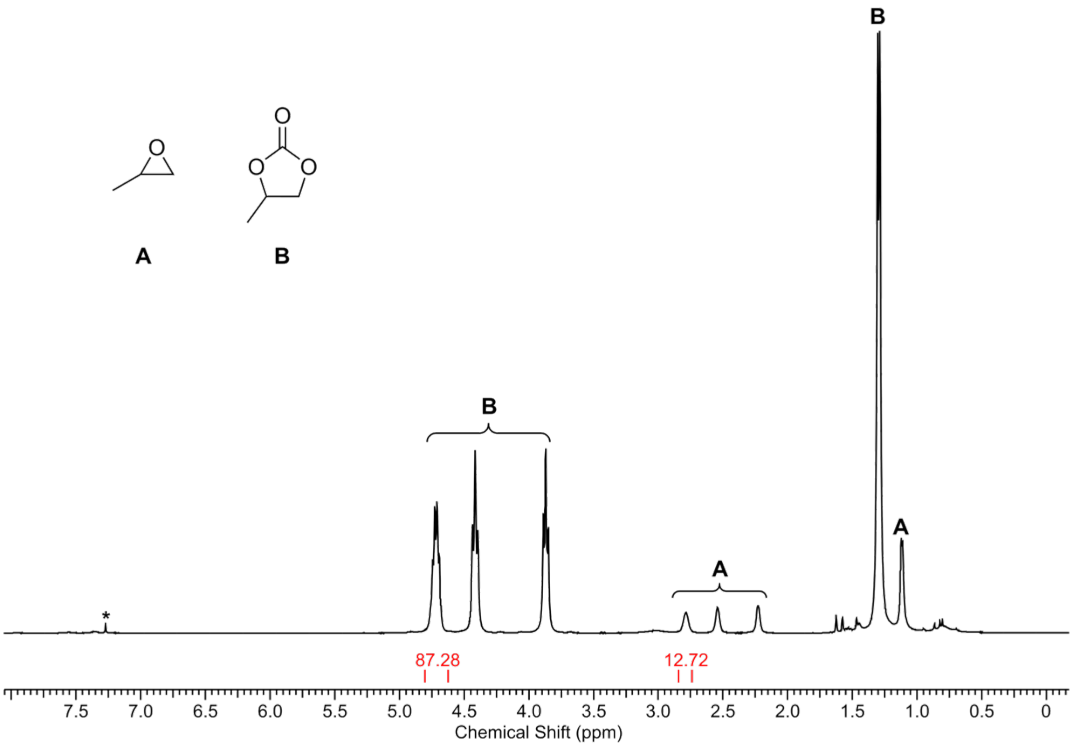

Figure S65. ^{1}H NMR spectrum ($CDCl_3$, 400.1 MHz, 26 °C) of the reaction mixture of the catalytic conversion of propylene oxide and CO_2 to propylene carbonate by using 0.5 mol% of Cp*$_2$Ce(tz^{Ph,Ph}) (**8**) and 1 mol% TBAB.

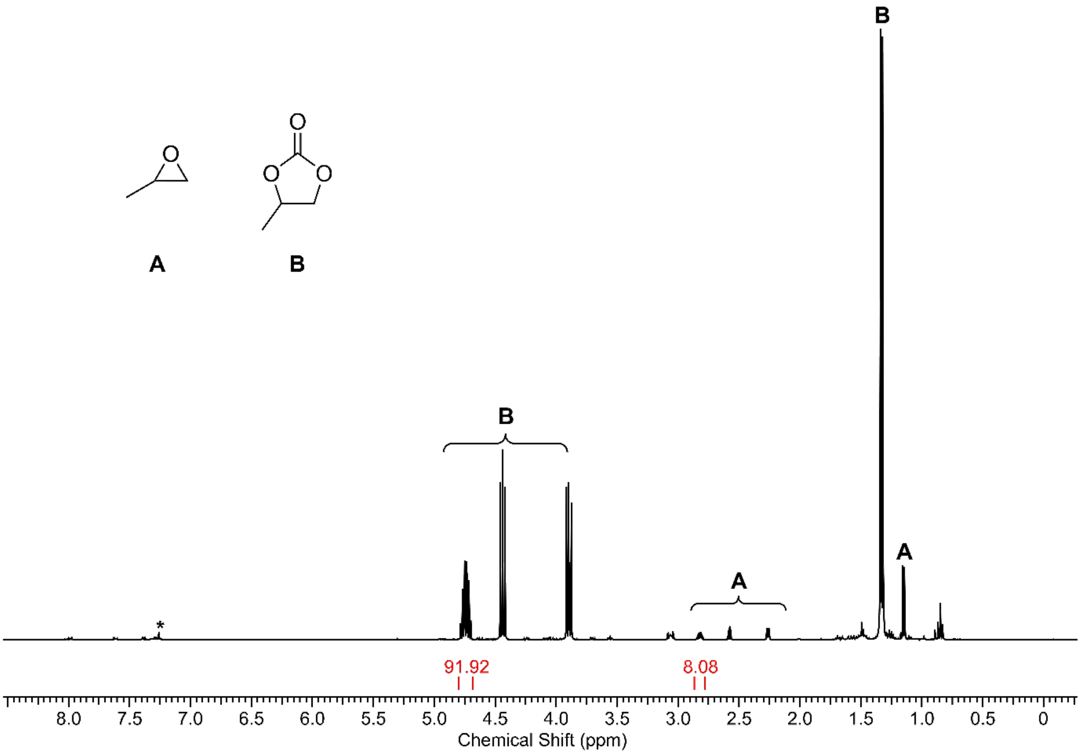

Figure S66. ^{1}H NMR spectrum ($CDCl_3$, 400.1 MHz, 26 °C) of the reaction mixture of the catalytic conversion of propylene oxide and CO_2 to propylene carbonate by using 0.5 mol% of $Cp^*{}_2Ce(tz^{Ph,Ph})(thf)$ (**8**$^{\mathbf{thf}}$) and 1 mol% TBAB.

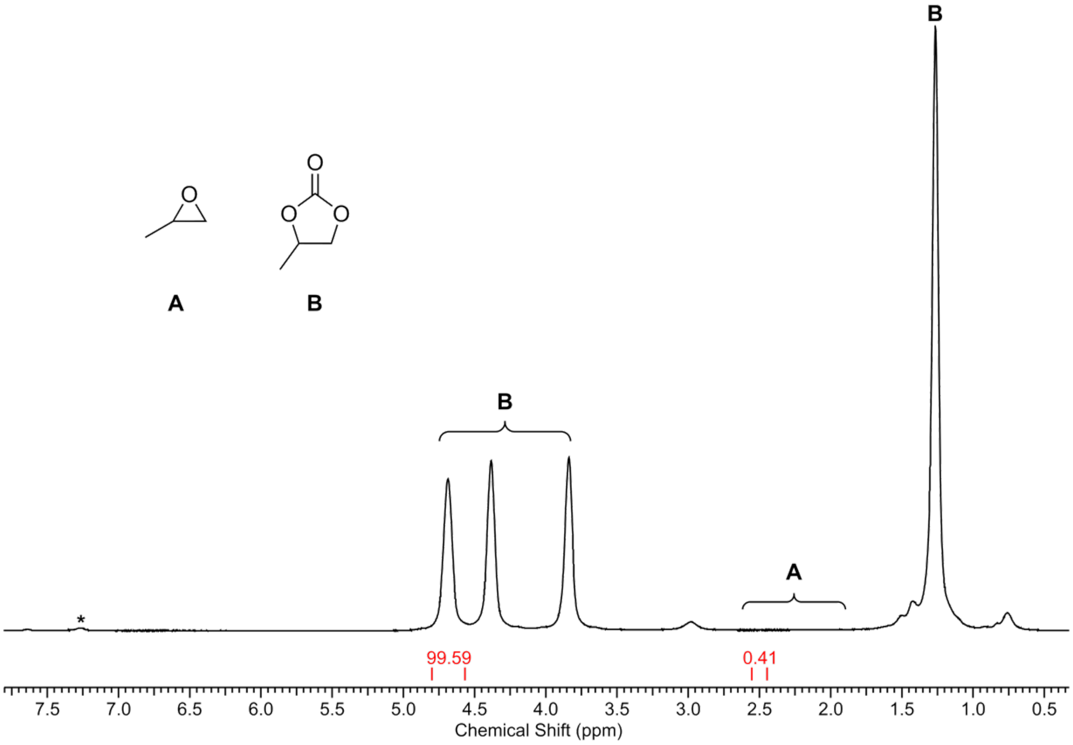

Figure S67. ^{1}H NMR spectrum ($CDCl_3$, 400.1 MHz, 26 °C) of the reaction mixture of the catalytic conversion of propylene oxide and CO_2 to propylene carbonate by using 0.5 mol% of $[Cp^*{}_2Ce(tet^{Ph})]_3$ (**9**) (in relation to Ce) and 1 mol% TBAB.

IR Spectra

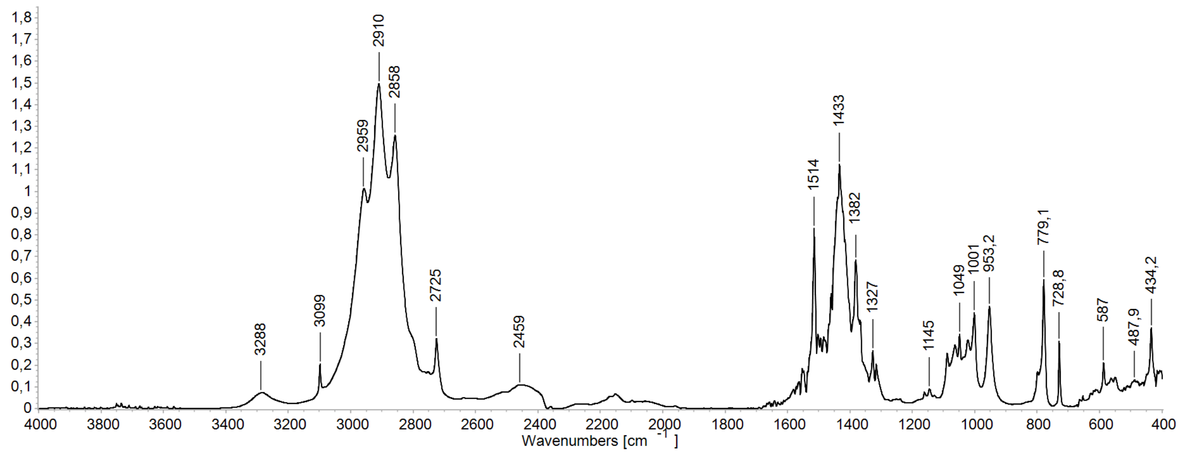

Figure S68. DRIFT spectrum of $Cp^{*}_2Ce(pz^{Me,Me})$ (**1**).

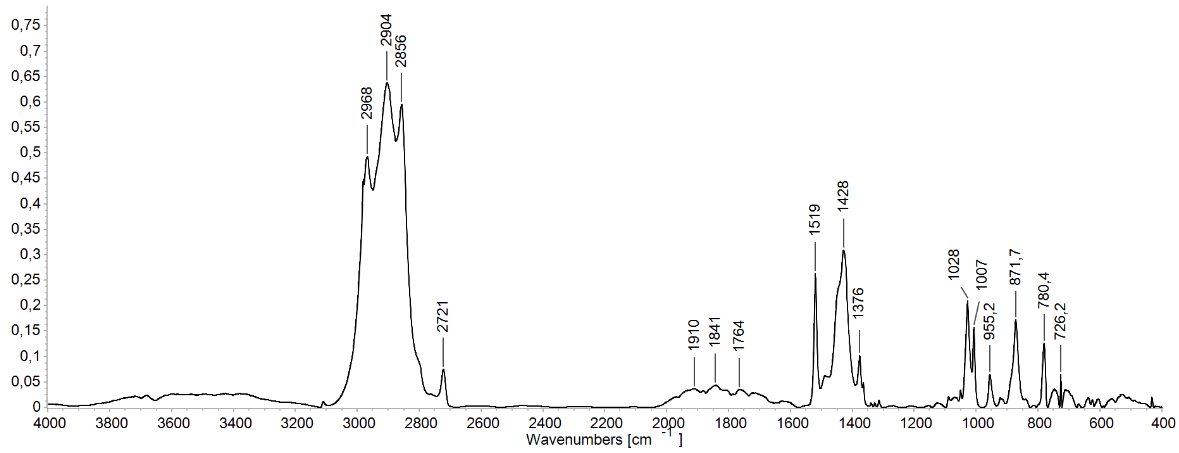

Figure S69. DRIFT spectrum of $Cp^{*}_2Ce(pz^{Me,Me})(thf)$ ($\mathbf{1}^{\mathbf{thf}}$).

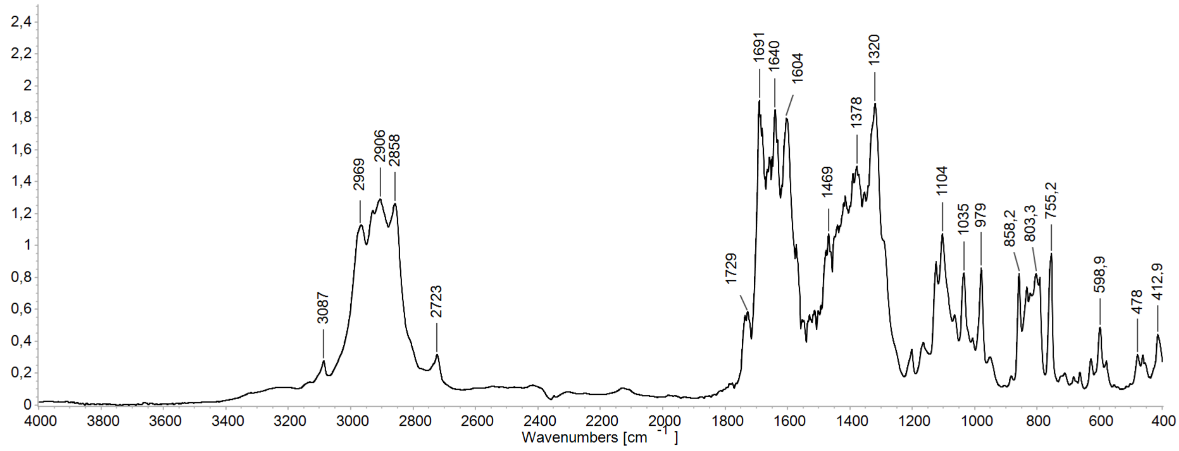

Figure S70. DRIFT spectrum of $[Cp^{*}_2Ce(pz^{Me,Me}\cdot CO_2)]_2$ (**2**).

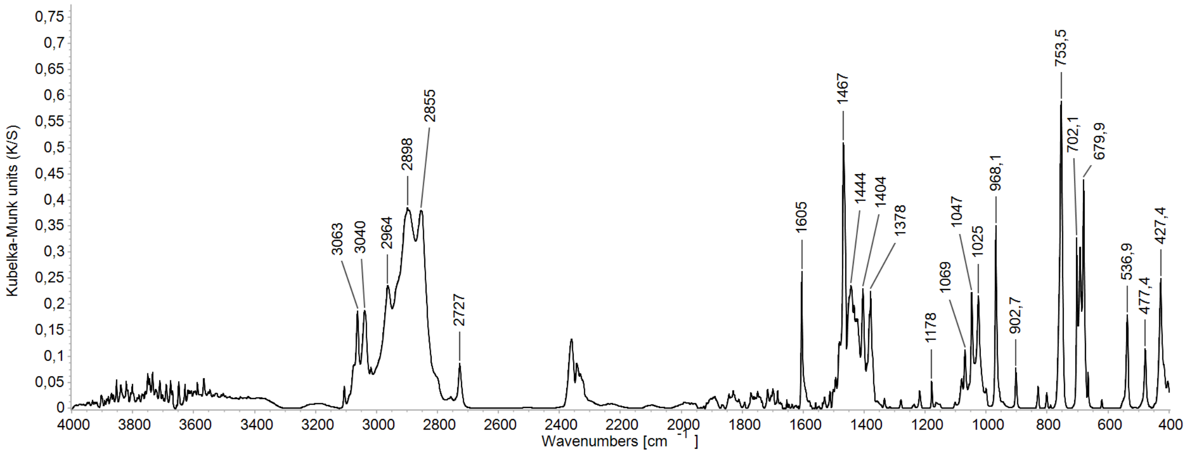

Figure S71. DRIFT spectrum of $Cp^*{}_2Ce(pz^{Ph,Ph})$ (**3**).

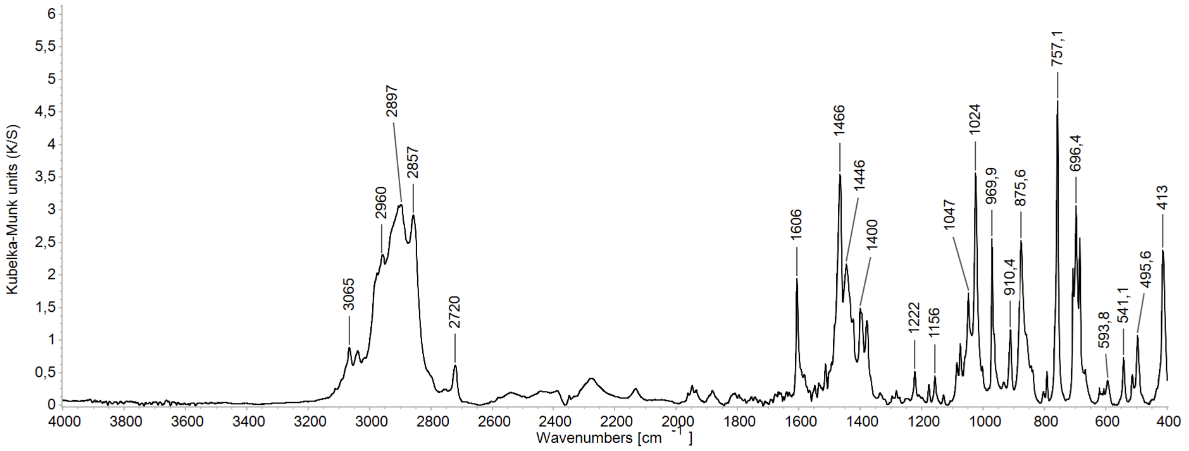

Figure S72. DRIFT spectrum of $Cp^*{}_2Ce(pz^{Ph,Ph})(thf)$ (**3**$^{\mathbf{thf}}$).

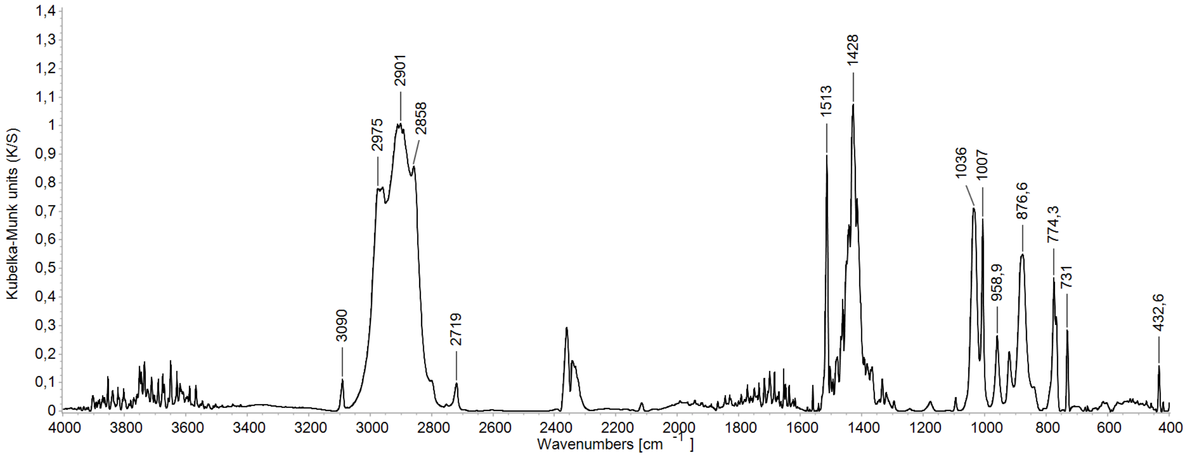

Figure S73. DRIFT spectrum of $Cp^*Ce(pz^{Me,Me})_2(thf)_2$ (**5**).

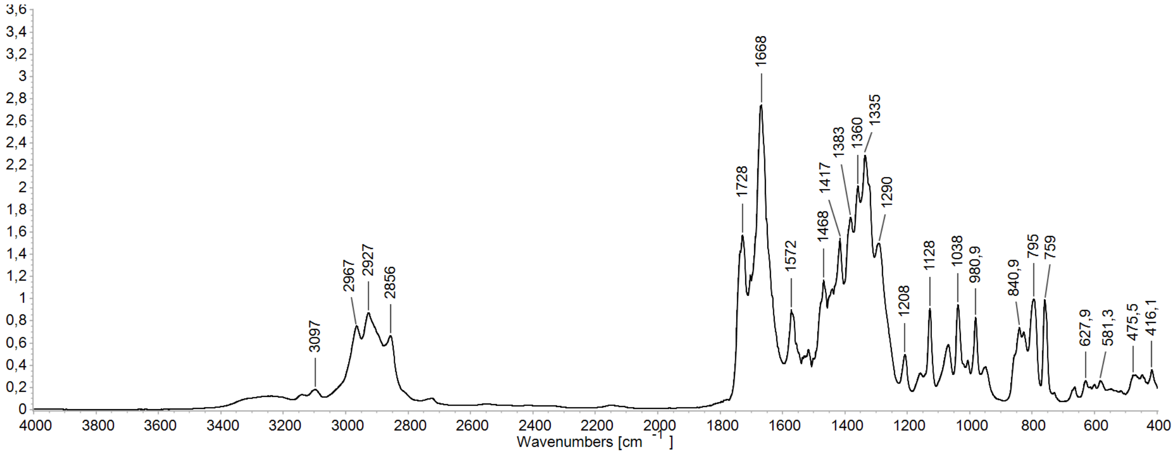

Figure S74. DRIFT spectrum of [Cp*Ce(pz^{Me,Me}·CO_2)$_2$(thf)]$_2$ (**6**).

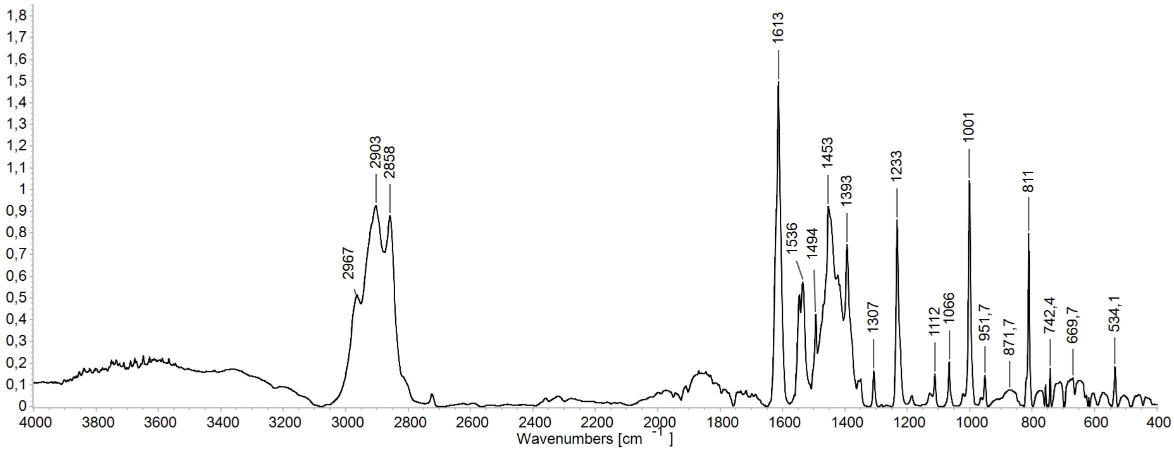

Figure S75. DRIFT spectrum of Cp*$_2$Ce(tz^{Me,Me})(dmap) (**7**).

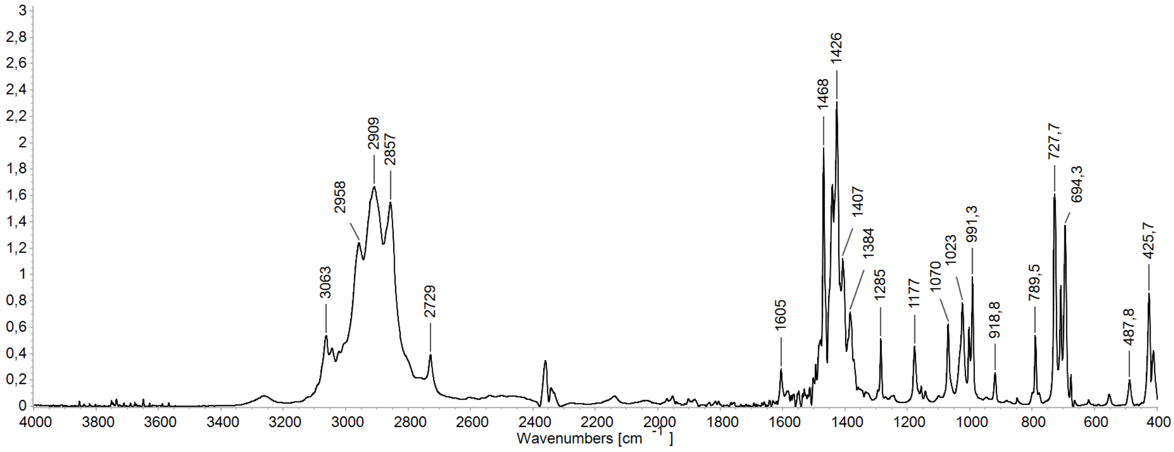

Figure S76. DRIFT spectrum of Cp*$_2$Ce(tz^{Ph,Ph}) (**8**).

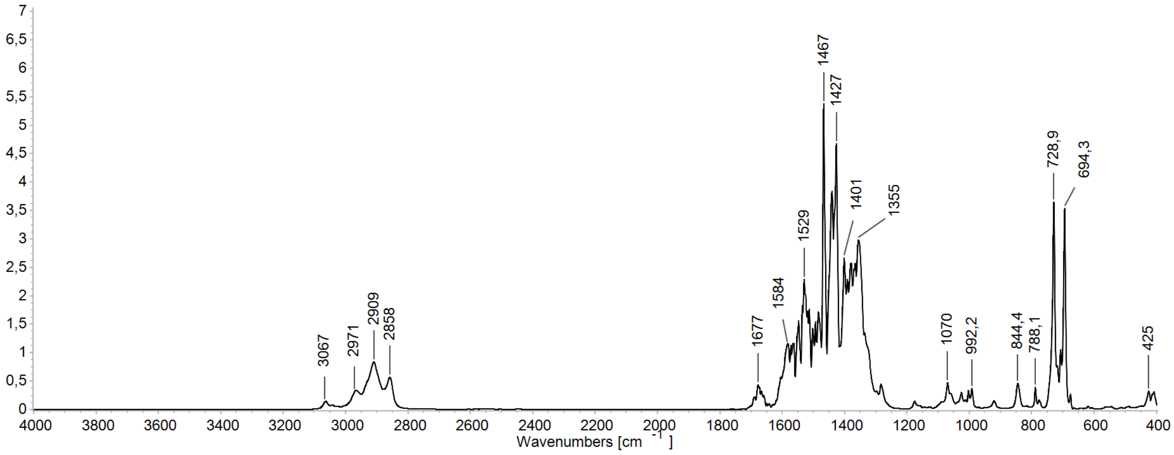

Figure S77. DRIFT spectrum of the reaction of Cp*$_2$Ce(tz^{Ph,Ph}) (**8**) and CO_2.

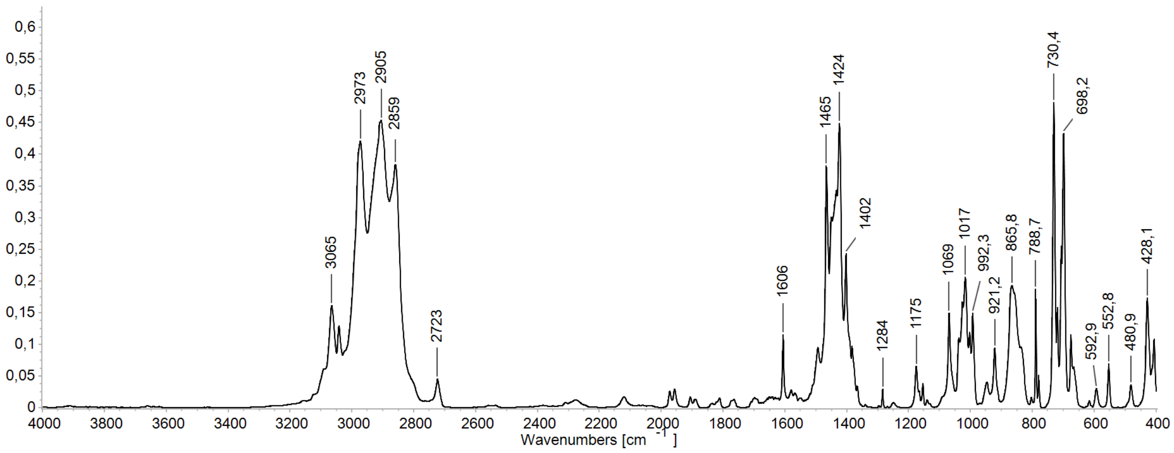

Figure S78. DRIFT spectrum of Cp*$_2$Ce(tz^{Ph,Ph})(thf) (**8^{thf}**).

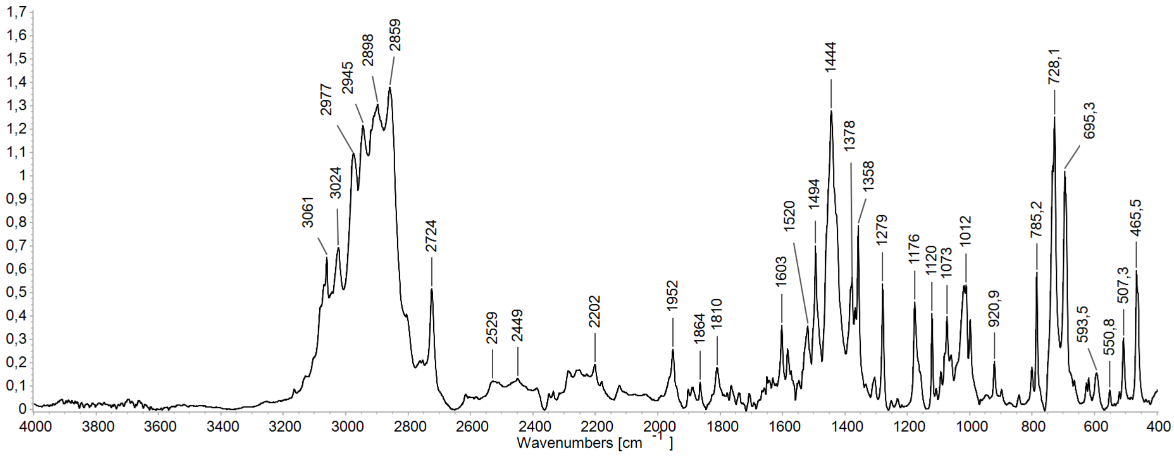

Figure S79. DRIFT spectrum of [Cp*$_2$Ce(tet^{Ph})]$_3$ (**9-Ce**).

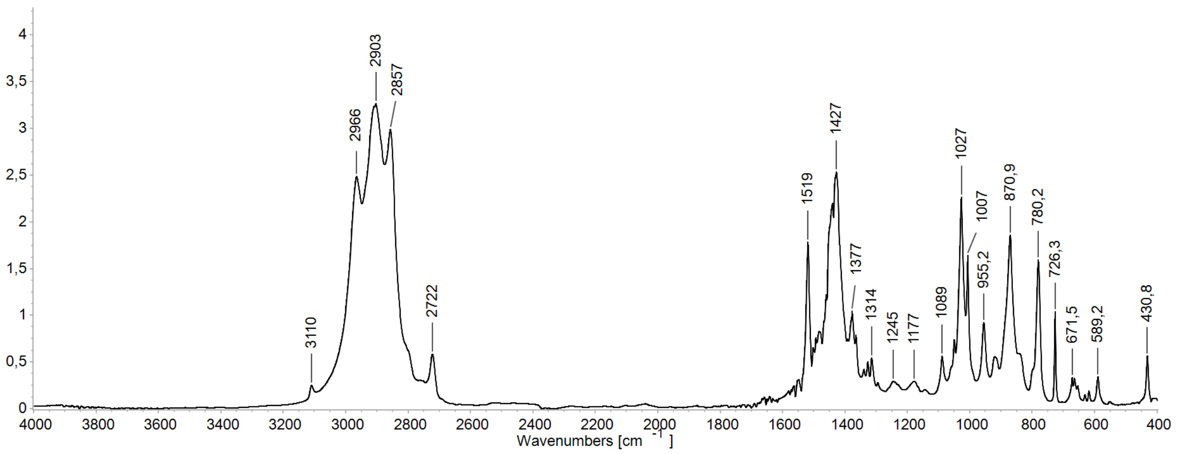

Figure S80. DRIFT spectrum of $Cp^*_2La(pz^{Me,Me})(thf)$ (**1^{thf}-La**).

Thermogravimetry

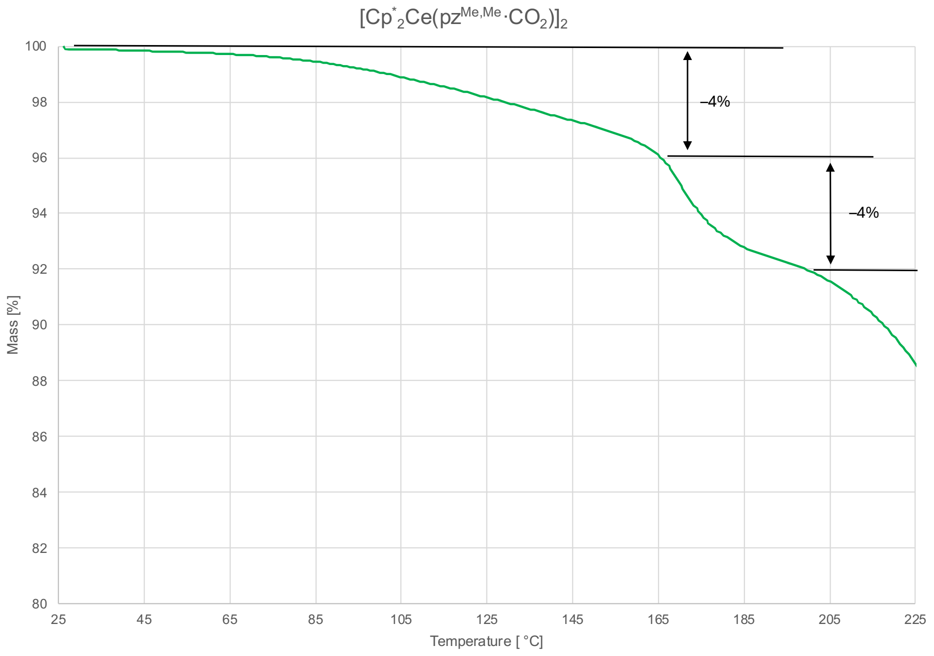

Figure S81. Thermogravimetric analysis of $[Cp^{*}_{2}Ce(pz^{Me,Me}\cdot CO_2)]_2$ (**2**). Sample was heated from 25 °C to 225 °C with a heating ratio of 0.5 $Kmin^{-1}$ under constant Ar flow.

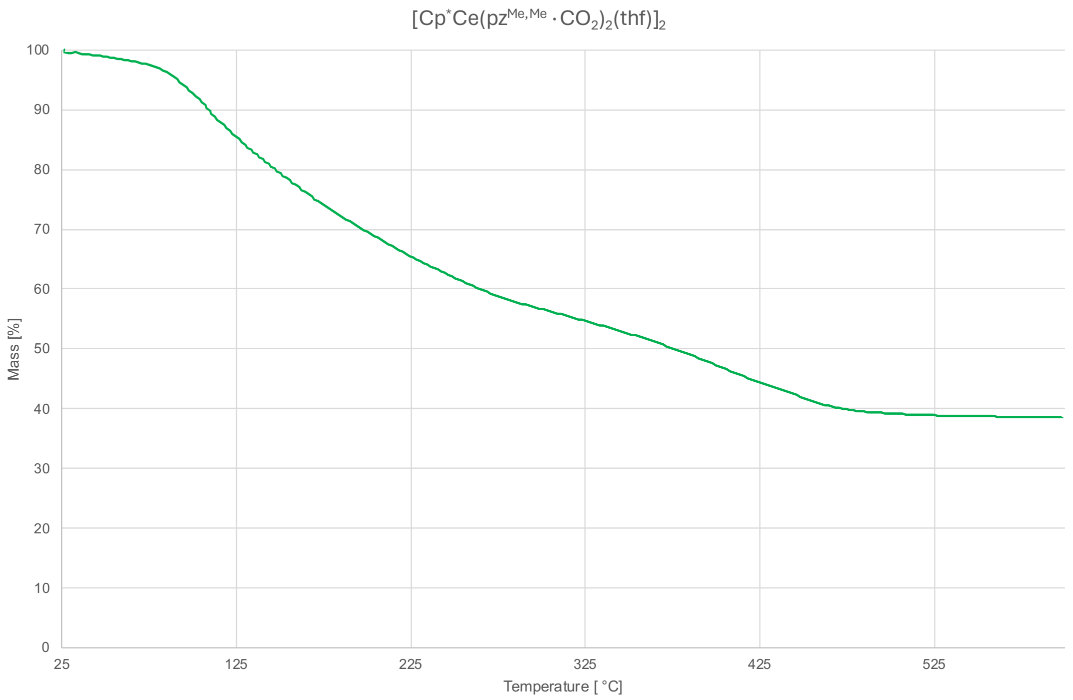

Figure S82. Thermogravimetric analysis of $[Cp^{*}Ce(pz^{Me,Me}\cdot CO_2)_2(thf)]_2$ (**6**). Sample was heated from 26 °C to 600 °C with a heating ratio of 2 $Kmin^{-1}$ under constant Ar flow.

Absorption Spectra

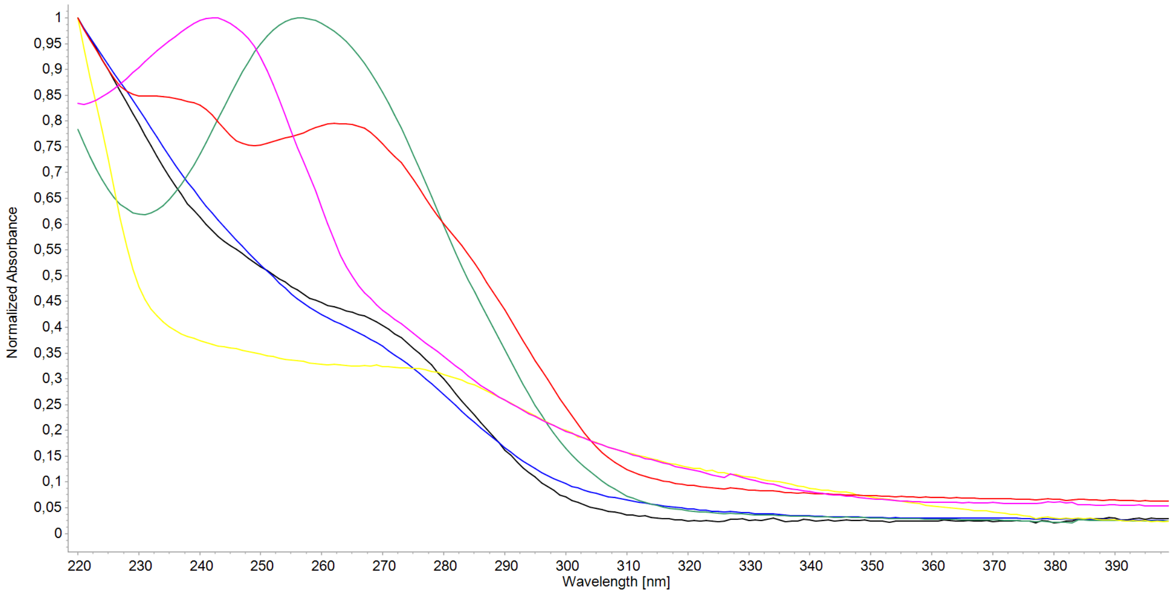

Figure S83. Normalized absorption spectra of **1** (black), **1**^{thf} (blue), **3** (green), **5** (yellow), **8** (red) and **9** (magenta) in *n*-hexane.

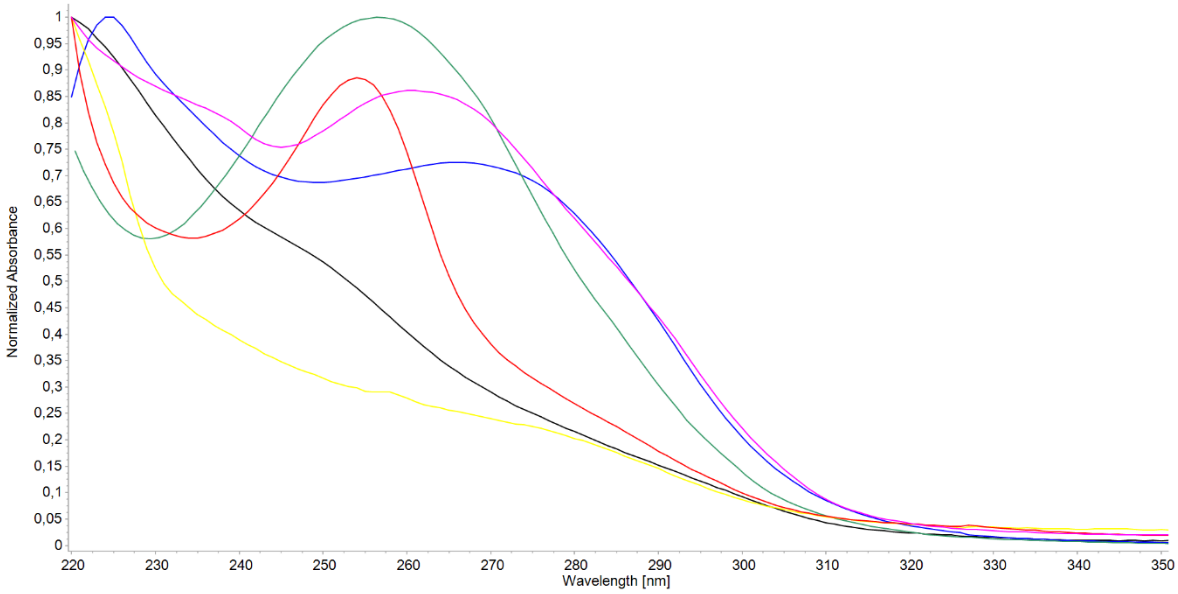

Figure S84. Normalized absorption spectra of **1**^{thf} (black), **2** (blue), **3**^{thf} (green), **6** (yellow), **7** (red) and **8**^{thf} (magenta) in THF.

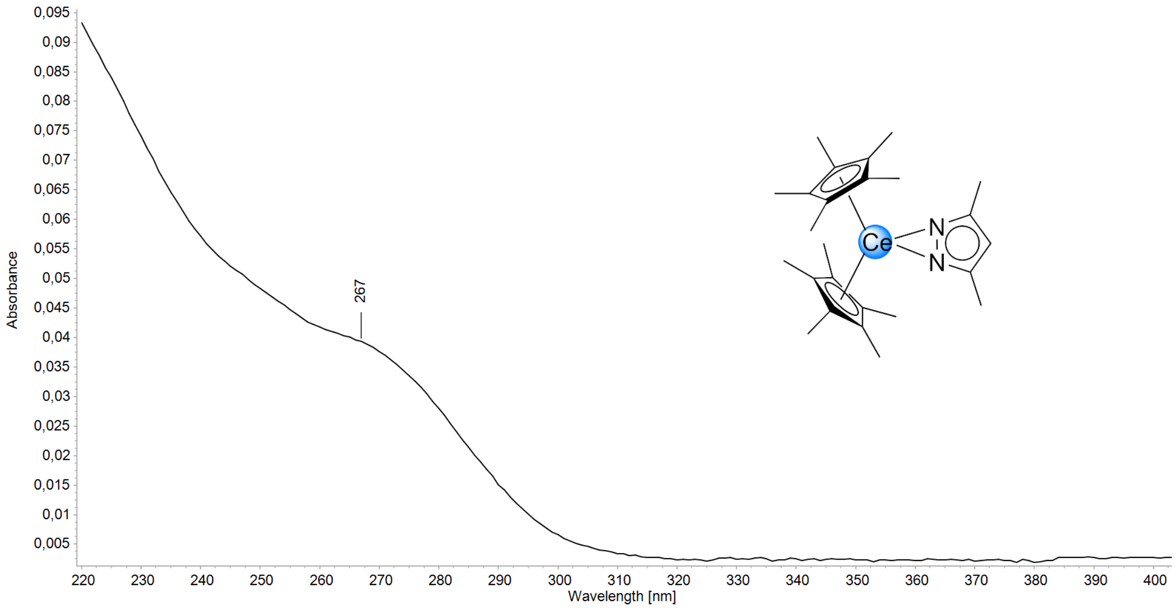

Figure S85. Absorption spectrum of a 10 µM solution of $Cp^{*}_{2}Ce(pz^{Me,Me})$ (**1**) in *n*-hexane.

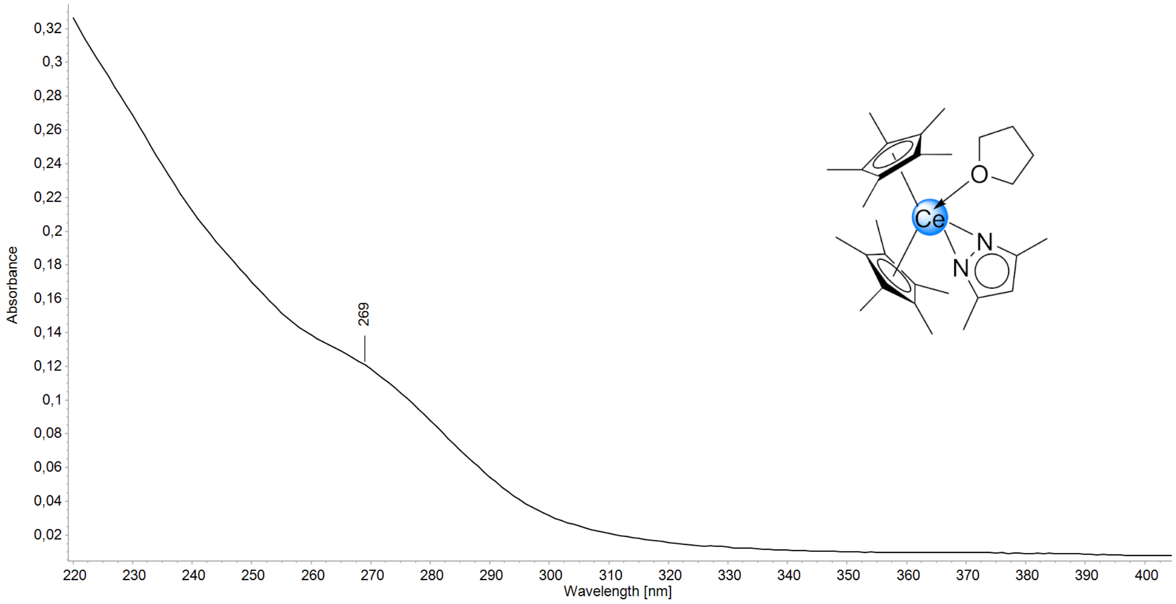

Figure S86. Absorption spectrum of a 10 µM solution of $Cp^{*}_{2}Ce(pz^{Me,Me})(thf)$ ($\mathbf{1^{thf}}$) in *n*-hexane.

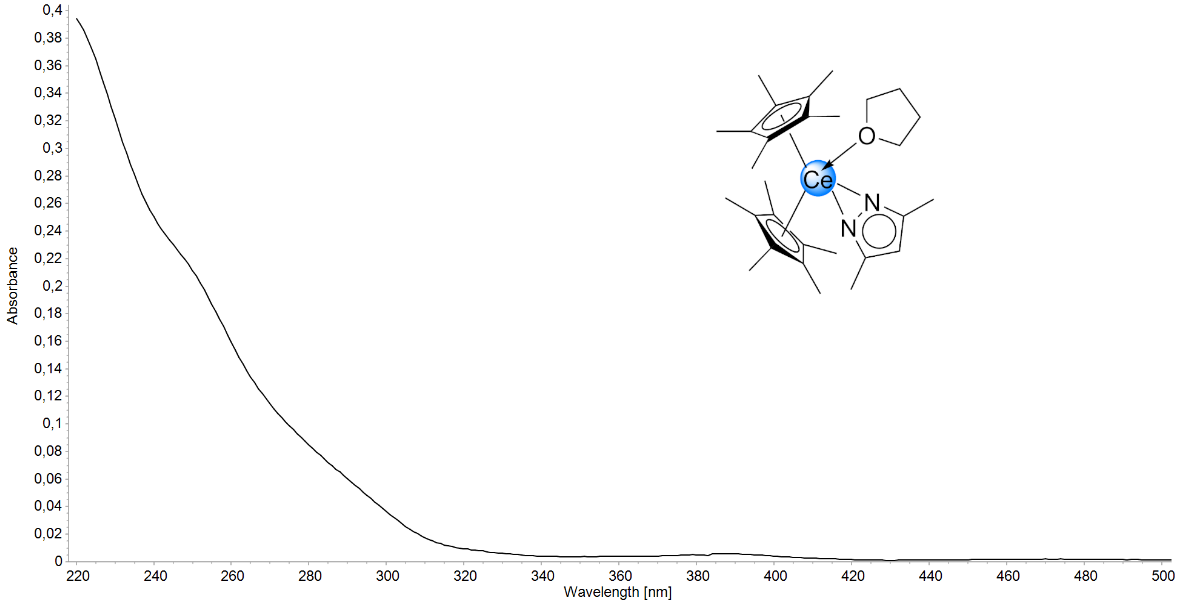

Figure S87. Absorption spectrum of a 10 μM solution of $Cp^*_2Ce(pz^{Me,Me})(thf)$ ($\mathbf{1^{thf}}$) in THF.

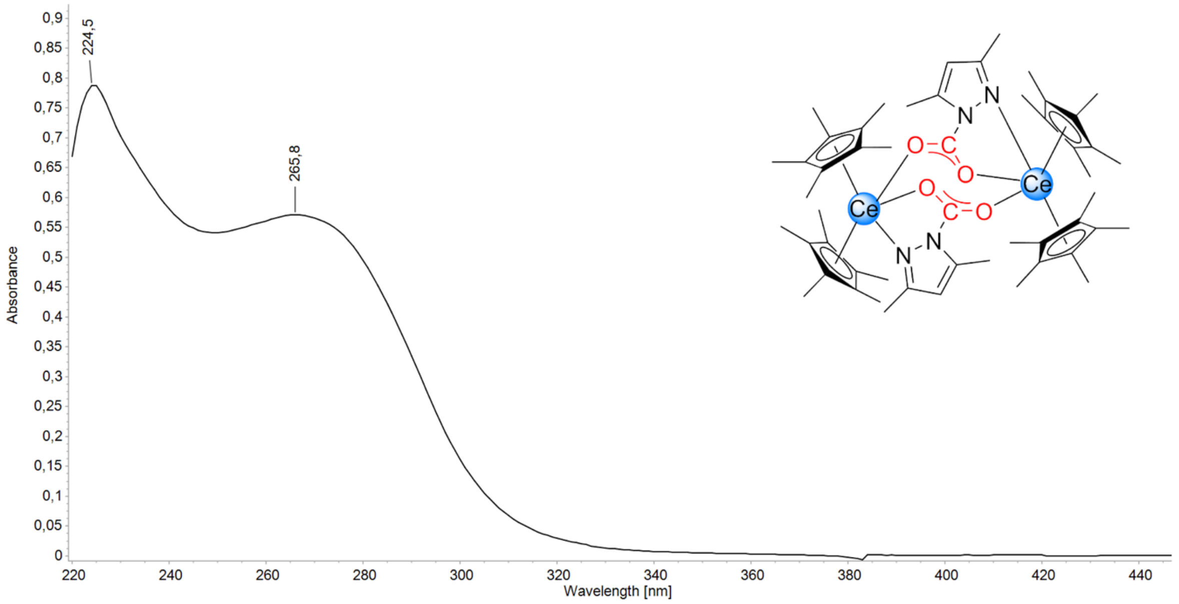

Figure S88. Absorption spectrum of a 100 μM solution of $[Cp^*_2Ce(pz^{Me,Me}\cdot CO_2)]_2$ (**2**) in THF.

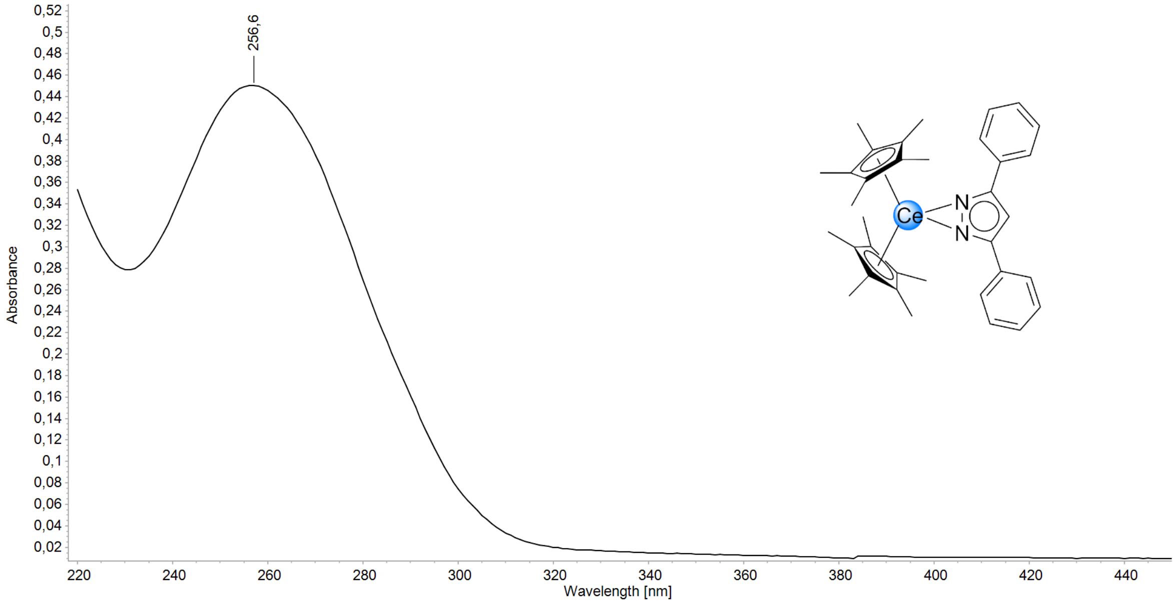

Figure S89. Absorption spectrum of a 10 μM solution of $Cp^{*}_{2}Ce(pz^{Ph,Ph})$ (**3**) in *n*-hexane.

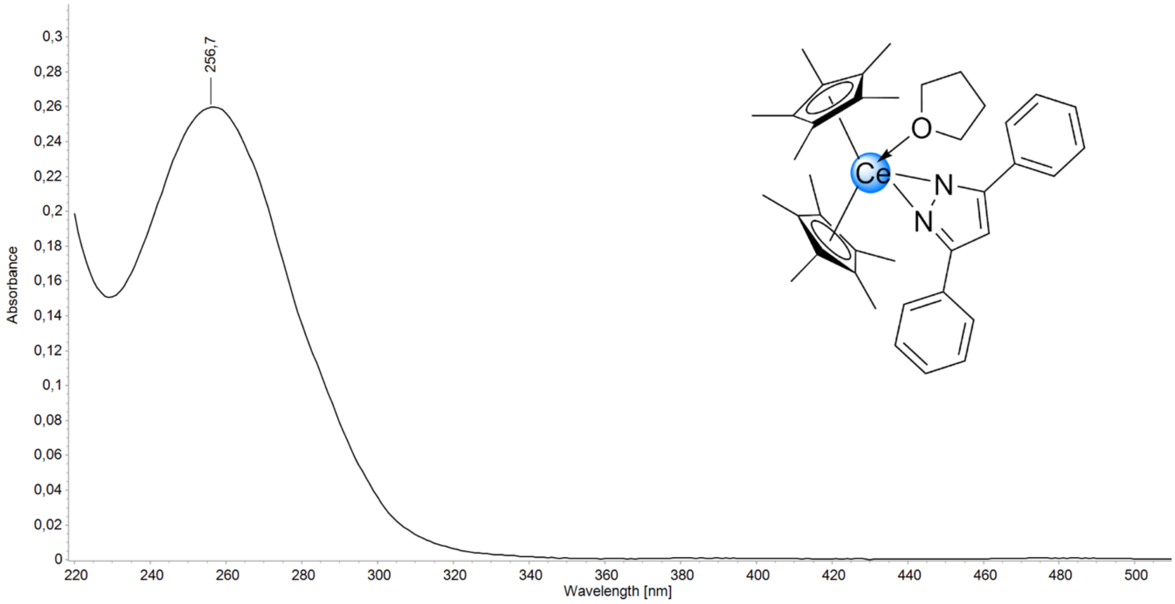

Figure S90. Absorption spectrum of a 10 μM solution of $Cp^{*}_{2}Ce(pz^{Ph,Ph})(thf)$ ($\mathbf{3^{thf}}$) in THF.

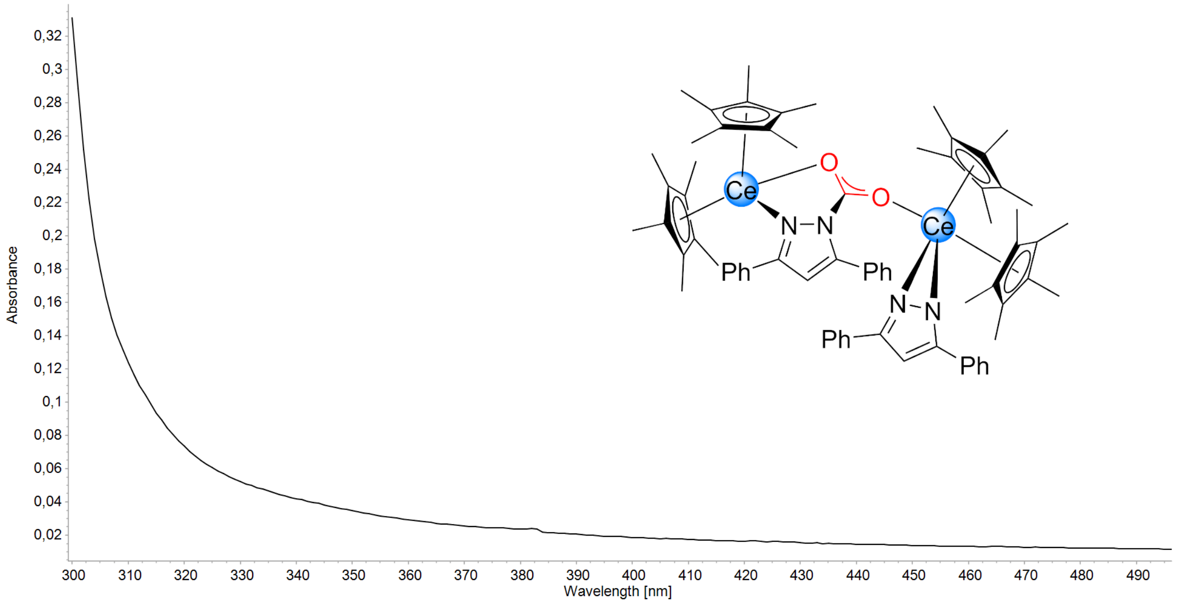

Figure S91. Absorption spectrum of a 100 µM solution of $Cp^{*}_{2}Ce(\mu\text{-}pz^{Ph,Ph}\cdot CO_{2})CeCp^{*}_{2}(pz^{Ph,Ph})$ (**4**) in toluene.

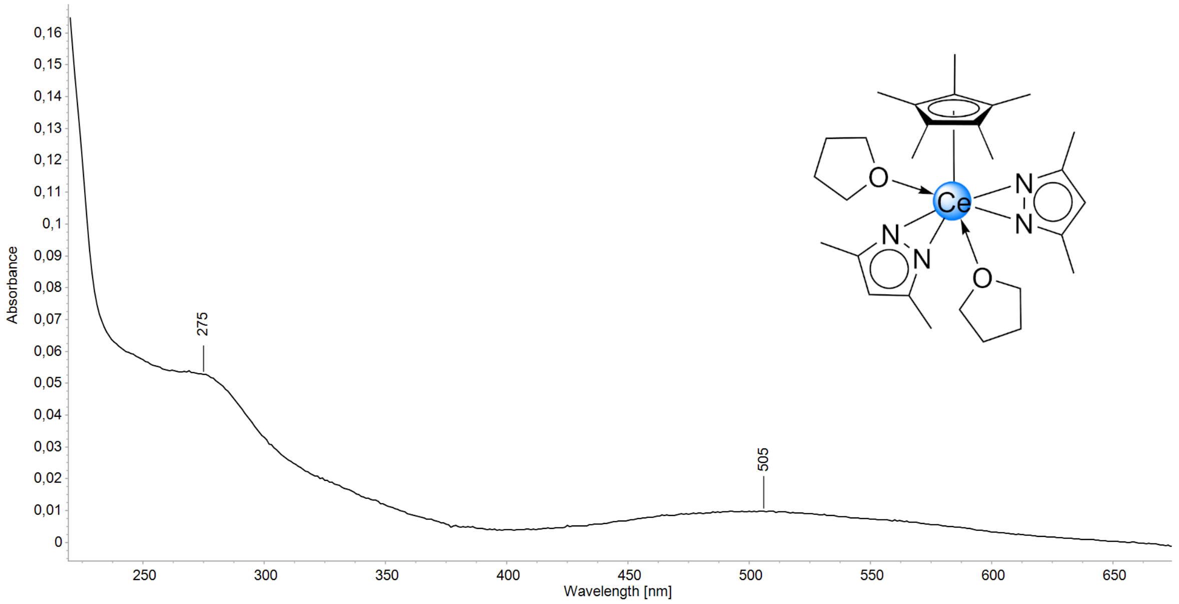

Figure S92. Absorption spectrum of a 10 µM solution of $Cp^{*}Ce(pz^{Me,Me})_{2}(thf)_{2}$ (**5**) in *n*-hexane.

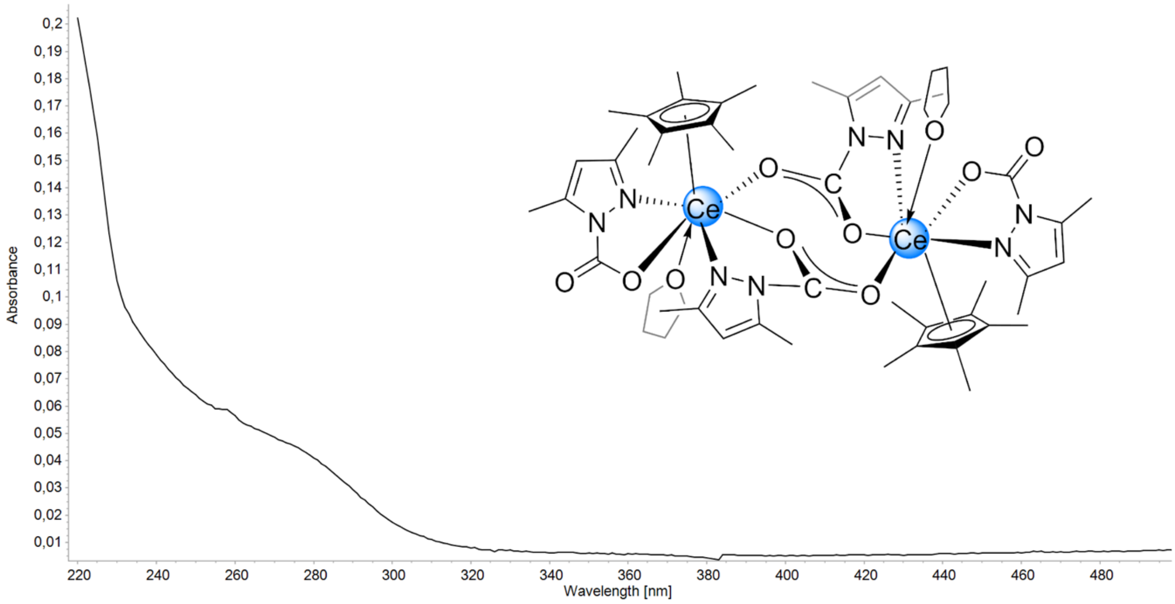

Figure S93. Absorption spectrum of a 20 µM solution of [Cp*Ce(pz^{Me,Me}·CO_2)(µ-pz^{Me,Me}·CO_2)(thf)]$_2$ (**6**) in THF.

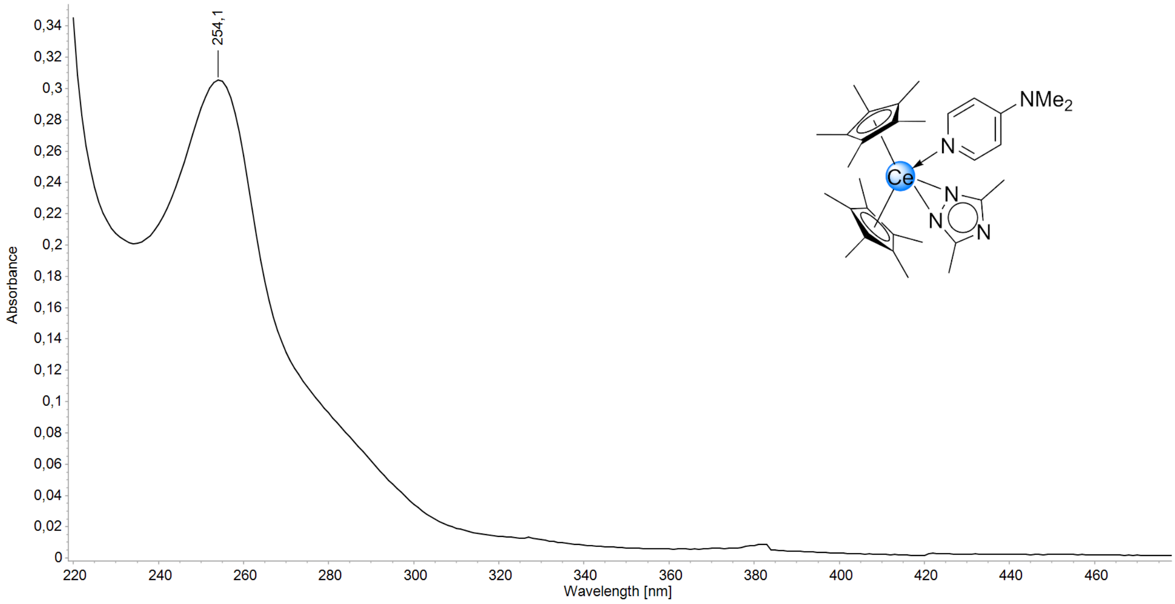

Figure S94. Absorption spectrum of a 10 µM solution of Cp*$_2$Ce(tz^{Me,Me})(dmap) (**7**) in THF.

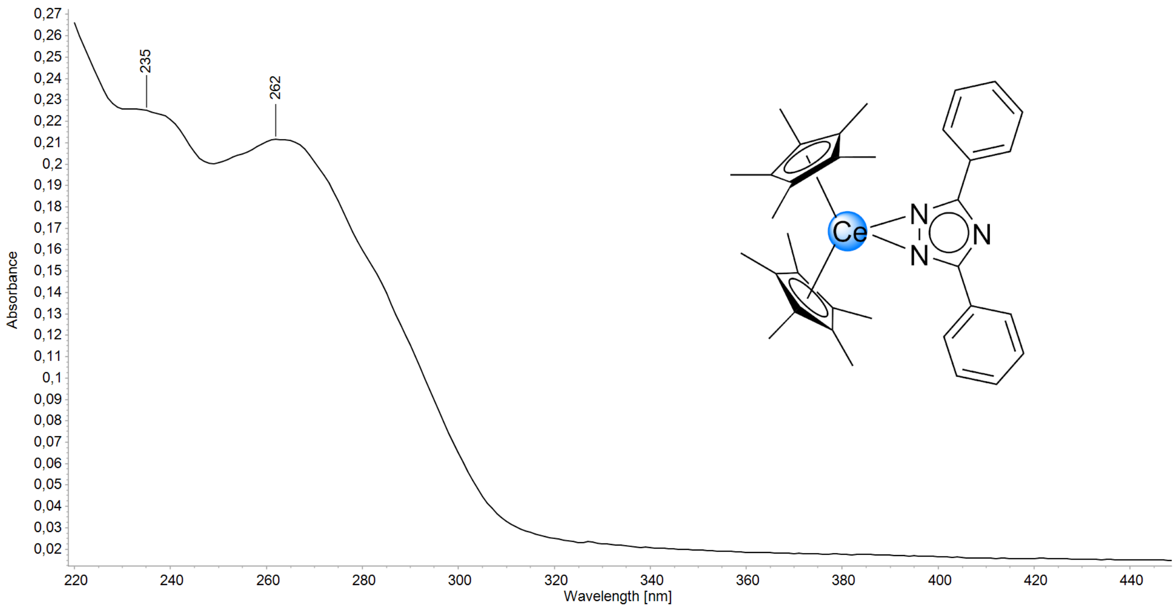

Figure S95. Absorption spectrum of a 10 µM solution of $Cp*_2Ce(tz^{Ph,Ph})$ (**8**) in *n*-hexane.

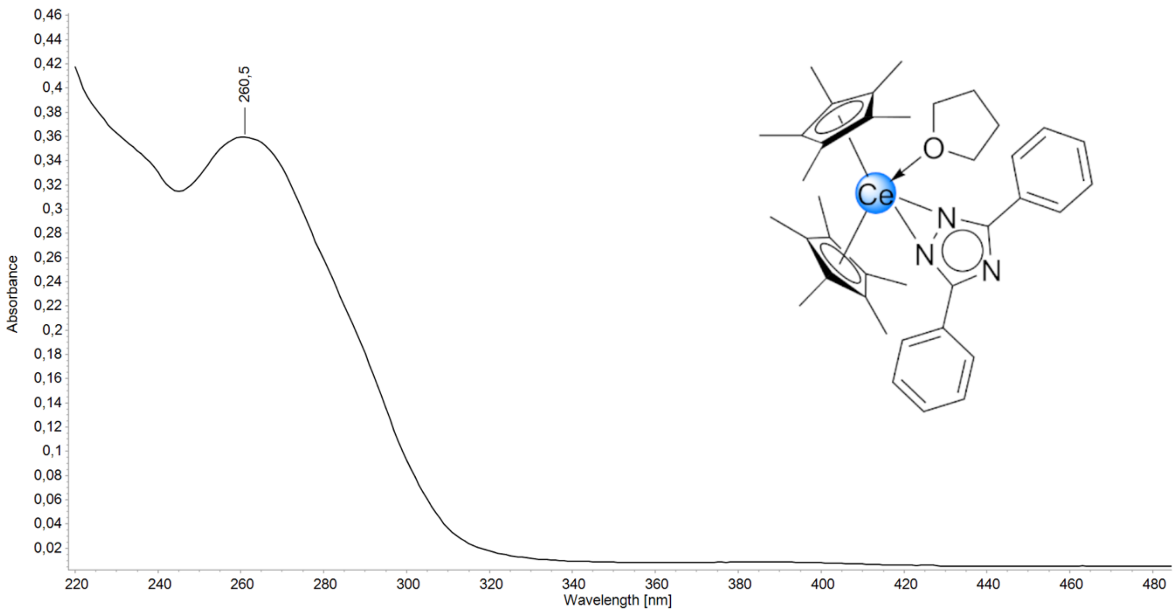

Figure S96. Absorption spectrum of a 10 µM solution of $Cp*_2Ce(tz^{Ph,Ph})$(thf) (**8**$^{\mathbf{thf}}$) in THF.

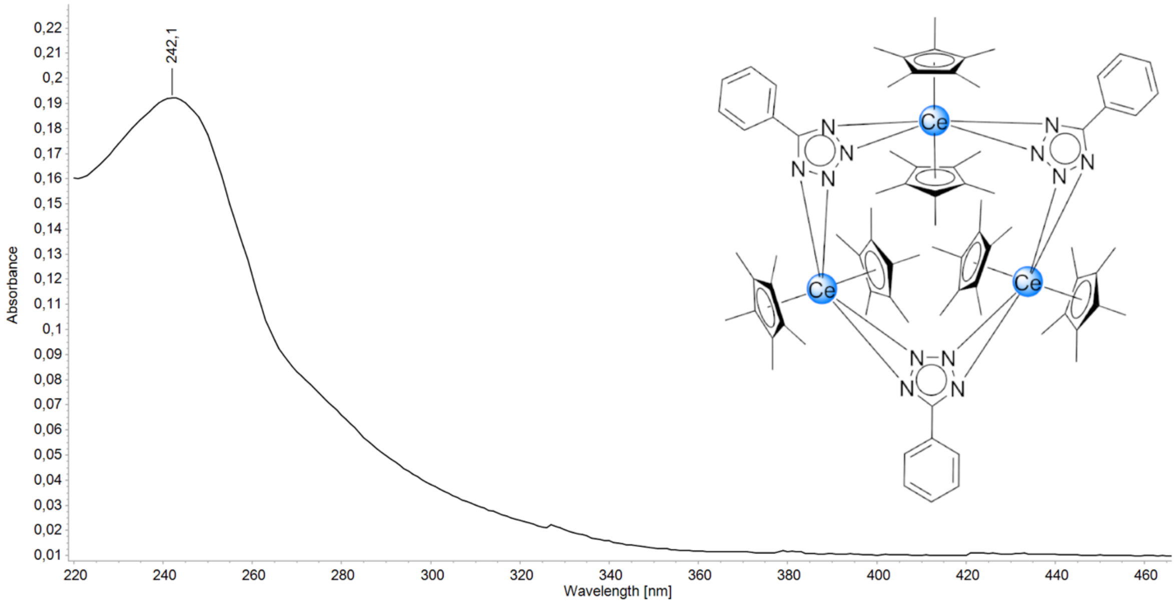

Figure S97. Absorption spectrum of a 2.5 µM solution of [Cp*$_2$Ce(tet^{Ph})]$_3$ (**9**) in *n*-hexane.

Cyclic Voltammetry Experiments

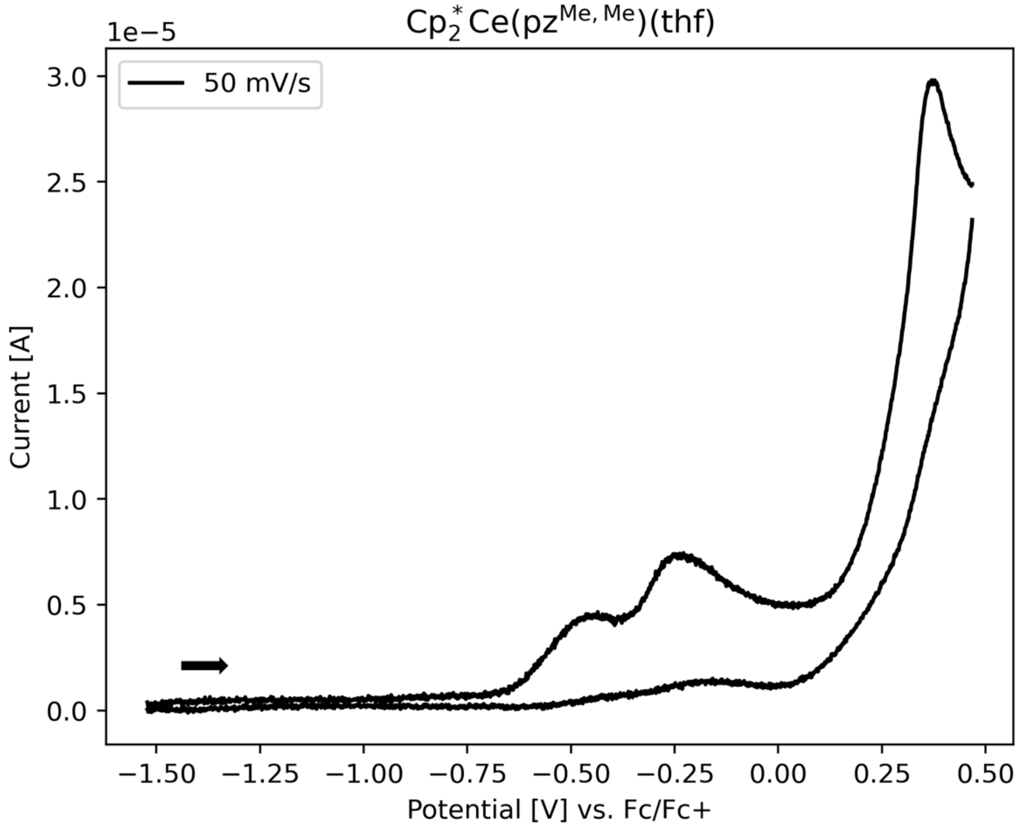

Figure S98. Cyclic voltammogram of $Cp^*{}_2Ce(pz^{Me,Me})(thf)$ ($\mathbf{1}^{\mathbf{thf}}$) vs Fc/Fc^+ in THF at a glassy-carbon electrode obtained at a scan rate of 50 mV/s. The arrow indicates the initial scan direction. The analyte concentration was 1 mM, and the electrolyte concentration was 0.1 M $[n\text{Pr}_4N][B(C_6H_3(CF_3)_2\text{-}3,5)_4]$.

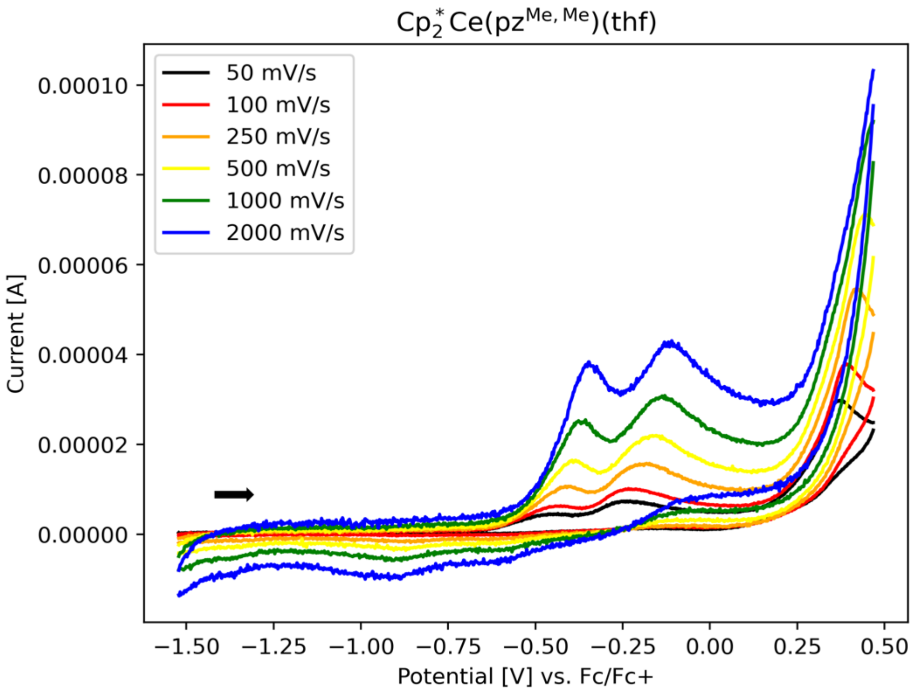

Figure S99. Cyclic voltammogram of Cp*$_2$Ce(pz^{Me,Me})(thf) ($\mathbf{1}^{\mathbf{thf}}$) vs Fc/Fc$^+$ in THF at a glassy-carbon electrode obtained at different scan rates. The arrow indicates the initial scan direction. The analyte concentration was 1 mM, and the electrolyte concentration was 0.1 M [*n*Pr$_4$N][B(C$_6$H$_3$(CF$_3$)$_2$-3,5)$_4$].

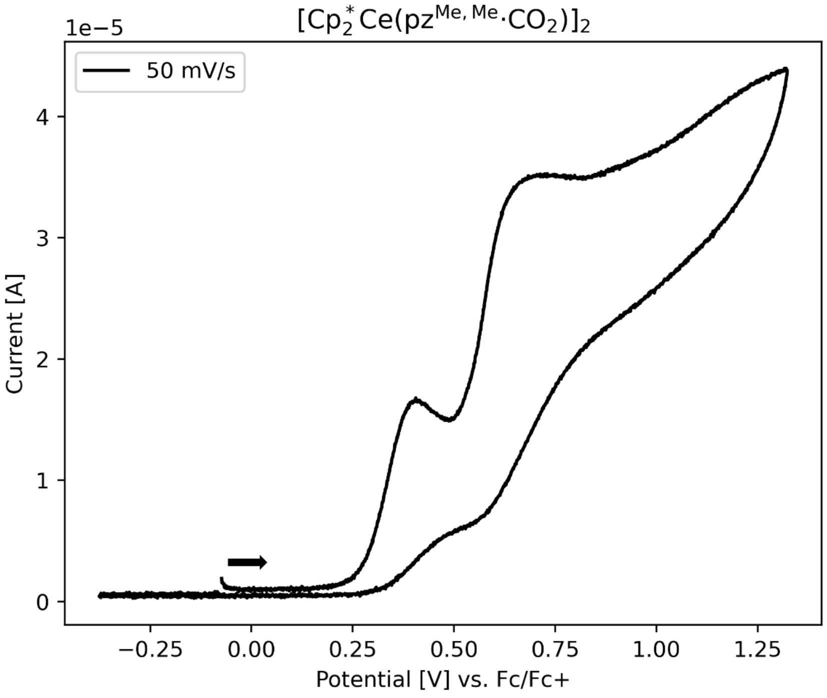

Figure S100. Cyclic voltammogram of [Cp*$_2$Ce(pz^{Me,Me}·CO$_2$)]$_2$ (**2**) vs Fc/Fc$^+$ in THF at a glassy-carbon electrode obtained at 50 mV/s. The arrow indicates the initial scan direction. The analyte concentration was 1 mM, and the electrolyte concentration was 0.1 M [*n*Pr$_4$N][B(C$_6$H$_3$(CF$_3$)$_2$-3,5)$_4$].

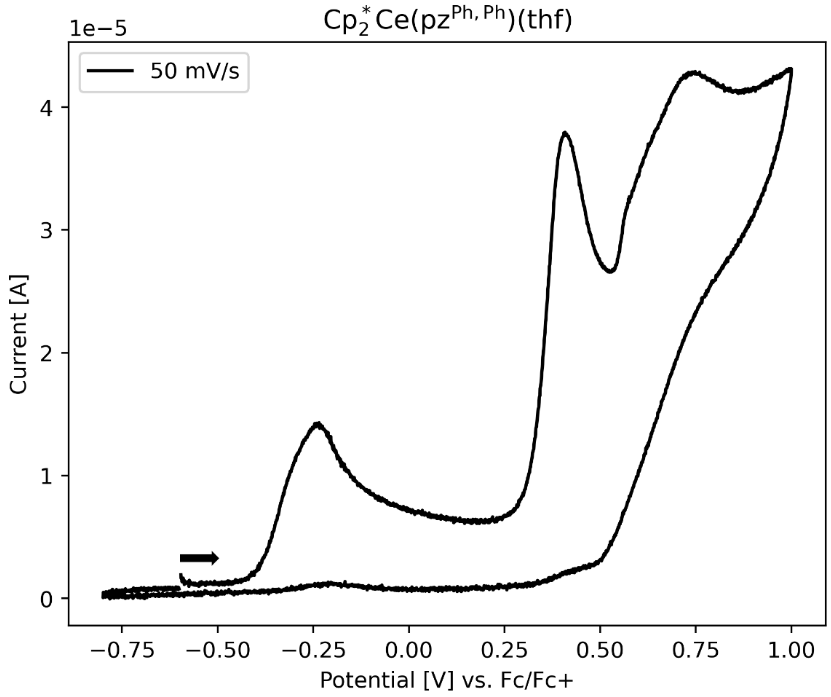

Figure S101. Cyclic voltammogram of $Cp*_2Ce(pz^{Ph,Ph})(thf)$ (**3**$^{\mathbf{thf}}$) vs Fc/Fc^+ in THF at a glassy-carbon electrode obtained at 50 mV/s. The arrow indicates the initial scan direction. The analyte concentration was 1 mM, and the electrolyte concentration was 0.1 M [nPr$_4$N][B($C_6H_3(CF_3)_2$-3,5)$_4$].

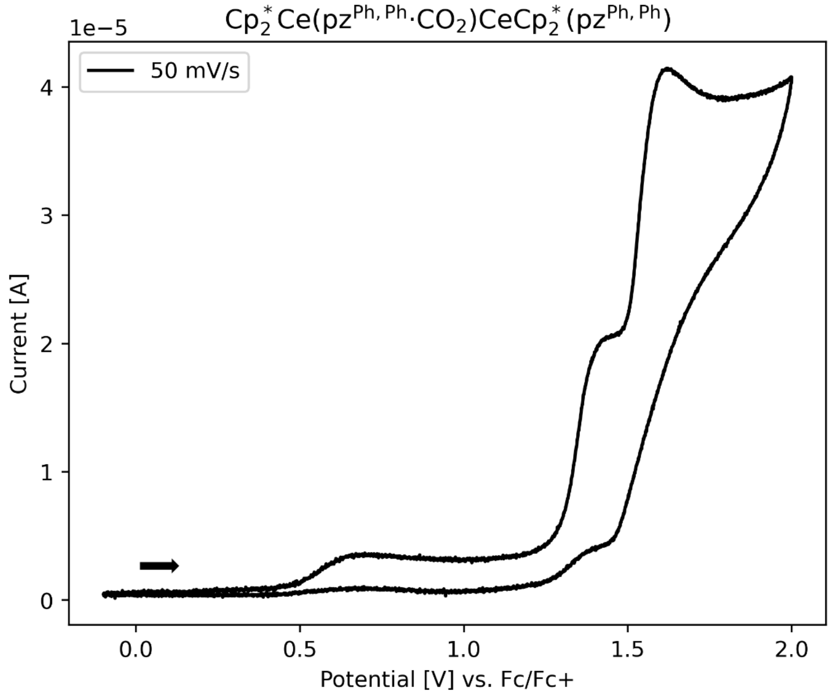

Figure S102. Cyclic voltammogram of $Cp*_2Ce(pz^{Ph,Ph}\cdot CO_2)CeCp*_2(pz^{Ph,Ph})$ (**4**) vs Fc/Fc^+ in THF at a glassy-carbon electrode obtained at 50 mV/s. The arrow indicates the initial scan direction. The analyte concentration was 1 mM, and the electrolyte concentration was 0.1 M [nPr$_4$N][B($C_6H_3(CF_3)_2$-3,5)$_4$].

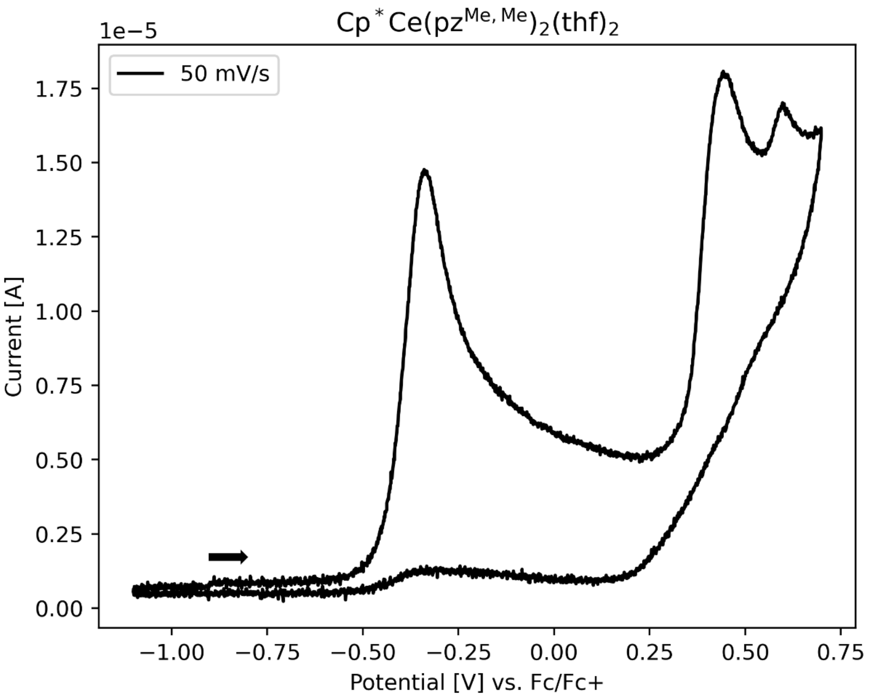

Figure S103. Cyclic voltammogram of $Cp^{*}Ce(pz^{Me,Me})_2(thf)_2$ (**5**) vs Fc/Fc^{+} in THF at a glassy-carbon electrode obtained at 50 mV/s. The arrow indicates the initial scan direction. The analyte concentration was 1 mM, and the electrolyte concentration was 0.1 M [*n*Pr_4N][$B(C_6H_3(CF_3)_2$-3,5$)_4$].

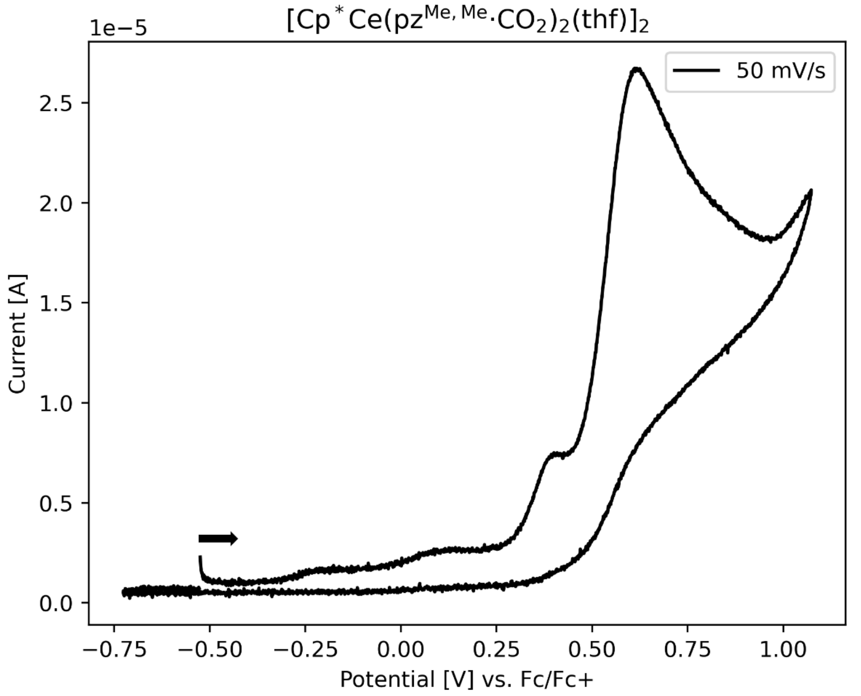

Figure S104. Cyclic voltammogram of $[Cp^{*}Ce(pz^{Me,Me}\cdot CO_2)_2(thf)]_2$ (**6**) vs Fc/Fc^{+} in THF at a glassy-carbon electrode obtained at 50 mV/s. The arrow indicates the initial scan direction. The analyte concentration was 1 mM, and the electrolyte concentration was 0.1 M [*n*Pr_4N][$B(C_6H_3(CF_3)_2$-3,5$)_4$].

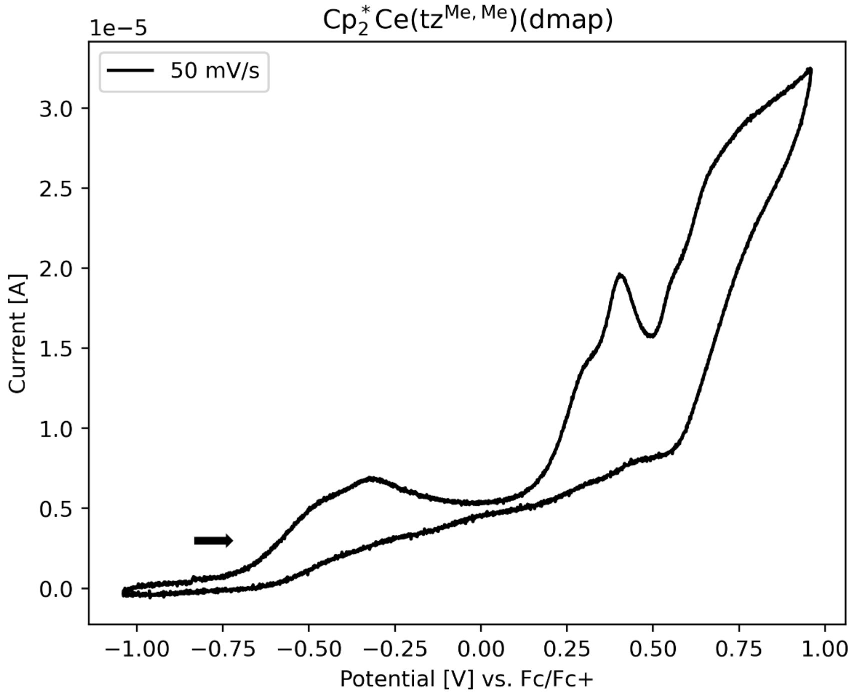

Figure S105. Cyclic voltammogram of Cp*$_2$Ce(tz^{Me,Me})(dmap) (**7**) vs Fc/Fc$^+$ in THF at a glassy-carbon electrode obtained at 50 mV/s. The arrow indicates the initial scan direction. The analyte concentration was 1 mM, and the electrolyte concentration was 0.1 M [*n*Pr$_4$N][B(C$_6$H$_3$(CF$_3$)$_2$-3,5)$_4$].

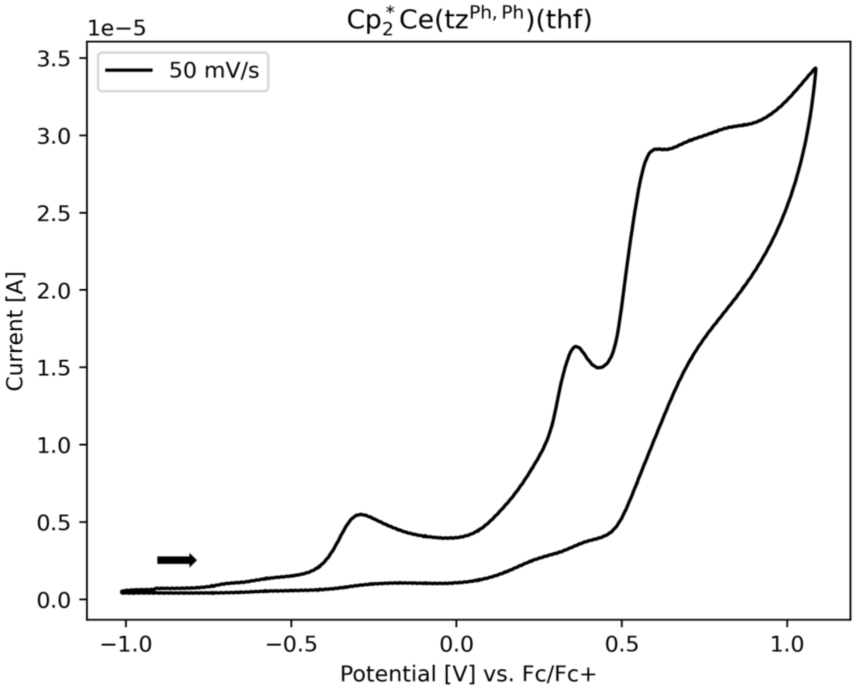

Figure S106. Cyclic voltammogram of Cp*$_2$Ce(tz^{Ph,Ph})(thf) (**8^{thf}**) vs Fc/Fc$^+$ in THF at a glassy-carbon electrode obtained at 50 mV/s. The arrow indicates the initial scan direction. The analyte concentration was 1 mM, and the electrolyte concentration was 0.1 M [*n*Pr$_4$N][B(C$_6$H$_3$(CF$_3$)$_2$-3,5)$_4$].

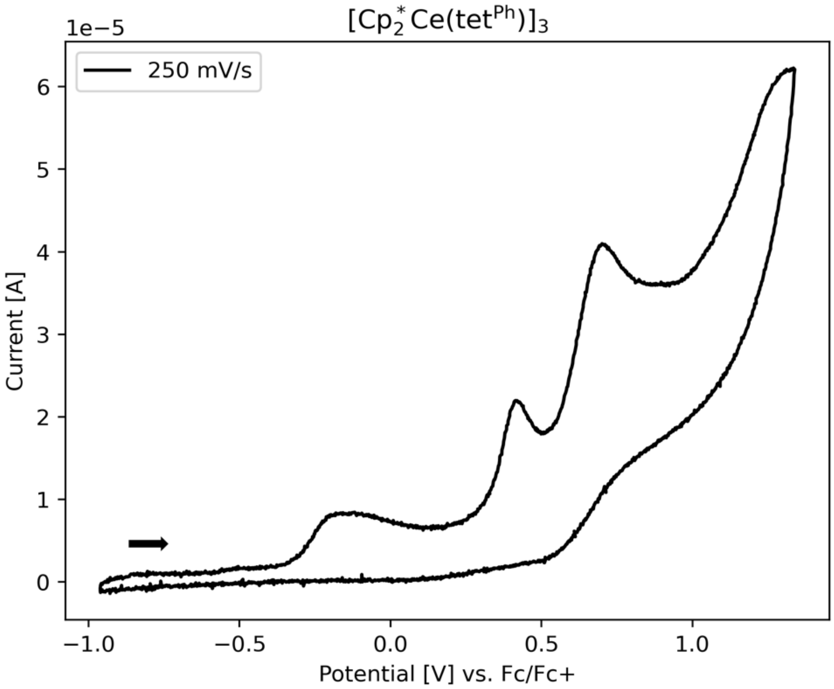

Figure S107. Cyclic voltammogram of [Cp*$_2$Ce(tet^{Ph})]$_3$ (**9-Ce**) vs Fc/Fc$^+$ in THF at a glassy-carbon electrode obtained at 250 mV/s. The arrow indicates the initial scan direction. The analyte concentration was 1 mM, and the electrolyte concentration was 0.1 M [*n*Pr$_4$N][B(C$_6$H$_3$(CF$_3$)$_2$-3,5)$_4$].

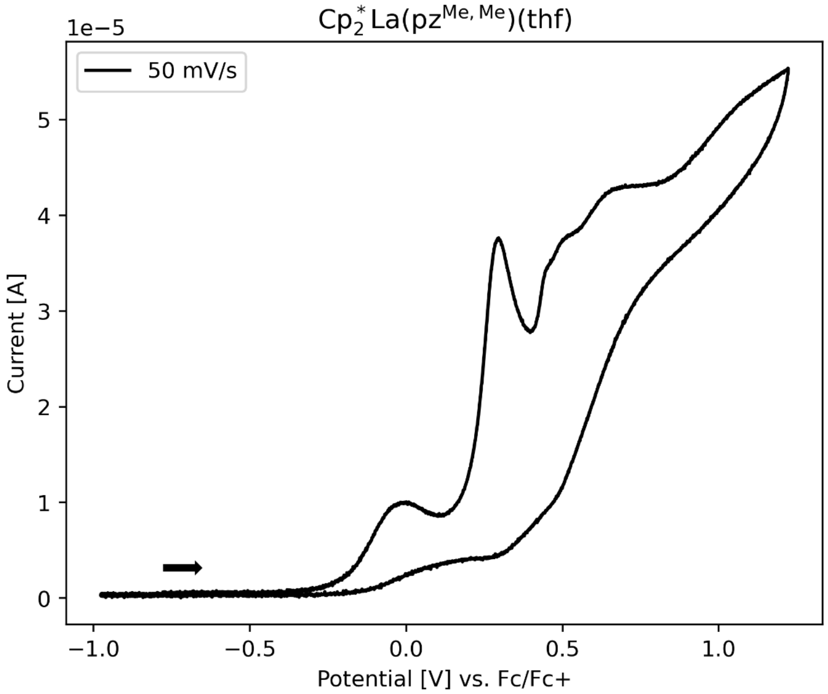

Figure S108. Cyclic voltammogram of Cp*$_2$La(pz^{Me,Me})(thf) (**1^{thf}-La**) vs Fc/Fc$^+$ in THF at a glassy-carbon electrode obtained at a scan rate of 50 mV/s. The arrow indicates the initial scan direction. The analyte concentration was 1 mM, and the electrolyte concentration was 0.1 M [*n*Pr$_4$N][B(C$_6$H$_3$(CF$_3$)$_2$-3,5)$_4$].

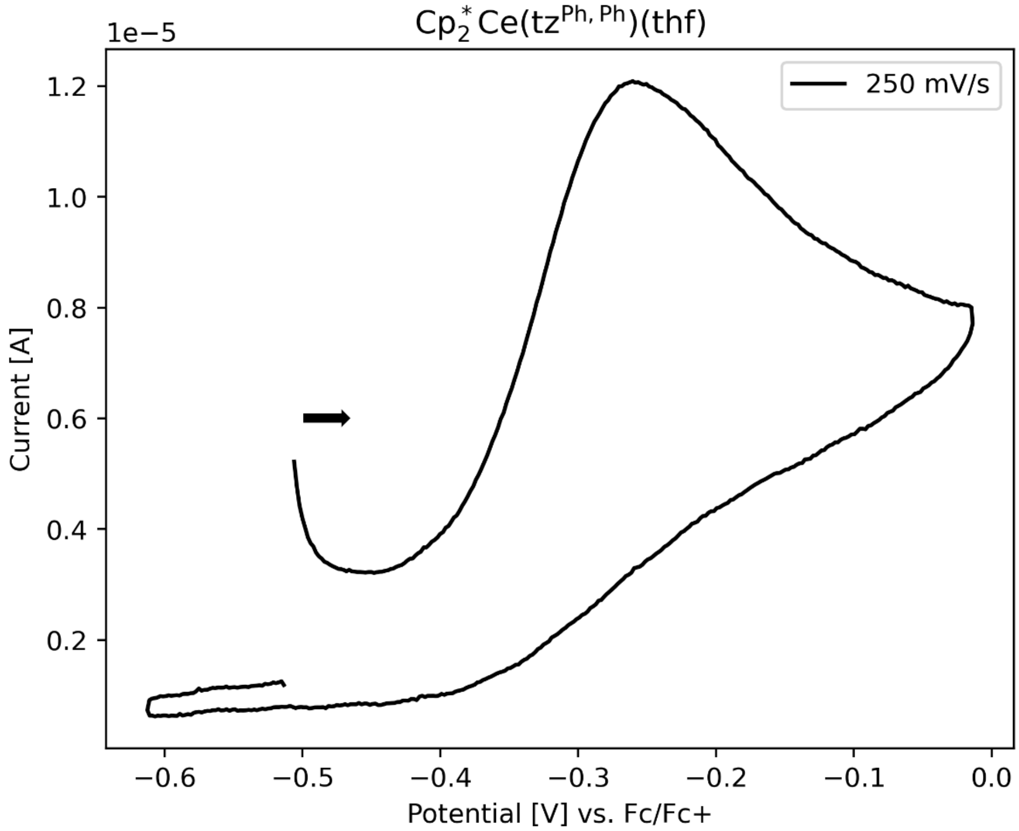

Figure S109. Cyclic voltammogram of Cp*$_2$Ce(tz^{Ph,Ph})(thf) (**8^{thf}**) vs Fc/Fc$^+$ in THF in the range between –0.6 V and 0 V at a glassy-carbon electrode obtained at 250 mV/s. The arrow indicates the initial scan direction. The analyte concentration was 1 mM, and the electrolyte concentration was 0.1 M [*n*Pr$_4$N][B(C$_6$H$_3$(CF$_3$)$_2$-3,5)$_4$].
